# Water compatible silica supported iron trifluoroacetate and trichloroacetate: as prominent and recyclable Lewis acid catalysts for solvent-free green synthesis of hexahydroquinoline-3-carboxamides†

**DOI:** 10.1039/d3ra03542e

**Published:** 2023-08-04

**Authors:** Dnyaneshwar Purushottam Gholap, Ramdas Huse, Sudarshan Dipake, M. K. Lande

**Affiliations:** a Department of Chemistry, Dr Babasaheb Ambedkar Marathwada University Aurangabad Maharashtra India mkl_chem@yahoo.com

## Abstract

Silica supported iron trifluoroacetate and iron trichloroacetate green Lewis acid catalysts were developed by a novel, cheap, environment-friendly approach and utilized in the synthesis of hexahydroquinoline-3-carboxamide derivatives. The structure and morphology of the prepared Lewis acid catalysts were studied by FTIR, PXRD, FE-SEM, HR-TEM, EDX, BET, TGA and NH_3_-TPD techniques. The present catalysts shows maximum conversion efficiency in hexahydroquinoline-3-carboxamide derivatives synthesis at 70 °C in solvent free reaction condition with best product yield in a short reaction time. Both catalysts are reusable and simple to recover, and perform meritoriously in water as well as in a variety of organic solvents. The key advantages of the current synthetic route are permitting of a variety of functional groups, quick reaction time, high product yield, mild reaction condition, recyclability of catalyst and solvent-free green synthesis. This makes it more convenient, economic and environmentally beneficial.

## Abstract 2

Silica supported iron trifluoroacetate and iron trichloroacetate green Lewis acid catalysts were developed by a novel, cheap, environment-friendly approach and utilized in the synthesis of hexahydroquinoline-3-carboxamide derivatives.

## Graphical abstract

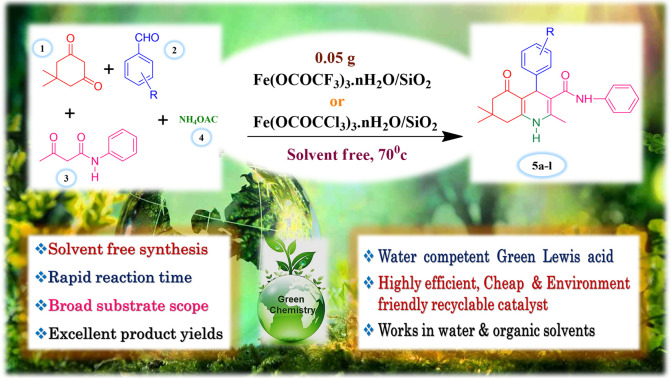

## 1. Introduction

Acid catalysts have created new horizons and milestones in the field of synthetic chemistry in recent decades.^1–3^ These catalysts are at the heart of many crucial industrial processes including biomass conversion,^4^ biodiesel production,^5^ polymer synthesis^6^ and different organic transformations,^7^ because of their great ability to accelerate reaction rates at low cost, excellent conversion, and product selectivity. Among all these acid catalysts, Lewis acids are considered as one of the supreme pillars and the most significant area of catalysts for organic synthesis. Lewis acid promoted reactions are very versatile because of their distinctive reactivity, selectivity, and benign reaction conditions.^8–10^ As a result, there is high demand for the advancement and designing of novel Lewis acids in synthetic organic chemistry to contribute towards the environmental sustainability and green chemistry. However, the use of heterogeneous lanthanide triflates, rare earth metal triflates and transition metal triflates in place of conventional homogeneous Lewis and Brønsted acid catalysts may become a more environment-friendly choice. In addition, the use of these heterogeneous Lewis acid catalysts in solvent-free environments is growing widely due to the many benefits, including a faster rate of reaction, less hazardous solvent pollution, reusability, air/water compatibility, low cost, and remarkable ability to suppress side reactions in substrates with acid sensitivity, which makes them valuable and helpful catalysts in synthetic processes.^11–13^

In recent times, many homogeneous and heterogenous supported green Lewis acid catalysts were designed and used in various chemical transformations. These catalysts include lanthanide triflates, rare earth metal triflates, transition metal triflates, trifluoroacetic acid or trichloroacetic acid, iron trifluoromethanesulfonate, iron trifluoroacetate, lanthanum trifluoroacetate and lanthanum trichloroacetate.^14–19^ Additionally, many mesoporous nano organosilica materials were utilized in different organic transformations which includes Pd-containing IL-based ordered nanostructured organosilica,^20^ a Pd-containing magnetic periodic mesoporous organosilica nanocomposite,^21^ a Cu-containing magnetic yolk–shell structured ionic liquid based organosilica nanocomposite,^22^ highly ordered mesoporous organosilica–titania with an ionic liquid framework,^23^ copper/IL-containing magnetic nanoporous MCM-41,^24^ core–shell structured Fe_3_O_4_@SiO_2_-supported IL/[Mo_6_O_19_],^25^ an ionic liquid/Mn complex immobilized on phenylene based periodic mesoporous organosilica,^26^ phenylene and isatin based bifunctional mesoporous organosilica supported Schiff-base/manganese complexes,^27^ amine-functionalized ionic liquid-based mesoporous organosilica,^28^ magnetic ethylene-based periodic mesoporous organosilica supported palladium,^29^ phenylene-based periodic mesoporous organosilica supported melamine,^30^ a magnetic silica nanocomposite supported W_6_O_19_/amine,^31^ phenyl and ionic liquid based bifunctional periodic mesoporous organosilica supported copper,^32^ magnetic silica supported propylamine/H_3_PW_12_O_40_.^33^

The green Lewis acids are highly efficient chemo, regio and stereoselective catalysts for widespread series of organic reactions including Diels–Alder reaction, aldol condensation, Friedel–Crafts alkylation, and acylation reaction, Baylis–Hillman reaction, radical addition, Michael reactions, Mannich reaction, alkene alkylation and dimerization, heterocyclic molecule synthesis, Reformatsky reaction, aromatic nitration, sulphonation and bromination reactions, rearrangement reactions, and many multicomponent, cyclization and ring-opening reactions.^34^ Herein, we have introduced silica-supported iron trifluoroacetate and iron trichloroacetate catalysts as a competent alternative to the Lanthanide triflates, rare earth metal triflates and transition metal triflate catalysts. These newly developed green Lewis acid catalysts are stable, nonhygroscopic, moisture insensitive, work as Lewis acid in aqueous and in organic medium, less expensive, quickly recovered and reused in reactions by retaining catalytic activity.

Heterocycles developed *via* Hantzsch syntheses, such as 1,4-dihydropyridine, polyhydroquinoline, and acridine, have gained much attention due to the presence of important biological activities. They have played a crucial role in the development of a broad range of heterocyclic molecules for medicinal use. They have pharmacological effects, such as vasodilators, antihypertensive, bronchodilators, anti-atherosclerotic, hepatoprotective, anticancer, anti-mutagenic, neuroprotective, and anti-diabetic characteristics.^35,36^ 1,4-DHPs, which are 1,4-substituted, form a significant family of Ca^2+^ channel blockers^37^ and are one of the most significant classes of medications used to treat cardiovascular disease.^38^ Furthermore, quinolines, particularly 1,4-DHPs have a number of pharmacological activities, including antianginal, anti-inflammatory action, anti-tumour, anti-tubercular activity, analgesic activity, and antithrombotic. Additionally, polyhydroquinoline heterocycles have also shown a number of medicinal applications, including as a neuroprotectant and as a cerebral anti-ischemic action in the management of Alzheimer's disease.^39^ In this context, the synthesis of a polyhydroquinoline scaffold still has attracted considerable attention. Acetoacetanilide is a crucial building component in the synthesis of these heterocyclic compounds. A literature survey of various methods for the polyhydroquinoline-3-carboxamides synthesis *via* a four-component reaction including acetoacetanilide, aromatic aldehydes, dimedone, and ammonium acetate reveals that many of these procedures suffered from complex synthetic paths, harsh reaction medium, longer reaction time, low product yield and non-recyclable catalyst. As a result, simple, efficient, environment-friendly, and versatile synthetic protocols are still in demand. Multicomponent reactions (MCRs) offer a handy method for the fast synthesis of complex molecules from easy beginning substances, without isolating intermediates.^40^ Compared to multistep synthesis, the MCRs have grown significantly in relevance to medicinal and organic chemistry because they minimize the use of catalysts and solvents, allowing for the reduction of waste, time, effort, and expense.^41–43^

In this regard, we have developed a new, efficient and green synthetic route for the synthesis of hexahydroquinoline-3-carboxamides from aromatic aldehyde, dimedone, acetoacetanilide, ammonium acetate. The solvent-free one pot condensation at 70 °C gives a higher yield of products with high catalyst recovery following simple reaction workup procedure. The present Lewis acid catalysts employed for the hexahydroquinoline-3-carboxamides synthesis show magnificent catalytic efficiency under solvent-free conditions in a short time.

## 2. Experimental section

### 2.1 Materials and methods

All the chemicals and solvents were purchased from Sigma-Aldrich and Molychem companies of high purity and used directly in the reaction without any further purification. The powder XRD was obtained with a well-calibrated instrument X-ray diffraction (XRD, Siemens D-5005 diffractometer) a Rigaku MiniFlex diffractometer having the Cu Kα (*λ* = 1.54 Å) line with radiation ranges from 5° and 60° (2*θ* values). The FTIR spectra were obtained using a Shimadzu FTIR 8300 and Bruker ALPHA (Eco-ATR) spectrophotometer. The elemental composition was carried *via* object 8724. The specific surface area and pore volume were obtained on Quantachrome instrument v5.2 with Brunauer–Emmett–Teller method. The structural morphology and elemental composition of the catalyst was determined by field emission SEM images and EDX using instrument Nova Nano SEM NPEP303 and high-resolution transmission electron microscopy images was captured on an instrument JEOL JEM 2100 Plus microscope. The acid strength and acid quantity were determined using NH_3_-TPD analysis on Microtrac MRB BELCAT II instrument. All the organic transformation progress was preliminary monitored by thin-layer chromatography (TLC). The ^1^H NMR spectra (400 MHz) and ^13^C NMR spectra (100 MHz) were run on a Bruker (400 MHz) spectrometer using d_6_-DMSO solvent and tetramethyl silane (TMS) as an internal reference. The melting points of all organic derivatives were recorded on the digital melting point apparatus.

### 2.2 Synthesis of iron trifluoroacetate (Fe(OCOCF_3_)_3_·*n*H_2_O) and trichloroacetate (Fe(OCOCCl_3_)_3_·*n*H_2_O)

**Scheme 1:**
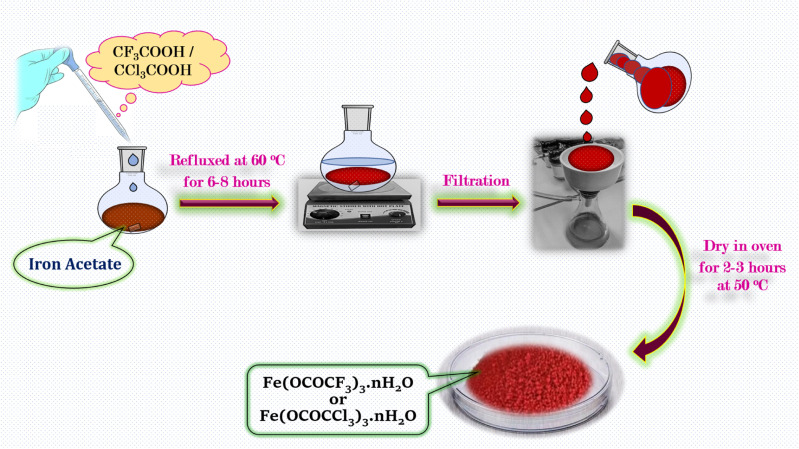
Schematic illustration for the synthetic procedure of iron trifluoroacetate (Fe(OCOCF_3_)_3_·*n*H_2_O) and trichloroacetate (Fe(OCOCCl_3_)_3_·*n*H_2_O) green Lewis acid catalyst.

The iron trifluoroacetate and iron trichloroacetate were prepared *via* new and novel method^14^ by the direct reaction between iron(iii) acetate (2 g) and an excess quantity of trifluoroacetic acid/trichloroacetic acid (9 g) respectively in 1 : 3 equivalent ratio. The reaction mixture was heated at 60 °C for 6–8 hours. The reddish-brown product formed in the reaction was separated *via* vacuum filtration for the removal of unreacted acid and acetic acid which obtained as a side product in the reaction. At the end, product was washed with *n*-hexane and dried in an oven at 50 °C for 2–3 hours (Scheme 1).

### 2.3 Synthesis of silica supported iron trifluoroacetate (Fe(OCOCF_3_)_3_·*n*H_2_O/SiO_2_) and trichloroacetate (Fe(OCOCCl_3_)_3_·*n*H_2_O/SiO_2_) Lewis acid catalyst

**Scheme 2:**
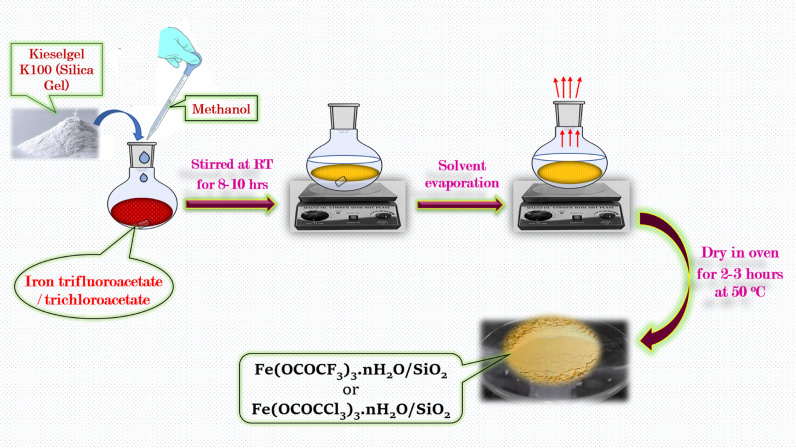
Schematic illustration for the synthetic procedure of silica supported iron trifluoroacetate (Fe(OCOCF_3_)_3_·*n*H_2_O/SiO_2_) and trichloroacetate (Fe(OCOCCl_3_)_3_·*n*H_2_O/SiO_2_) green Lewis acid catalyst.

The silica supported iron trifluoroacetate and iron trichloroacetate Lewis acids were prepared *via* literature known procedures^14^ by the slight modification in the procedure. The synthesized iron trifluoroacetate/trichloroacetate Lewis acid (2 g) was poured into methanol (90 ml) in 250 ml round bottom flask and reaction mixture was stirred for 10–15 minutes at RT. Further, the supporting material Kieselgel K100 or silica gel (20 g), was added to this reaction mixture and the resultant slurry of the reaction mixture was stirred finely for 8–10 hours at RT. Finally, the solvent in the reaction mixture was evaporated and then solid porous form supported Lewis acid catalyst was dried in a vacuum desiccator for 2–3 hours to obtained its anhydrous form (Scheme 2).

### 2.4 General procedure for hexahydroquinoline-3-carboxamides derivatives synthesis

A mixture of benzaldehyde (1 mmol), dimedone (1 mmol), acetoacetanilide (1 mmol), ammonium acetate (1.2 mmol) and silica supported iron trifluoroacetate or iron trichloroacetate catalyst (50 mg or 0.05 g) were stirred at 70 °C for 08 minutes under solvent-free condition. Thin layer chromatography was used to keep track of the reaction progress. After the end of reaction, the reaction mixture was diluted with hot ethanol (15 ml) and it was then filtered to remove the catalyst. The filtrate of reaction mixture was then added to crushed ice to produce a crude solid product, which was then filtered and recrystallized with the use of hot ethanol to produce pure crystals. The recovered catalyst was cleaned with ethanol and prepared for reuse by being dried in a vacuum desiccator for 2–3 hours.

## 3. Result and discussion

### 3.1 Catalyst characterization

**Fig. 1:**
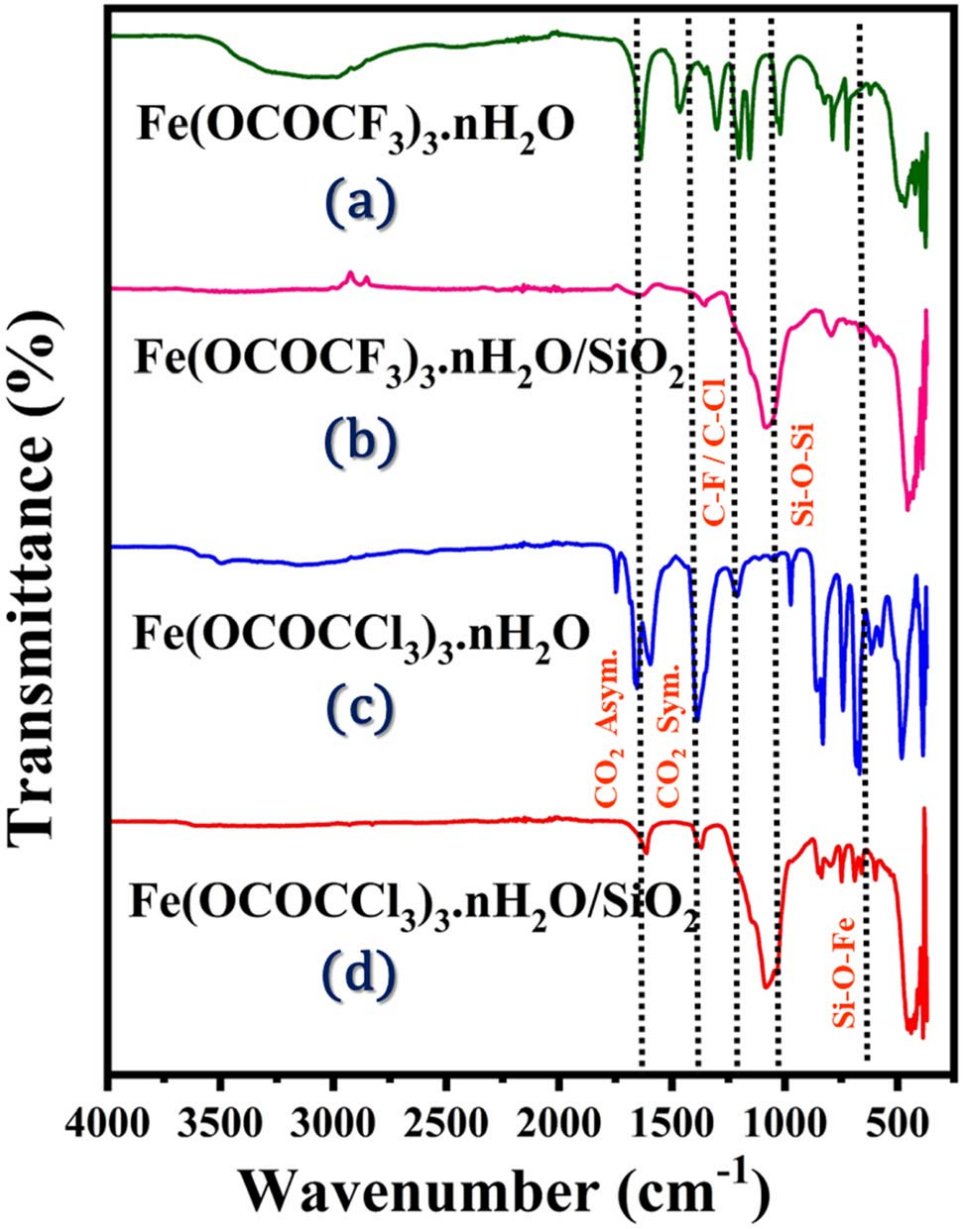
FTIR spectrum of Lewis acid catalysts (a) Fe(OCOCF_3_)_3_·*n*H_2_O (b) Fe(OCOCF_3_)_3_·*n*H_2_O/SiO_2_ (c) Fe(OCOCCl_3_)_3_·*n*H_2_O (d) Fe(OCOCCl_3_)_3_·*n*H_2_O/SiO_2_.

Fundamentally, FTIR spectroscopy was utilized for the investigation of primary structure of iron trifluoroacetate (Fe(OCOCF_3_)_3_·*n*H_2_O), iron trichloroacetate (Fe(OCOCCl_3_)_3_·*n*H_2_O), silica-supported iron trifluoroacetate (Fe(OCOCF_3_)_3_·*n*H_2_O/SiO_2_) and iron trichloroacetate (Fe(OCOCCl_3_)_3_·*n*H_2_O/SiO_2_) Lewis acid catalyst series depicted in Fig. 1. According to the literature, primitive structural information of currently synthesized Lewis acids is found in their FTIR spectrum. These catalysts show five characteristic frequencies in the FTIR spectrum. The spectrum at 1637 and 1656 cm^−1^ corresponds to a CO_2_ asymmetric vibration in unsupported iron trifluoroacetate and trichloroacetate catalysts respectively. Another distinctive band in respective catalysts at 1462 and 1444 cm^−1^, represents a CO_2_ symmetric vibration. While iron trifluoroacetate and trichloroacetate supported by silica exhibit an asymmetric CO_2_ vibration band at 1631 and 1622 cm^−1^ and CO_2_ symmetric vibration band at 1359 and 1377 cm^−1^. The interaction of silica and iron trifluoroacetate or iron trichloroacetate led to this alteration of vibrational value in the silica supported catalyst compared to unsupported catalyst.

In addition, the doublet spectra at 1155, 1125, and 1205 cm^−1^ is assigned to the C–F, C–Cl, and C–O bond vibrations of the trifluoroacetate or trichloroacetate groups. Further, a set of vibrational bands in the 825 and 839 cm^−1^ corresponding to δasCF_3_ and δasCCl_3_ mode of free CF_3_COO^−^ and CCl_3_COO^−^ functional moieties.^14,44^ Moreover, FTIR spectrum of the silica-supported catalyst provide a lower shift and revealed two new sharp bands appeared at 1080 cm^−1^ and 684 cm^−1^, which were attributed to (Si–O–Si) and (Fe–O–Si) bond stretching vibrations. This provides more conclusive evidence of the successful functionalization and stronger electrostatic interaction of iron trifluoroacetate and trichloroacetate with the mesoporous silica support material.

**Fig. 2:**
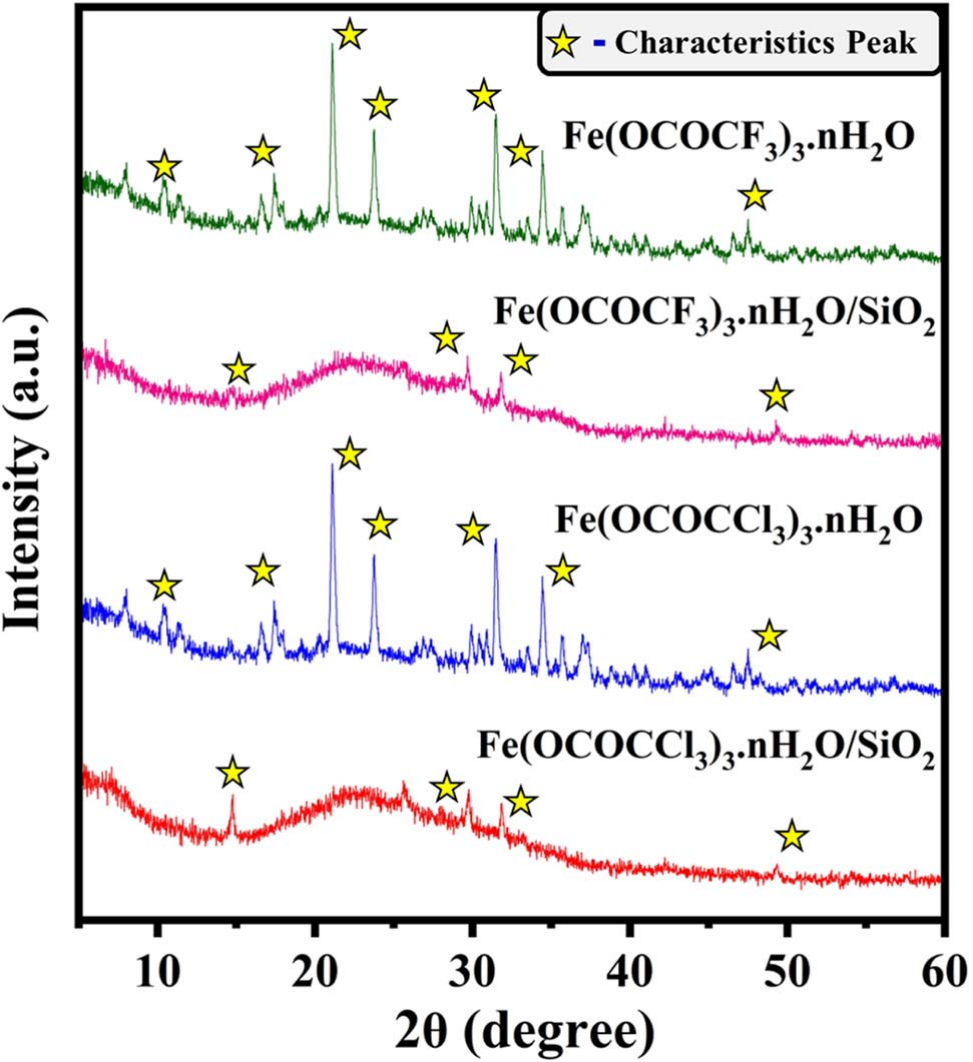
XRD spectrum of Lewis acid catalysts (a) Fe(OCOCF_3_)_3_·*n*H_2_O (b) Fe(OCOCF_3_)_3_·*n*H_2_O/SiO_2_ (c) Fe(OCOCCl_3_)_3_·*n*H_2_O (d) Fe(OCOCCl_3_)_3_·*n*H_2_O/SiO_2_.

The powder X-ray diffractometric (PXRD) analysis utilized to investigate the fine distribution and formation of iron trifluoroacetate and trichloroacetate functionalities on silica-supporting material. The PXRD spectrum of the bulk unsupported iron trifluoroacetate, iron trichloroacetate and their silica-supported forms were depicted in Fig. 2. The characteristic diffraction peaks of the bulk unsupported iron trifluoroacetate and trichloroacetate mostly include 2*θ* degree value = 10, 17, 21, 23, 31, 34, and 47.^45–48^ In addition, iron trifluoroacetate and trichloroacetate supported over silica exhibit modified and prominent distinctive diffraction peaks at 2*θ* degree value = 14, 29, 31, and 49. Moreover, in contrast to the sharp diffraction spectrum observed in unsupported iron trifluoroacetate and trichloroacetate, the broad humped diffraction spectrum was seen in silica-supported iron trifluoroacetate and trichloroacetate Lewis acids. This clearly demonstrates the disturbance in the original crystallinity of supported Lewis acid catalysts. This variation in the crystallinity of silica-supported Lewis acid is due to the strong electrostatic interaction between iron trifluoroacetate/trichloroacetate and mesoporous silica, which further transforms the supported Lewis acids into an amorphous nature.^14,49^ This result provides strong confirmation about the fine dispersion of iron trifluoroacetate and trichloroacetate over mesoporous silica material in the present silica-supported Lewis acids, [Fe(OCOCF_3_)_3_·*n*H_2_O/SiO_2_] and [Fe(OCOCCl_3_)_3_·*n*H_2_O/SiO_2_].

**Fig. 3:**
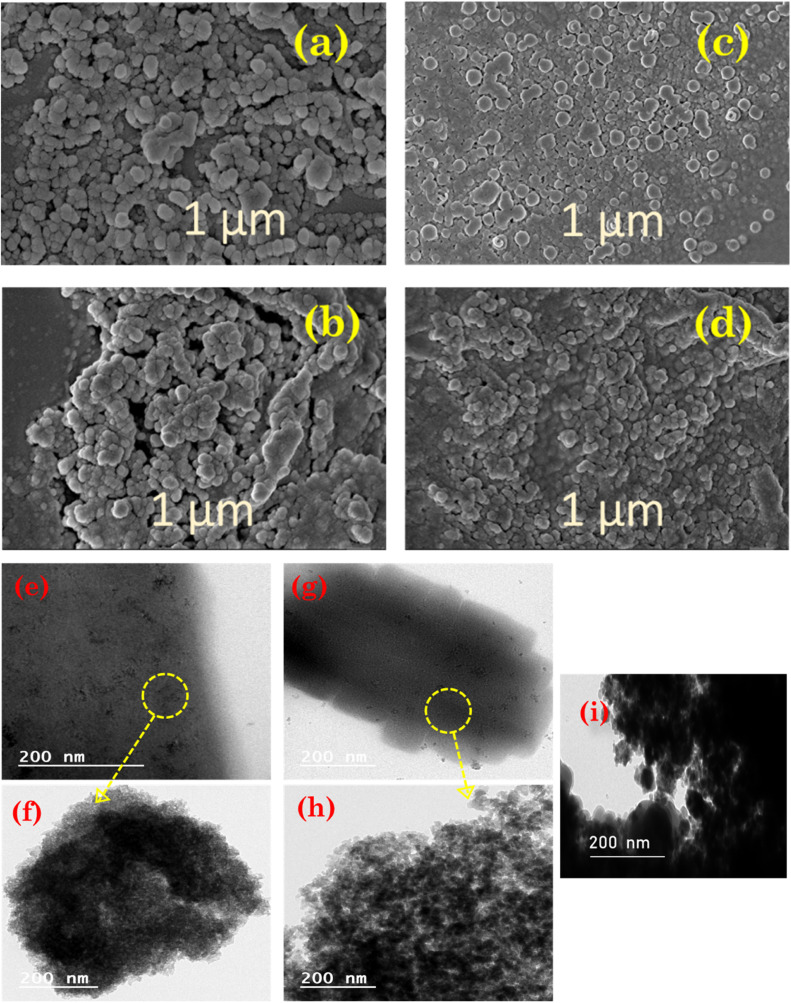
FE-SEM images of (a) bulk iron trifluoroacetate (b) silica supported iron trifluoroacetate (c) bulk iron trichloroacetate (d) silica supported iron trichloroacetate and HR-TEM images of (e) bulk iron trifluoroacetate (f) silica supported iron trifluoroacetate (g) bulk iron trichloroacetate (h) silica supported iron trichloroacetate (i) bulk silica gel or silica K100 material.

FE-SEM and HR-TEM were employed to analyse the surface texture and morphology of prepared Lewis acid catalysts. The FE-SEM images of bulk unsupported iron trifluoroacetate and trichloroacetate catalyst samples are depicted in Fig. 3a and c, having soft, irregularly shaped particles with smooth surfaces. While, Fig. 3b and d, respectively depict the FE-SEM images of the iron trifluoroacetate and trichloroacetate supported on silica. The shape and morphology obtained in images of supported Lewis catalysts are substantially comparable to that of the bulk unsupported iron trifluoroacetate and trichloroacetate samples. These intact surface morphology of silica-supported Lewis acids authenticate the thorough dispersion of iron trifluoroacetate and trichloroacetate functionalities in the pores of the mesoporous silica material. Furthermore, in both supported iron trifluoroacetate and trichloroacetate catalysts, no distinct crystallites of the bulk iron trifluoroacetate and trichloroacetate samples were seen.

The high-resolution TEM images of bulk unsupported iron trifluoroacetate and trichloroacetate Lewis acids (Fig. 3e and g) reveal that the majority of the specifically organized fine particles are covered in dark colour. However, the high-resolution TEM images of the iron trifluoroacetate and trichloroacetate supported over silica (Fig. 3f and h), show perfectly layered dark colour fine particles on another surface layer of supporting material. Moreover, HR-TEM image of bulk silica gel or silica K100 material (Fig. 3i) show dark black colour layer of silica material which also appeared in images of the iron trifluoroacetate and trichloroacetate supported on silica. The results of this provide convincing proof that the iron trifluoroacetate and trichloroacetate are evenly distributed throughout the mesoporous pores of the silica support material.

**Fig. 4:**
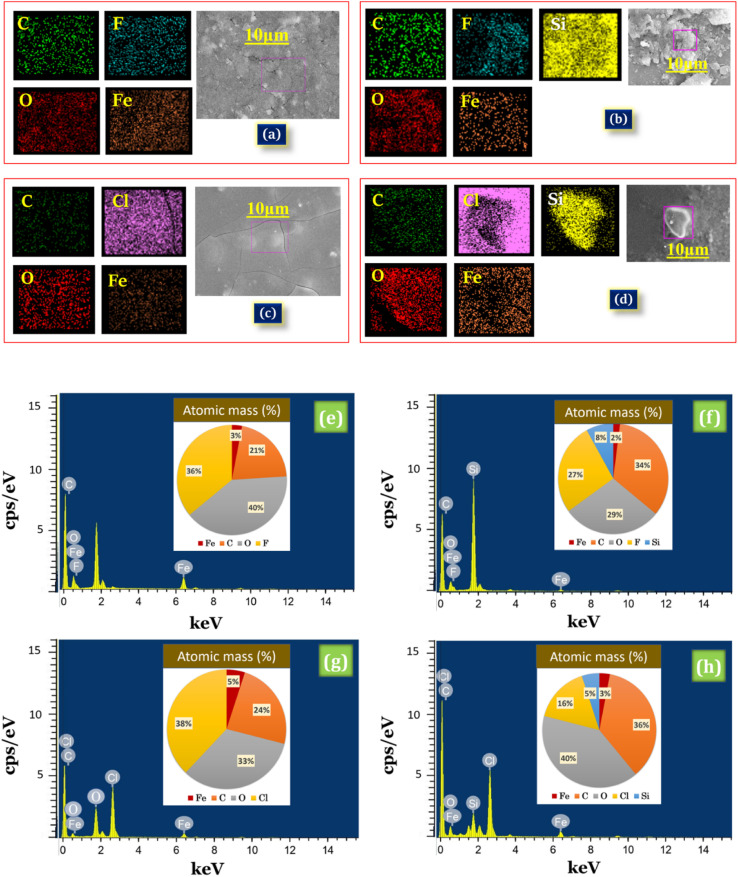
Elemental mapping images of (a) Fe(OCOCF_3_)_3_·*n*H_2_O (b) Fe(OCOCF_3_)_3_·*n*H_2_O/SiO_2_ (c) Fe(OCOCCl_3_)_3_·*n*H_2_O (d) Fe(OCOCCl_3_)_3_·*n*H_2_O/SiO_2_ and EDX spectra for atomic percentage of (e) Fe(OCOCF_3_)_3_·*n*H_2_O (f) Fe(OCOCF_3_)_3_·*n*H_2_O/SiO_2_ (g) Fe(OCOCCl_3_)_3_·*n*H_2_O (h) Fe(OCOCCl_3_)_3_·*n*H_2_O/SiO_2_.

EDX spectral analysis is used to confirm the chemical composition of iron trifluoroacetate, iron trichloroacetate, silica-supported iron trifluoroacetate and silica-supported iron trichloroacetate Lewis acid (Fig. 4). EDX spectra of presently synthesized iron trifluoroacetate [Fe(OCOCF_3_)_3_·*n*H_2_O] confirm the existence of C, O, F, and Fe elements (Fig. 4a), whereas iron trichloroacetate [Fe(OCOCCl_3_)_3_·*n*H_2_O] confirms the occurrence of C, O, Cl, and Fe elements (Fig. 4c). Furthermore, iron trifluoroacetate supported on silica [Fe(OCOCF_3_)_3_·*n*H_2_O/SiO_2_] indicates the occurrence of C, O, F, Fe and Si elements and silica-supported iron trichloroacetate [Fe(OCOCCl_3_)_3_·*n*H_2_O/SiO_2_] show the existence of C, O, Cl, Fe and Si elements (Fig. 4b and d).

Additionally, EDX mapping images depicted in Fig. 4a and c, provide clear validation for a uniform distribution of Fe, O, C and F or Cl elements in the desired iron trifluoroacetate and iron trichloroacetate catalyst system. While, elemental mapping images depicted in Fig. 4b and d clarify the building of a well-dispersed blended material of Fe, O, C, Si, and F or Cl elements in the silica-supported iron trifluoroacetate and iron trichloroacetate Lewis acid catalyst. Afterward, the atomic percentage of Fe, O, C, F, Cl, Si, and O in the synthesized Lewis acid catalysts were determined by the results of the EDX elemental image mapping represented in (Fig. 4e–h). This outcome strongly evidences the formation of silica-supported iron trifluoroacetate and trichloroacetate Lewis acid catalysts which is in moral agreement with spectral results of FTIR, powder XRD, FE-SEM and HR-TEM.

**Fig. 5:**
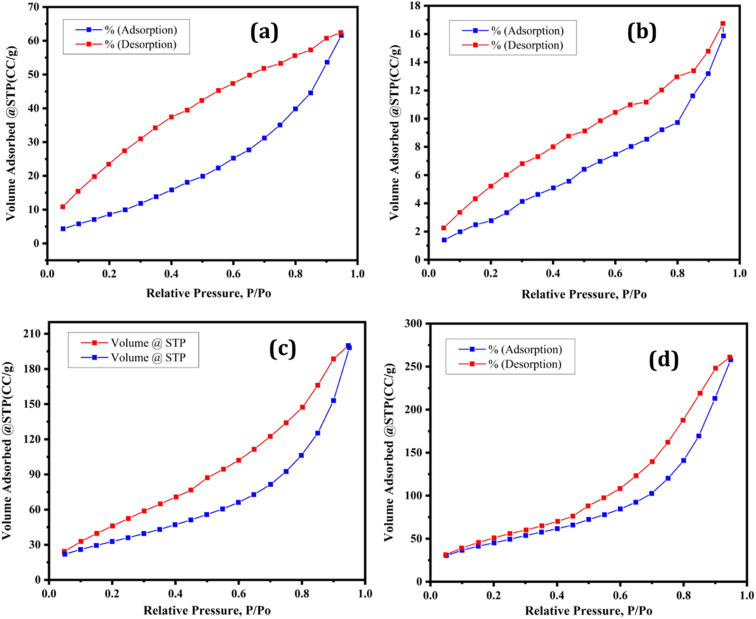
Nitrogen adsorption–desorption isotherms of (a) Fe(OCOCF_3_)_3_·*n*H_2_O (b) Fe(OCOCCl_3_)_3_·*n*H_2_O (c) Fe(OCOCF_3_)_3_·*n*H_2_O/SiO_2_ (d) Fe(OCOCCl_3_)_3_·*n*H_2_O/SiO_2_.

A catalyst's specific surface area is a key predictor. The specific surface area, pore volume, and pore diameter of the synthesized supported and unsupported Lewis acid catalysts were measured using the Brunauer–Emmett–Teller (BET) method. The specific surface area of bulk iron trifluoroacetate (Fe(OCOCF_3_)_3_·*n*H_2_O), iron trichloroacetate (Fe(OCOCCl_3_)_3_·*n*H_2_O), silica supported iron trifluoroacetate (Fe(OCOCF_3_)_3_·*n*H_2_O/SiO_2_) and iron trichloroacetate (Fe(OCOCCl_3_)_3_·*n*H_2_O/SiO_2_) Lewis acid catalysts were 41.617, 28.321, 170.856 and 126.568 m^2^ g^−1^, respectively as depicted in Table 1. The nitrogen adsorption–desorption measurement was used to determine the porosity of these catalyst samples. According to the IUPAC classification, the nitrogen adsorption–desorption isotherm occurs naturally and is of type III.^50^ The bulk iron trifluoroacetate, iron trichloroacetate, silica-supported iron trifluoroacetate and trichloroacetate Lewis acid catalysts all exhibit a well-expressed H_3_ hysteresis loop at high relative pressure (Fig. 5a–d), which is typical for mesoporous materials and ranges from 0.4–1.0 *P*/*P*^0^. The average pore diameters of bulk iron trifluoroacetate, iron trichloroacetate, silica-supported iron trifluoroacetate and iron trichloroacetate catalysts are 9.1564, 6.8528, 9.3339, and 9.6850 nm, respectively, as mentioned in Table 1. Additionally, the present catalyst's total pore volume (0.0953–0.3989 cm^3^ g^−1^) was determined using the BJH method as represented in Fig. 5a–d. Moreover, pure silica gel or silica K100 mesoporous material has a specific surface area 395 m^2^ g^−1^, average pore diameter 9.4 nm, total pore volume 0.93 cm^3^ g^−1^ and type IV isotherms with a hysteresis loop at high relative pressure.^51–53^ The comparative study of BJH plot of pure silica material and silica supported iron trifluoroacetate and trichloroacetate is overserved, which clearly shows alternation in original isotherm and hysteresis of pure silica. This modification is due to strong interaction between mesoporous silica material and iron trifluoroacetate or trichloroacetate Lewis acid.

**Table 1:** BET analysis of the Lewis acid catalyst series

Entry	Catalyst sample	*S*_BET_ (m^2^ g^−1^)	*D*_pore_ (nm)	*V*_pore_ (cm^3^ g^−1^)
1	Fe(OCOCF_3_)_3_·*n*H_2_O	41.617	9.1564	0.0953
2	Fe(OCOCCl_3_)_3_·*n*H_2_O	28.321	6.8528	0.0245
3	Fe(OCOCF_3_)_3_·*n*H_2_O/SiO_2_	170.856	9.3339	0.3989
4	Fe(OCOCCl_3_)_3_·*n*H_2_O/SiO_2_	126.568	9.6850	0.3065

The BET analysis demonstrated that the silica-supported iron trifluoroacetate and trichloroacetate catalysts had a marginally large surface area than their unsupported bulk catalyst. The improved surface area, average pore diameter and total pore volume of supported Lewis acid catalysts can become one of the prominent feature in enhancing catalytic efficiency. The fundamental reason for this may be the deposition and integration of iron trifluoroacetate or trichloroacetate inside the pores of the mesoporous silica support material.

**Fig. 6:**
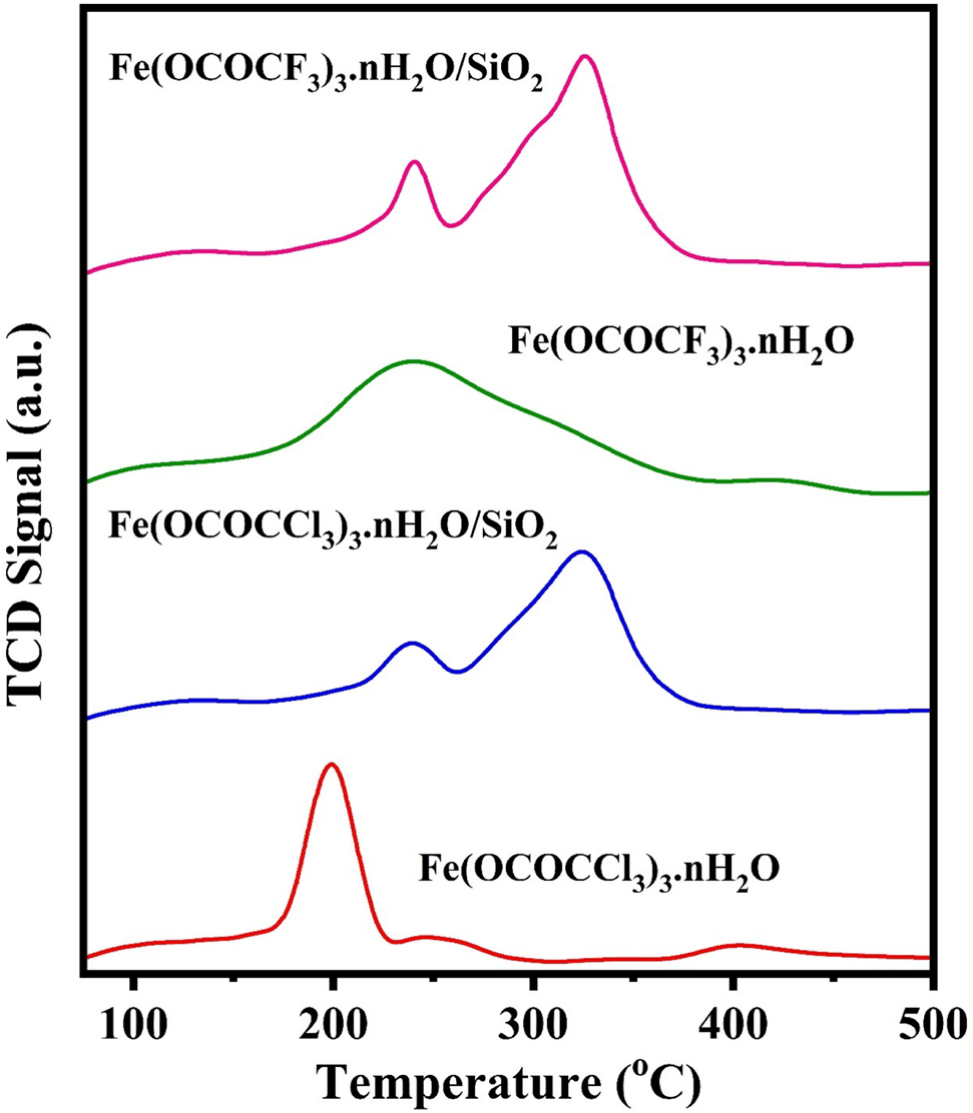
Temperature-programmed desorption of ammonia (NH_3_-TPD) profiles of bulk iron trifluoroacetate (Fe(OCOCF_3_)_3_·*n*H_2_O), iron trichloroacetate (Fe(OCOCCl_3_)_3_·*n*H_2_O), silica supported iron trifluoroacetate (Fe(OCOCF_3_)_3_·*n*H_2_O/SiO_2_) and iron trichloroacetate (Fe(OCOCCl_3_)_3_·*n*H_2_O/SiO_2_) Lewis acid catalysts.

Acid strength and acid quantity of synthesized catalyst samples were determined using NH_3_-TPD characterization. The ammonia desorption peak temperature determines the acid concentration, whilst the areas under the TPD curves provide an estimate of the acid quantity. The NH_3_-TPD profiles of four Lewis acid catalyst samples are presented in Fig. 6 and Table 2 listed their relative quantities and the acidity of the acidic sites. As depicted in Fig. 6, bulk iron trifluoroacetate (Fe(OCOCF_3_)_3_·*n*H_2_O) and iron trichloroacetate (Fe(OCOCCl_3_)_3_·*n*H_2_O) shows single NH_3_ desorption peaks respectively at 234 °C and 204 °C corresponding to the weak acidic sites. However, silica-supported iron trifluoroacetate (Fe(OCOCF_3_)_3_·*n*H_2_O/SiO_2_) and iron trichloroacetate (Fe(OCOCCl_3_)_3_·*n*H_2_O/SiO_2_) Lewis acid shows two NH_3_ desorption peaks which corresponding to the weak acidic site and strong acidic site. In these, peaks at 242 °C and 236 °C correspond to the weak acidic sites, while peaks at 328 °C and 322 °C corresponds to the strong acidic sites of respective Lewis acid catalysts.^12,54^

**Table 2:** Acid strength and acid quantity of different Lewis acid catalysts

Catalyst	Weak acid sites		Strong acid sites		Total acidity (mmol g^−1^)
	Peak temp. (°C)	Acid amount (mmol g^−1^)	Peak temp. (°C)	Acid amount (mmol g^−1^)	
Fe(OCOCF_3_)_3_·*n*H_2_O	234 °C	0.038	—	—	0.038
Fe(OCOCCl_3_)_3_·*n*H_2_O	204 °C	0.025	—	—	0.025
Fe(OCOCF_3_)_3_·*n*H_2_O/SiO_2_	242 °C	0.024	328 °C	0.075	0.099
Fe(OCOCCl_3_)_3_·*n*H_2_O/SiO_2_	236 °C	0.020	322 °C	0.071	0.091

Here, Fig. 6 indicate that total acidity, acidic site and acid strength were enhanced in supported Lewis acid as compared to bulk unsupported Lewis acid, which is summarized in Table 2. This is due to the fine distribution and interaction of iron trifluoroacetate/trichloroacetate with mesoporous silica material. Moreover, this strong interaction in silica and Lewis acid also increase the thermal stability of the catalyst and hence results into the change in NH_3_ desorption peaks to the higher temperature. Further, in supported Lewis acid catalyst these increase in total acidity, acidic site and acidic strength become a key feature for enhancement of catalytic efficiency.

**Fig. 7:**
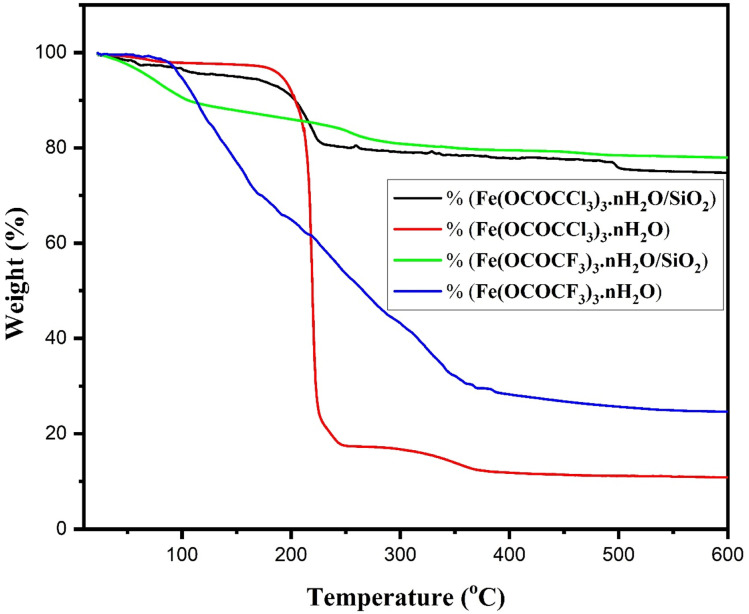
TGA of iron trifluoroacetate, silica supported iron trifluoroacetate, iron trichloroacetate and silica supported iron trichloroacetate catalysts.

Thermal stability of currently synthesized Lewis acid catalysts was studied by thermogravimetric analysis (Fig. 7). All the Lewis acid catalysts have their first distinctive TG curves, at the temperature range of 86 °C to 168 °C related to the loss of water molecules. The second characteristic TG curve at the temperature range of 178 °C to 246 °C with weight loss of 46% and 71% in unsupported iron trifluoroacetate and trichloroacetate catalysts respectively. This weight loss was assumed to be caused by a partial loss of the trifluoroacetate and trichloroacetate group of anhydrous Lewis acid. While third TG curve at the temperature range of 247 °C to 370 °C was corresponding to the complete loss of trifluoroacetate and trichloroacetate groups leading to the formation of FeF_3_ and FeCl_3_ as the decomposition product with a high weight loss of 76% and 90% respectively.^14,44,45^ However, the second characteristic TG curve for silica supported iron trifluoroacetate and trichloroacetate catalysts, caused due to the partial loss of trifluoroacetate and trichloroacetate groups are obtained at the temperature range of 210 °C to 308 °C with minimum weight loss of 17 and 20%, respectively. Further, the third TG curve was observed at the temperature range of 367 °C to 428 °C, corresponding to the complete loss of trifluoroacetate and trichloroacetate groups with maximum weight loss of 21 and 23% respectively.

Hence, the comparative thermogravimetric analysis of unsupported iron trifluoroacetate, iron trichloroacetate, silica supported iron trifluoroacetate and trichloroacetate Lewis acid catalysts provides clear evidence for higher thermal stability of silica supported Lewis acid catalysts than that of the unsupported Lewis acid alone. This must be caused by the results of the strong interaction of bulk iron trifluoroacetate or trichloroacetate with silica material. This improvement in thermal stability of the supported Lewis acid catalysts enables them to operate throughout a moderate temperature range without suffering a major reduction in catalytic activity, allows the reaction to occur at higher temperatures and avoids metal sintering to extend the catalyst's lifespan.

### 3.2 Catalytic evaluation of silica supported Lewis acid catalysts for the synthesis of hexahydroquinoline-3-carboxamides derivatives

**Scheme 3:**
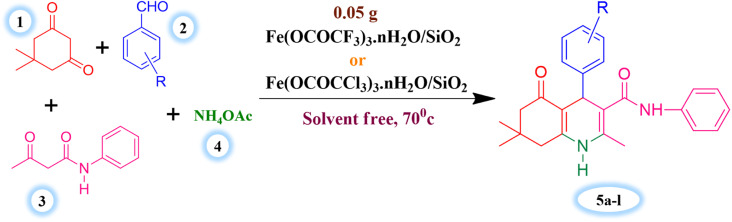
Silica supported iron trifluoroacetate and trichloroacetate Lewis acid catalysed synthesis of hexahydroquinoline-3-carboxamides.

The major objective of present research work was to evaluate the effectiveness of synthesized Lewis acid catalysts and optimize the most favourable and efficient reaction conditions for the one-pot four component synthesis of hexahydroquinoline-3-carboxamides derivatives. Benzaldehyde (1 mmol), dimedone (1 mmol), acetoacetanilide (1 mmol) and ammonium acetate (1.2 mmol) were taken as substrates for the model reaction of current synthetic protocol (Scheme 3).

Initially, the model reaction was carried in presence of synthesized unsupported Fe(OCOCF_3_)_3_·*n*H_2_O & Fe(OCOCCl_3_)_3_·*n*H_2_O Lewis acid catalysts and silica supported Fe(OCOCF_3_)_3_·*n*H_2_O/SiO_2_ & Fe(OCOCCl_3_)_3_·*n*H_2_O/SiO_2_ Lewis acid catalysts, under solvent free condition to assess the effectiveness of these catalysts (Table 3). The supported Lewis acid catalysts show higher catalytic activity in terms of reaction time and product yield as compared to similar catalysts without silica support. It was noticed that product yield and reaction speed was modified a lot by the silica supported Lewis acid catalysts. However, pure silica supporting material exhibits exceedingly low catalytic activity in respect of product yield and reaction time.

**Table 3:** Effect of current series of green Lewis acid catalyst in the hexahydroquinoline-3-carboxamide synthesis (5a)^a^

Entry	Catalyst series	Amount of catalyst	Reaction time^b^ (min)	Product yield^c^ (%)
1	Kieselgel K100 (silica gel)	50 mg	300	34
2	Fe(OCOCCl_3_)_3_·*n*H_2_O	50 mg	30	79
3	Fe(OCOCF_3_)_3_·*n*H_2_O	50 mg	27	82
4	**Fe(OCOCCl**_**3**_ **)**_**3**_ **·*n*H**_**2**_ **O/SiO**_**2**_	50 mg	**09**	**98**
5	**Fe(OCOCF**_**3**_ **)**_**3**_ **·*n*H**_**2**_ **O/SiO**_**2**_	50 mg	**08**	**98**

a Reaction conditions: benzaldehyde (1 mmol), dimedone (1 mmol), acetoacetanilide (1 mmol), ammonium acetate (1.2 mmol) and green Lewis acid catalyst.

b Reaction progress monitored by TLC.

c Isolated yields.

These results (Table 3) clearly indicate that the Lewis acid catalyst supported on silica exhibits maximum catalytic efficiency. However, the well-dispersed Lewis acid catalyst made of iron trifluoroacetate or trichloroacetate on the silica supporting material was the cause of increased catalytic activity for a supported Lewis acid catalyst. This means that it would have more surface area and functional Lewis and Brønsted sites than a bulk catalyst made of iron trifluoroacetate or trichloroacetate.

Further, to identify the ideal reaction conditions for current synthetic protocol using silica supported iron trifluoroacetate or trichloroacetate Lewis acid catalysts, we have also monitored the model reaction under a variety of conditions, including the different solvents, temperature, and catalyst quantity. To determine the suitable reaction medium for the current synthetic protocol in presence of synthesized Lewis acid catalysts, a variety of solvents (polar and non-polar solvents) were used. The best outcomes in terms of reaction time and product yield were found to be produced in a solvent-free environment (Table 4). This must be due to the fact that the catalyst's porous structure, which is more effective in solvent-free circumstances due to the increased availability of reactant molecules, provides for simple access to the active sites through its pores. In a solvent, the presence of solvent molecules decreases the availability of reactant molecules, lowering the yield of product compared to a solvent-free reaction.

**Table 4:** Optimization of solvents in the hexahydroquinoline-3-carboxamide synthesis in presence of silica-supported green Lewis acid catalyst (5a)^a^

Entry	Solvents	Reaction condition	Time^b^ (min)		Product yield^c^ (%)	
			I catalyst	II catalyst	I catalyst	II catalyst
1	Water	Reflux	58	60	62	58
2	Ethanol	Reflux	21	24	92	89
3	DMF	Reflux	38	41	71	67
4	THF	Reflux	34	39	82	74
5	Acetonitrile	Reflux	43	45	74	71
6	Chloroform	Reflux	40	41	67	63
7	Toluene	Reflux	55	58	64	60
**8**	**Solvent free**	**70 °C**	**08**	**09**	**98**	**98**

a Reaction conditions: benzaldehyde (1 mmol), dimedone (1 mmol), acetoacetanilide (1 mmol), ammonium acetate (1.2 mmol) and silica supported iron trifluoroacetate and trichloroacetate Lewis acid catalysts. (I catalyst) – silica supported iron trifluoroacetate. (II catalyst) – silica supported iron trichloroacetate.

b Reaction progress monitored by TLC.

c Isolated yields.

Moreover, the optimization of reaction temperature was carried under solvent free conditions in the synthesis of hexahydroquinoline-3-carboxamide mentioned in Table 5. As the temperature enhances from 50 °C to 70 °C, the reaction time and product yield improved gradually and steadily. As a result of the findings, 70 °C has been determined to be the optimal reaction temperature for this one pot, solvent-free synthesis of hexahydroquinoline-3-carboxamides. Additionally, an increase in temperature above 70 °C had no discernible effects on the reaction's progress time or product yield.

**Table 5:** Optimization of the reaction temperature in hexahydroquinoline-3-carboxamide synthesis (5a)^a^

Entry	Reaction medium	Reaction temperature	Time^b^ (min)		Product yield^c^ (%)	
			I catalyst	II catalyst	I catalyst	II catalyst
1	Solvent free	50 °C	18	20	75	72
2	Solvent free	60 °C	12	13	88	86
**3**	**Solvent free**	**70 °C**	**08**	**09**	**98**	**98**
4	Solvent free	80 °C	08	09	98	98
5	Solvent free	90 °C	08	09	98	98

a Reaction conditions: benzaldehyde (1 mmol), dimedone (1 mmol), acetoacetanilide (1 mmol), ammonium acetate (1.2 mmol) and silica supported iron trifluoroacetate and trichloroacetate Lewis acid catalysts. (I catalyst) – silica supported iron trifluoroacetate. (II catalyst) – silica supported iron trichloroacetate.

b Reaction progress monitored by TLC.

c Isolated yields.

**Fig. 8:**
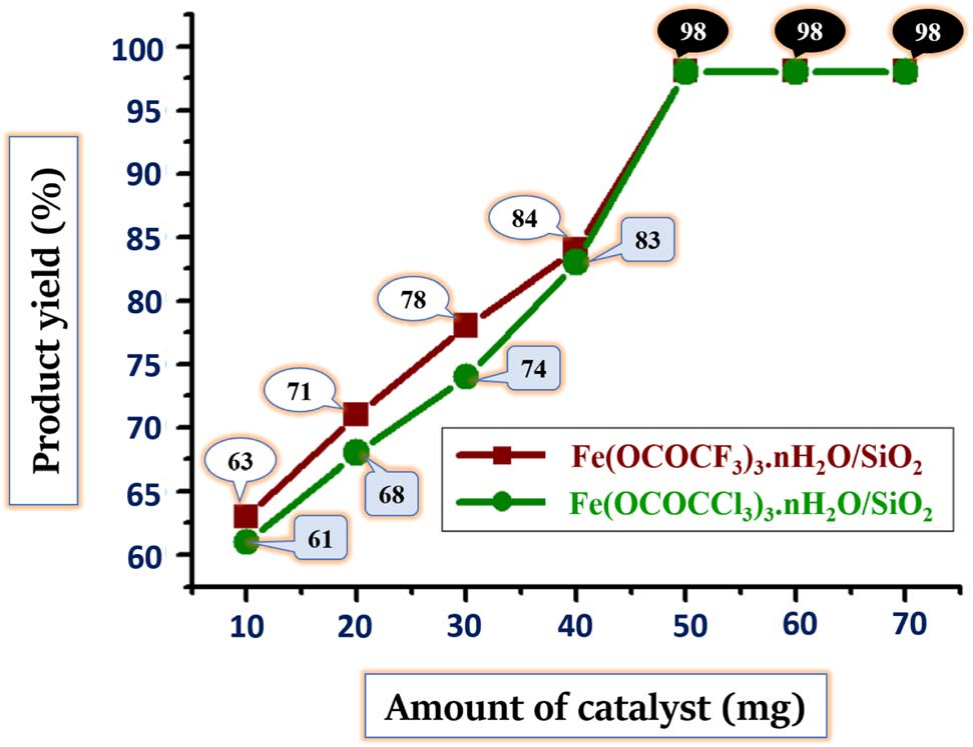
Optimization of the stoichiometric amount of silica supported Lewis acid catalysts and comparison of product yield (%) for the synthesis of (5a) *via* solvent-free condition.

Another important parameter in assessment of current synthetic protocol is the determination of the proper and stoichiometric amount of the catalyst. To find out the proper amount of catalyst needed in reaction, the model reaction was run in a solvent-free environment with various concentrations (10, 20, 30, 40, 50, 60, and 70 mg) of both silica-supported iron trifluoroacetate and trichloroacetate Lewis acid catalysts. The results are summarised in Table 6 and Fig. 8.

**Table 6:** Optimization of the amount of silica supported Lewis acid catalysts and comparison of reaction time and product yield (%) for the synthesis of (5a)^a^*via* solvent-free condition

Entry	Amount of catalyst (mg)	Reaction temperature	Time^b^ (min)		Product yield^c^ (%)	
			I catalyst	II catalyst	I catalyst	II catalyst
1	10	70 °C	08	09	63	61
2	20	70 °C	08	09	71	68
3	30	70 °C	08	09	78	74
4	40	70 °C	08	09	84	83
**5**	**50**	**70 °C**	**08**	**09**	**98**	**98**
6	60	70 °C	08	09	98	98
7	70	70 °C	08	09	98	98

a Reaction conditions: benzaldehyde (1 mmol), dimedone (1 mmol), acetoacetanilide (1 mmol), ammonium acetate (1.2 mmol) and silica supported iron trifluoroacetate and trichloroacetate Lewis acid catalyst. (I catalyst) – silica supported iron trifluoroacetate. (II catalyst) – silica supported iron trichloroacetate.

b Reaction progress monitored by TLC.

c Isolated yields.

As the amount of silica supported Lewis acid catalysts rises gradually, product yield in reaction increases (Table 6, entries 1–7 and Fig. 8). The outcomes show that, at a temperature of 70 °C, 50 mg or 0.05 g of both the silica-supported iron trifluoroacetate and trichloroacetate Lewis acid catalysts gave highest product yield of 98%. The product yields remain unchanged when the quantity of these Lewis acid catalysts increased further by 60 and 70 mg (Table 6, entries 6, 7 and Fig. 8). This can be due to the reason that catalysts may have reached their maximum conversion efficiency.

Finally, hexahydroquinoline-3-carboxamide derivatives (5a–l) were synthesized using the present catalytic system under the ideal reaction circumstances. Herein, excellent outcomes were obtained and a detailed description of these was provided in Table 7. Moreover, silica supported iron trifluoroacetate exhibits higher catalytic activity than silica supported iron trichloroacetate with respect to all optimization parameters involved in synthesis of the hexahydroquinoline-3-carboxamide derivatives (5a–l). This is actually because high electronegative fluorine atoms have a more withdrawing character than chlorine atoms, which increases the acidity of Lewis acid catalyst.

**Table 7:** Silica supported iron trifluoroacetate (Fe(OCOCF_3_)_3_·*n*H_2_O/SiO_2_) and iron trichloroacetate (Fe(OCOCCl_3_)_3_·*n*H_2_O/SiO_2_) Lewis acid prompted synthesis of hexahydroquinoline-3-carboxamides derivatives (5a–l)^a^

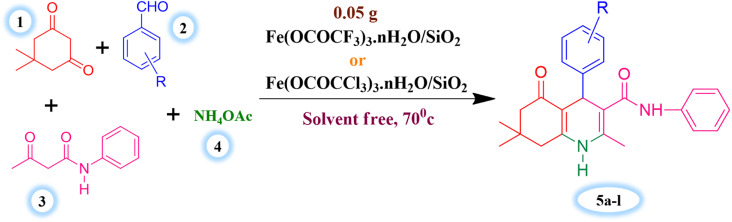							
Entry	Substituent ‘R’	Time^b^ (min)		Yield^c^ (%)		Mp (°C) observed	Mp (°C) reported
		I catalyst	II catalyst	I catalyst	II catalyst		
5a	H	08	09	98	98	245–247	242–244 (ref. 55)
5b	4-OMe	08	09	98	96	246–248	247–249 (ref. 56)
5c	4-Me	08	10	96	95	252–255	251–253 (ref. 57)
5d	4-Br	10	11	94	91	228–230	232–233 (ref. 57)
5e	4-F	10	11	94	92	205–207	200–202 (ref. 58)
5f	4-Cl	10	11	95	91	255–257	251–253 (ref. 55)
5g	4-NO_2_	12	15	92	90	207–210	209–211 (ref. 59)
5h	4-OH	09	11	96	94	>300	>300 (ref. 59)
5i	2-Cl	10	11	94	93	228–230	225–227 (ref. 60)
5j	2-Br	10	12	93	91	241–242	241–243 (ref. 57)
5k	3-Br	09	11	92	91	209–210	211–213 (ref. 60)
5l	3-NO_2_	13	15	91	88	248–251	244–246 (ref. 59)

a Reaction conditions: benzaldehyde (1 mmol), dimedone (1 mmol), acetoacetanilide (1 mmol), ammonium acetate (1.2 mmol) and (I catalyst) – silica supported iron trifluoroacetate or (II catalyst) – silica supported iron trichloroacetate.

b Reaction progress monitored by TLC.

c Isolated yields.

The comparison of catalytic efficiency of silica supported iron trifluoroacetate and trichloroacetate Lewis acid was performed with other catalysts reported in the literature, for the synthesis of hexahydroquinoline-3-carboxamides derivatives is depicted (Table 8). In comparison of other literature reported catalysts, the silica supported iron trifluoroacetate and trichloroacetate Lewis acid catalysts work superiorly in terms of amount of catalyst, reaction times and product yield. Henceforth, the current Lewis acid promoted synthesis of hexahydroquinoline-3-carboxamides become one of the excellent alternatives to these catalysts. This makes the current hexahydroquinoline-3-carboxamides synthetic route more economically convenient, and environment friendly.

**Table 8:** Comparative study of the current Lewis acid catalysts with literature known catalysts utilized in synthesis of hexahydroquinoline-3-carboxamides derivatives (5a)^a^

Entry	Catalyst	Conditions	Time^b^ (min)	Yield^c^ (%)
1	—	Water, reflux	720	—
2	Yb(OTf)_3_	CH_3_CN, RT	300	82 (ref. 61)
3	Sc(OTf)_3_	EtOH, RT	240	93 (ref. 62)
4	Fe_3_O_4_@CS@Ag@CH_2_COOH	EtOH/70 °C	60	95 (ref. 55)
5	FePO_4_	EtOH/reflux	60	94 (ref. 63)
6	[CPySO_3_H]^+^Cl^−^	EtOH/70 °C	45	95 (ref. 60)
7	Verjuice	EtOH/70 °C	20	95 (ref. 59)
8	[2-MPy][p-TSA]	EtOH/50 °C	20	94 (ref. 56)
9	PTSA	Grinding/EtOH	15	78 (ref. 64)
10	Nano-γ-Fe_2_O_3_–SO_3_H	Solvent-free/70 °C	65	90 (ref. 65)
11	LAIL@NMP	Solvent-free/80 °C	65	97 (ref. 66)
12	MCM-41	Solvent-free/90 °C	25	88 (ref. 67)
13	Fe(OCOCCl_3_)_3_·*n*H_2_O/SiO_2_	Solvent-free, 70 °C	09	98 (this work)
14	Fe(OCOCF_3_)_3_·*n*H_2_O/SiO_2_	Solvent-free, 70 °C	08	98 (this work)

a Reaction conditions: benzaldehyde (1 mmol), dimedone (1 mmol), acetoacetanilide (1 mmol), ammonium acetate (1.2 mmol) and silica supported iron trifluoroacetate and trichloroacetate Lewis acid catalysts.

b Reaction progress monitored by TLC.

c Isolated yields.

**Fig. 9:**
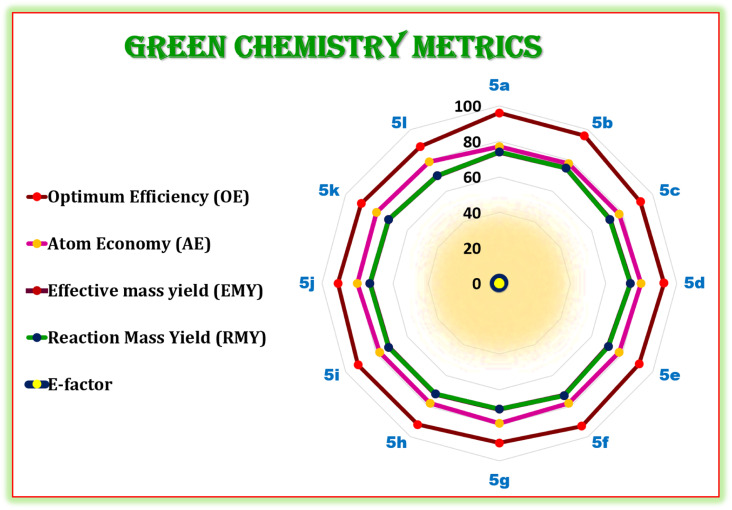
Radar chart of the measured green metrics for synthesis of hexahydroquinoline-3-carboxamides (5a–l).

Further, in term of sustainability, it is necessary to validate the current synthetic protocol using well-recognized ‘green chemistry metrics’ including the E-factor, atom economy, reaction mass efficiency, optimum efficiency, and reaction mass yield.^68,69^ These measures are used to measure a chemical reaction's effectiveness or environmental performance.^70^ Moreover, the E-factor is widely utilized in green chemistry metrics of chemical reactions. The reaction is more environmentally friendly and eco-compatible when the E-factor value is lower.^71^ The current synthetic protocol is green, as evidenced by the E-factor, which ranges from 0.33 to 0.42 in (Fig. 9).

Later on, we have calculated and demonstrated the green chemistry metrics for the hexahydroquinoline-3-carboxamides (5a–l). The results showed that values of green chemistry metrics including atom economy (AE), E-factor, optimum efficiency (OE), reaction mass efficiency (RME), and effective mass yield (EMY) are close to their ideal values as shown (see the ESI for detailed calculations†).

### 3.3 Proposed active center or site of silica supported iron trifluoroacetate and trichloroacetate Lewis acid catalyst

**Fig. 10:**
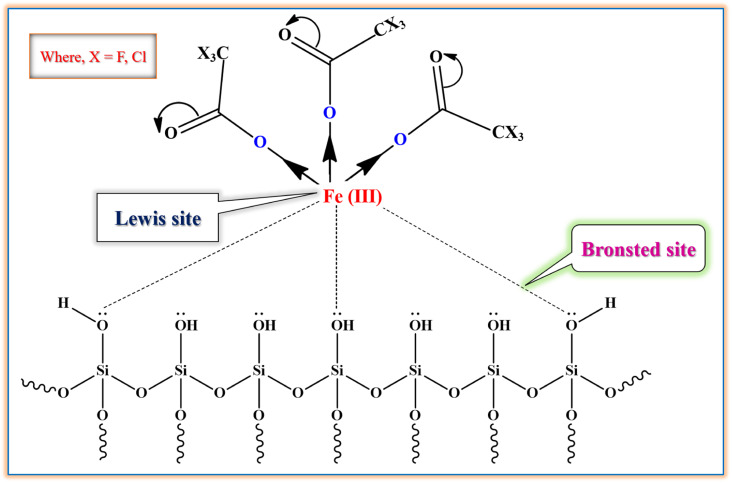
The proposed active sites of silica supported iron trifluoroacetate and trichloroacetate Lewis acid.

The active sites in silica supported iron trifluoroacetate and trichloroacetate Lewis acid catalysts is fully defined in Fig. 10. The current Lewis acid catalysts especially point out Lewis acidic center or site on iron metal surface which is interacted with mesoporous silica surface. The iron metal (Fe) loses electron density when exposed to the trifluoroacetate or trichloroacetate group, becoming electron deficient and exhibiting Lewis acidity. The interaction of surface –OH groups of silica with trifluoroacetate or trichloroacetate groups binds them to the silica surface without changing its Lewis acidic character. Moreover, it is widely known that the iron trifluoroacetate and trichloroacetate catalysts supported by silica primarily include Lewis acidity, which actually originated from the loaded iron trifluoroacetate or trichloroacetate.^14,72^

### 3.4 Recycling of catalyst

**Fig. 11:**
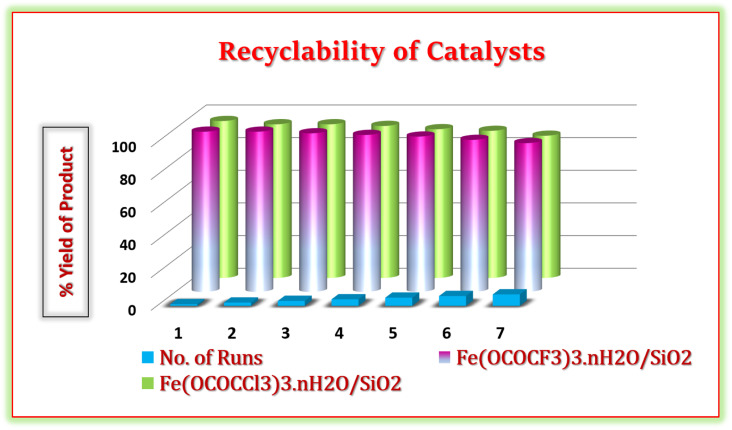
Reusability of silica supported iron trifluoroacetate and trichloroacetate catalyst in the synthesis of hexahydroquinoline-3-carboxamide derivatives.

**Fig. 12:**
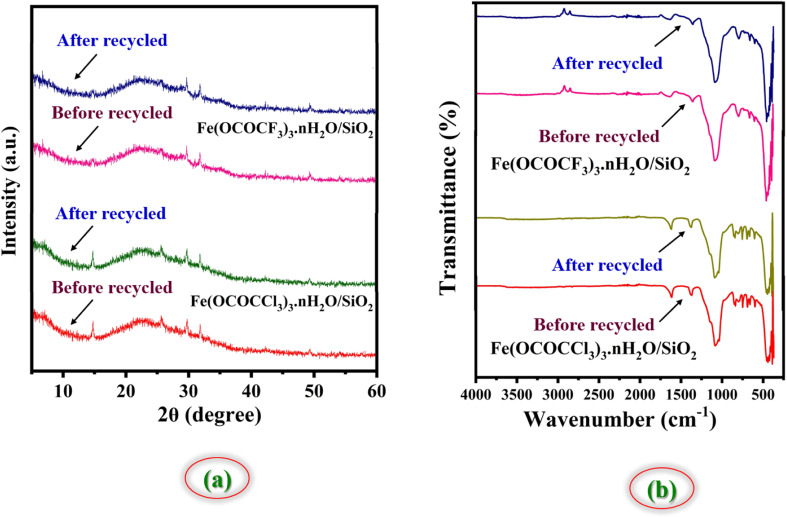
(a) XRD spectrum of silica-supported iron trifluoroacetate and iron trichloroacetate Lewis acid before recycled and after recycled. (b) FTIR spectrum of silica-supported iron trifluoroacetate and iron trichloroacetate Lewis acid before recycled and after recycled.

One of the main aspects of the present synthetic protocol is that the catalysts can be recycled. The present catalysts were regenerated from the reaction mixture using a simple filtration method. At end of the reaction, the reaction mixture was diluted with hot ethanol and filtered for the separation of the catalyst. Further, filtered solid catalysts were washed by 1 : 1 ethanol–water system and kept for drying and activation in vacuum-oven for 20 min at 80 °C. Afterward, they were weighed and used for the next run. They were employed seven times in the model reaction without any additional treatment in reaction (Fig. 11). It was observed that regenerated catalysts almost have consistent catalytic activity. These studies demonstrate that current Lewis acids are the most efficient and reusable catalyst without suffering a significant reduction in catalytic activity. These recycled catalysts were recovered and identified *via* FTIR and PXRD analysis after seven cycles. The X-ray diffraction peaks of recovered catalysts at 2*θ* degree value = 14, 29, 31, and 49 were found to be exactly identical to the fresh silica-supported iron trifluoroacetate and trichloroacetate Lewis acid catalysts, depicted in Fig. 12a. Moreover, FTIR spectrum also shows characteristics vibrational bands at 1631, 1622, 1359, 1377, 1155, 1125, 1205, 1080 and 684 cm^−1^ of recovered catalysts which were similar to the fresh silica supported iron trifluoroacetate and trichloroacetate Lewis acids, depicted in Fig. 12b.

### 3.5 Plausible reaction mechanism

**Scheme 4:**
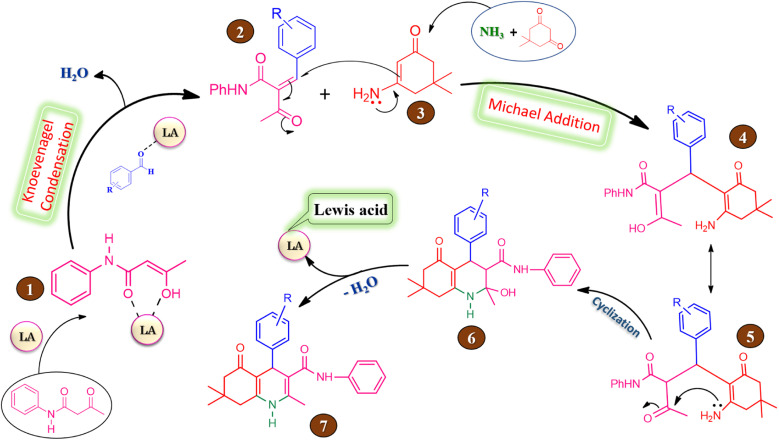
The proposed reaction mechanism for the synthesis of hexahydroquinoline-3-carboxamides derivatives in presence of silica supported iron trifluoroacetate or trichloroacetate Lewis acid catalyst.

The probable reaction mechanism for the four component one-pot synthesis of hexahydroquinoline-3-carboxamide mediated by silica supported iron trifluoroacetate or trichloroacetate Lewis acid catalyst is represented in Scheme 4. The overall reaction proceeds *via* Knoevenagel condensation followed by Michael addition.^58,73^

The keto form of acetoacetanilide converts into its enol form in the presence of silica supported iron trifluoroacetate or trichloroacetate Lewis acid catalyst which leads to increase in the nucleophilicity of active methylene carbon of acetoacetanilide. In first step, the enol form of acetoacetanilide (1) attacks on carbonyl carbon of aromatic aldehyde leads to formation of 2-benzylidene-3-oxo-*N*-phenyl butanamide intermediate (2) *via* Knoevenagel condensation reaction. In second step, Michael addition takes place between intermediate (2) and 3-amino-5,5-dimethylcyclohex-2-enone (3) leads to the generation of intermediate (4), which on tautomerization gives intermediate (5). Further, an intramolecular cyclization occurs by the nucleophilic attack of amino group on the carbonyl group of intermediate (5) leads to the formation of intermediate (6), which undergoes dehydration to generate hexahydroquinoline-3-carboxamide (7). Moreover, the catalyst is regenerated at the end of each reaction are freely available for the next reaction cycle.

## 4. Conclusion

Herein, silica supported iron trifluoroacetate and trichloroacetate were developed as recyclable and water-competent green Lewis acid catalysts by a novel and environment-friendly approach. The efficiency of current Lewis acid catalysts were assessed in the hexahydroquinoline-3-carboxamides (5a–l) synthesis. These catalysts work prominently without loosening their catalytic activity in water and organic solvents, as compared to conventional Lewis acid catalysts. In the present synthetic protocol, excellent reaction conditions were obtained in solvent-free reaction conditions at 70 °C temperature. This synthetic protocol has many interesting merits as being the most efficient and favourable route with superb product yield in a short reaction time and easy work-up procedure.

Moreover, comparative study of silica supported iron trifluoroacetate and trichloroacetate catalysts was also carried out in respect of reaction time, reaction temperature, and different reaction conditions which effects on product yield. The results achieved from this clearly indicate that silica supported iron trifluoroacetate has more catalytic activity and efficiency than silica supported iron trichloroacetate. The present Lewis acid catalysts offer many remarkable features such as non-hazardous, water compatible, reusable in reactions and worked effectively in various organic solvents with excellent product yield. Further, they are also non-hygroscopic, moisture insensitive, and made from readily available cheaper starting materials and less expensive than metal triflate and other Lewis acid catalysts. As a result, they may become one of the excellent competent alternatives to both metal triflate Lewis acid and heterogeneous acid catalysts.

## Conflicts of interest

The authors declare no conflict of interest is involved in publication of this research paper.

## Supplementary Material

RA-013-D3RA03542E-s001
