# Supplementary material for: Water compatible silica supported iron trifluoroacetate and trichloroacetate: as prominent and recyclable Lewis acid catalysts for solvent-free green synthesis of hexahydroquinoline-3-carboxamides
Source: RSC Adv. 2023 Aug 4;13(34):23431–48. doi: 10.1039/d3ra03542e (PMC10401520; doi:10.1039/d3ra03542e)
Supplement: RA-013-D3RA03542E-s001 [file RA-013-D3RA03542E-s001.pdf]

Electronic Supplementary Material (ESI) for RSC Advances.
This journal is © The Royal Society of Chemistry 2023

Water compatible silica supported Iron trifluoroacetate and trichloroacetate: As the prominent and recyclable Lewis acid catalysts for solvent-free green synthesis of hexahydroquinoline-3-carboxamides

Dnyaneshwar Purushottam Gholap[1], Ramdas Huse[1], Sudarshan Dipake[1] and Dr. M. K. Lande[1*,]

[1]Department of Chemistry, Dr. Babasaheb Ambedkar Marathwada University, Aurangabad, Maharashtra, India.

*Corresponding Author: Email Id: mkl_chem@yahoo.com

Supporting Information

Content

1. Validation of Green metrics for synthesized organic derivatives 5a-l.

2. ^{1}H, ^{13}C NMR, FTIR, Mass spectra of the organic derivatives.

1. Validation of Green metrics for synthesized organic derivatives (5a-l)

Calculation of green metrics

Table 1: Materials used for green metrics calculation

Reactant 1	Reactant 2	Reactant 3	Reactant 4
Benzaldehyde 0.50gm (Mol. Wt. = 106.12)	Acetoacetanilide 0.83 gm (Mol. Wt. = 177.20).	5, 5-dimethyl-1, 3-cyclohexanedione 0.66 gm (Mol. Wt. = 140.18)	Ammonium acetate 0.43 gm (Mol. Wt. = 77.08)
4-methoxybenzaldehyde 0.64gm (Mol. Wt. = 136.15)			
4-methybenzaldehyde 0.56gm (Mol. Wt. = 120.15)			
4-bromobenzaldehyde 0.87gm (Mol. Wt. = 185.03)			
4-fluoroobenzaldehyde 0.58gm (Mol. Wt. = 124.11)			
4-chlorobenzaldehyde 0.66gm (Mol. Wt. = 140.57)			
4-nitrobenzaldehyde 0.71gm (Mol. Wt. = 151.12)			
4-hydroxybenzaldehyde 0.57gm (Mol. Wt. = 122.12)			
2-chlorobenzaldehyde 0.66gm (Mol. Wt. = 140.57)			
2-bromobenzaldehyde 0.87gm (Mol. Wt. = 185.03)			
3-bromobenzaldehyde 0.87gm (Mol. Wt. = 185.03)			
3-nitrobenzaldehyde 0.71gm (Mol. Wt. = 151.12)			

Analysis of green metrics

The following listed formulae were used for calculating the E-factor, Atom economy (AE), Reaction mass efficiency (RME), Effective mass yield (EMY), and Optimum efficiency (OE). The Calculated data for compounds 5a-l are presented in Table 2. Calculation of green chemistry metrics for one representative entry, viz. 5a

- **E-factor** = Total mass waste (g) / Mass of product

  = Total input (g) – Total output (g) / Total output (g)

  e.g. For the product 5a; E-factor = (0.5 gm + 0.83 gm + 0.66 gm + 0.43 gm) – 1.79 / 1.79

  = 0.35

- **Atom Economy (%)** = (Mol. wt. of product) / (Total mol. wt. of reactants) x 100

  e.g. For the product 5a; AE = (386.49) / (106.12 + 177.20 + 140.18 + 77.08) x 100

  = (386.49) / (500.58) x 100

  = 77 %

- **Reaction mass efficiency (%)** = (Mass of crude product) / (Total mass of reactant) x 100

  e.g. For the product 5a; RME = (1.79) / (0.5 gm + 0.83 gm + 0.66 gm + 0.43 gm) x 100

  = (1.79) / (2.42 gm) x 100

  = 74 %

- **Effective mass yield (%)** = (Mass of Product) / (Mass of non-benign reagents) x 100

  e.g. For the product 5a; EMY = (1.79) / (2.42 gm) x 100

  = 74 %

- **Optimum efficiency (%)** **=** Reaction mass efficiency / Atom economy x 100

  e.g. For the product 5a; OE = (74) / (77) x 100

  = 96 %

Table 2: Green metrics calculation data

Entry	Product	E-factor	Atom Economy (AE)	Reaction Mass Efficiency (RME)	% Effective Mass Yield (EMY)	% Optimum Efficiency (OE)
1.	5a	0.35	77%	74%	74%	96%
2.	5b	0.33	78%	75%	75%	96%
3.	5c	0.38	78%	72%	72%	92%
4.	5d	0.35	80%	74%	74%	93%
5.	5e	0.40	78%	71%	71%	91%
6.	5f	0.37	78%	73%	73%	93%
7.	5g	0.40	79%	71%	71%	90%
8.	5h	0.38	78%	72%	72%	92%
9.	5i	0.39	78%	72%	72%	92%
10.	5j	0.36	80%	73%	73%	91%
11.	5k	0.38	80%	72%	72%	90%
12.	5l	0.42	79%	70%	70%	89%

Note: Since the silica supported Iron trifluoroacetate Lewis acid catalyst was recovered from the reaction protocol, their amount is not included in the calculations of green metrics.

2. ^{1}H, ^{13}C NMR, FTIR, Mass spectra of the organic derivatives (5a-l)

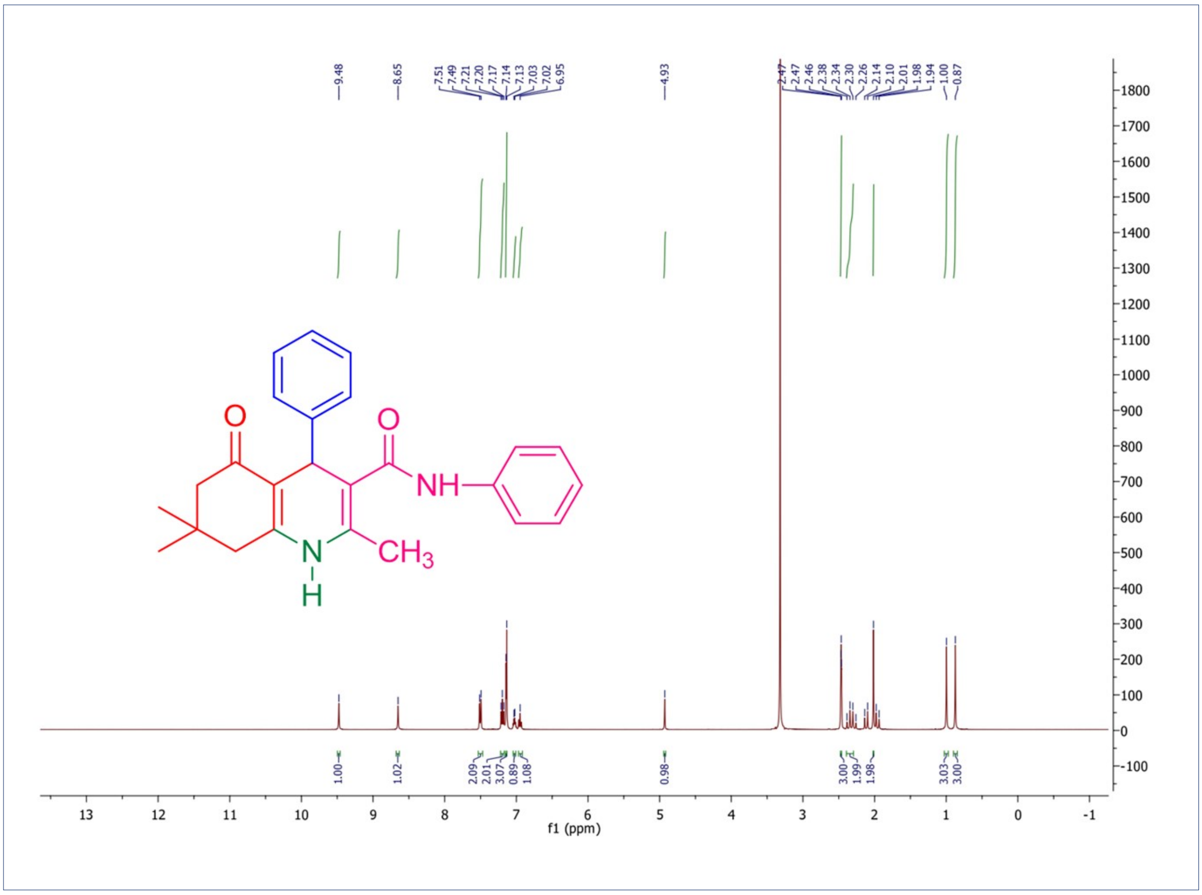

Spectrum 1 : ^{1}H NMR Spectrum of 4-(phenyl)-2,7,7-trimethyl-5-oxo-N-phenyl-1,4,5,6,7,8-hexahydroquinoline -3-carboxamide (5a)

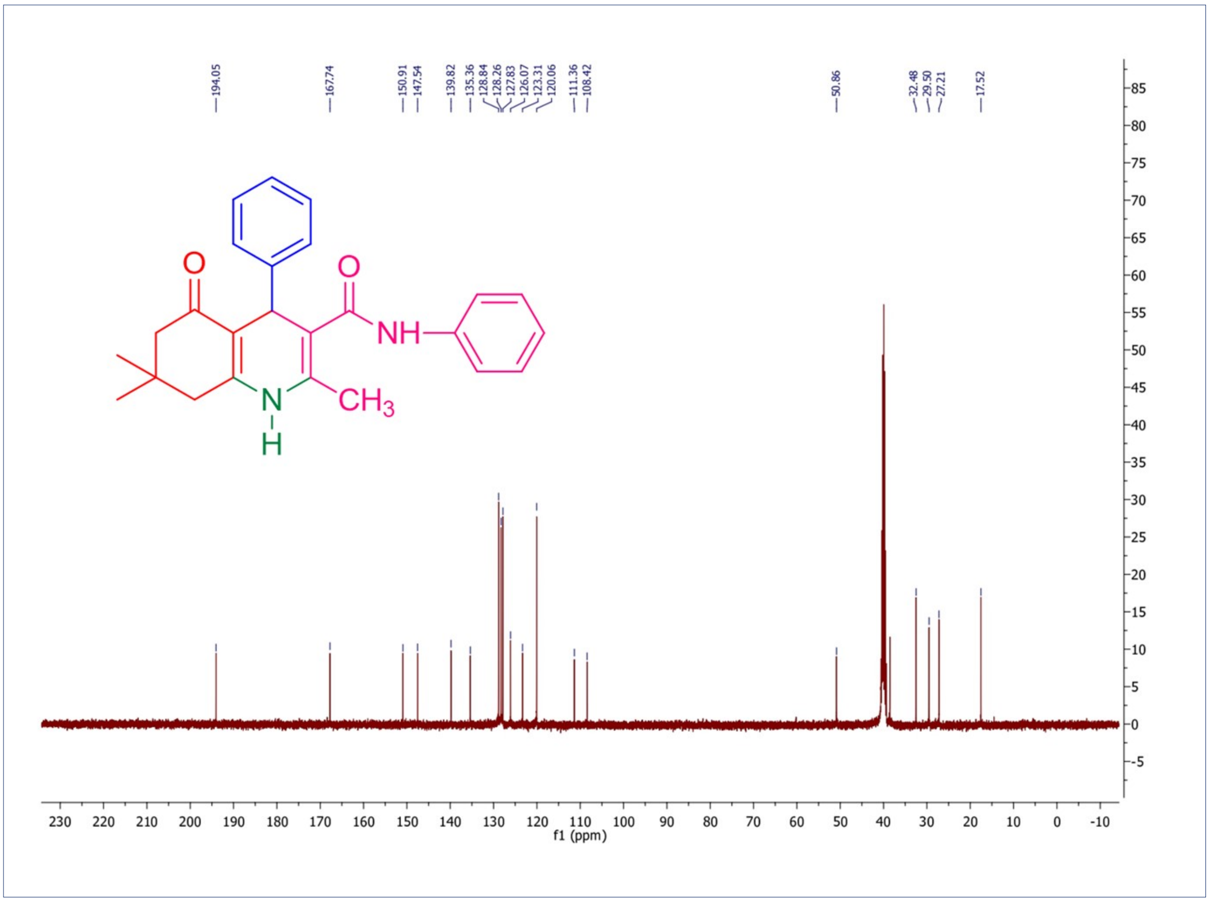

Spectrum 2 : ^{13}C NMR Spectrum of 4-(phenyl)-2,7,7-trimethyl-5-oxo-N-phenyl-1,4,5,6,7,8-hexahydroquinoline -3-carboxamide (5a)

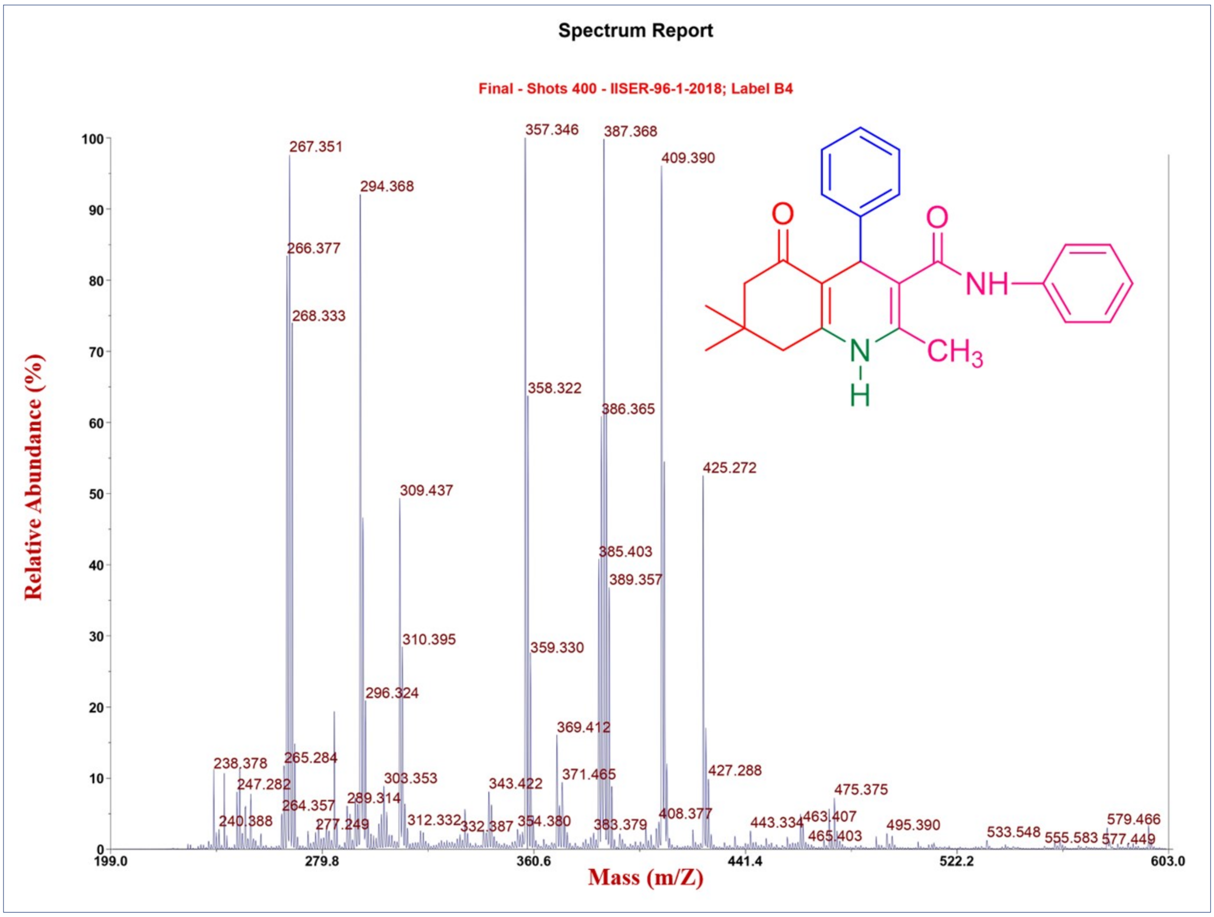

Spectrum 3 : Mass Spectrum of 4-(phenyl)-2,7,7-trimethyl-5-oxo-N-phenyl-1,4,5,6,7,8-hexahydroquinoline -3-carboxamide (5a)

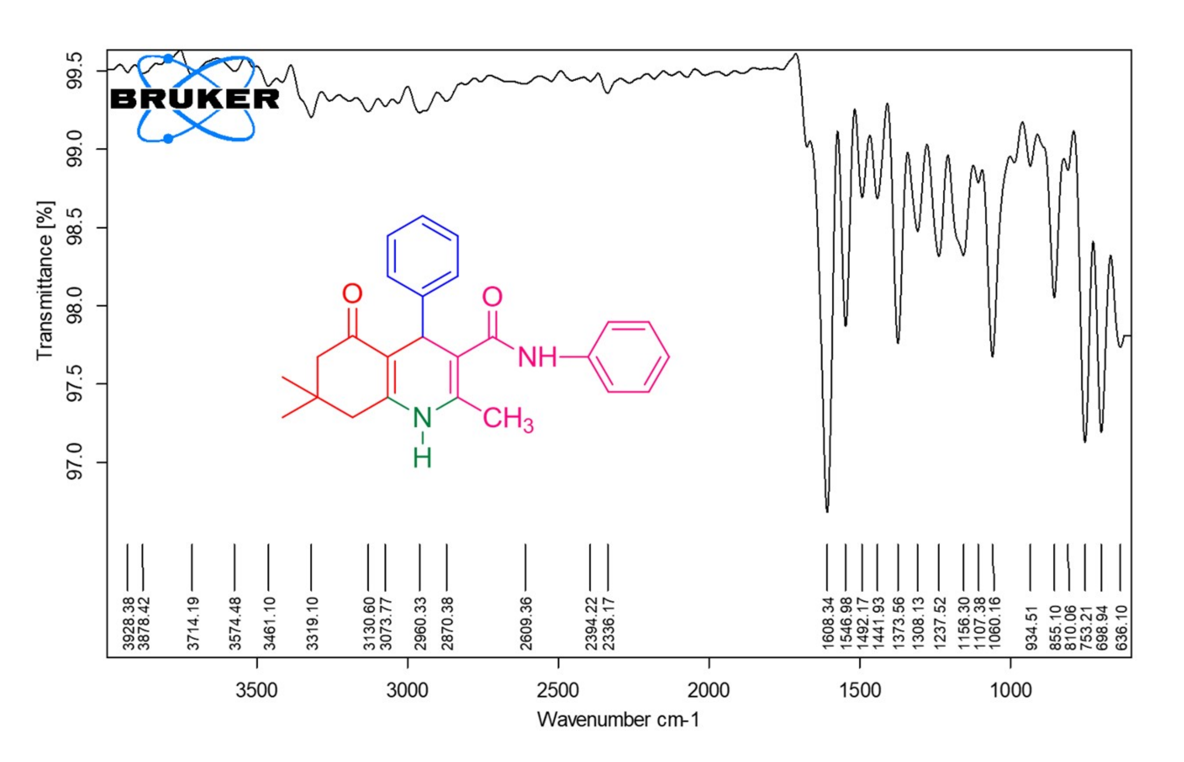

Spectrum 4 : FTIR Spectrum of 4-(phenyl)-2,7,7-trimethyl-5-oxo-N-phenyl-1,4,5,6,7,8-hexahydroquinoline -3-carboxamide (5a)

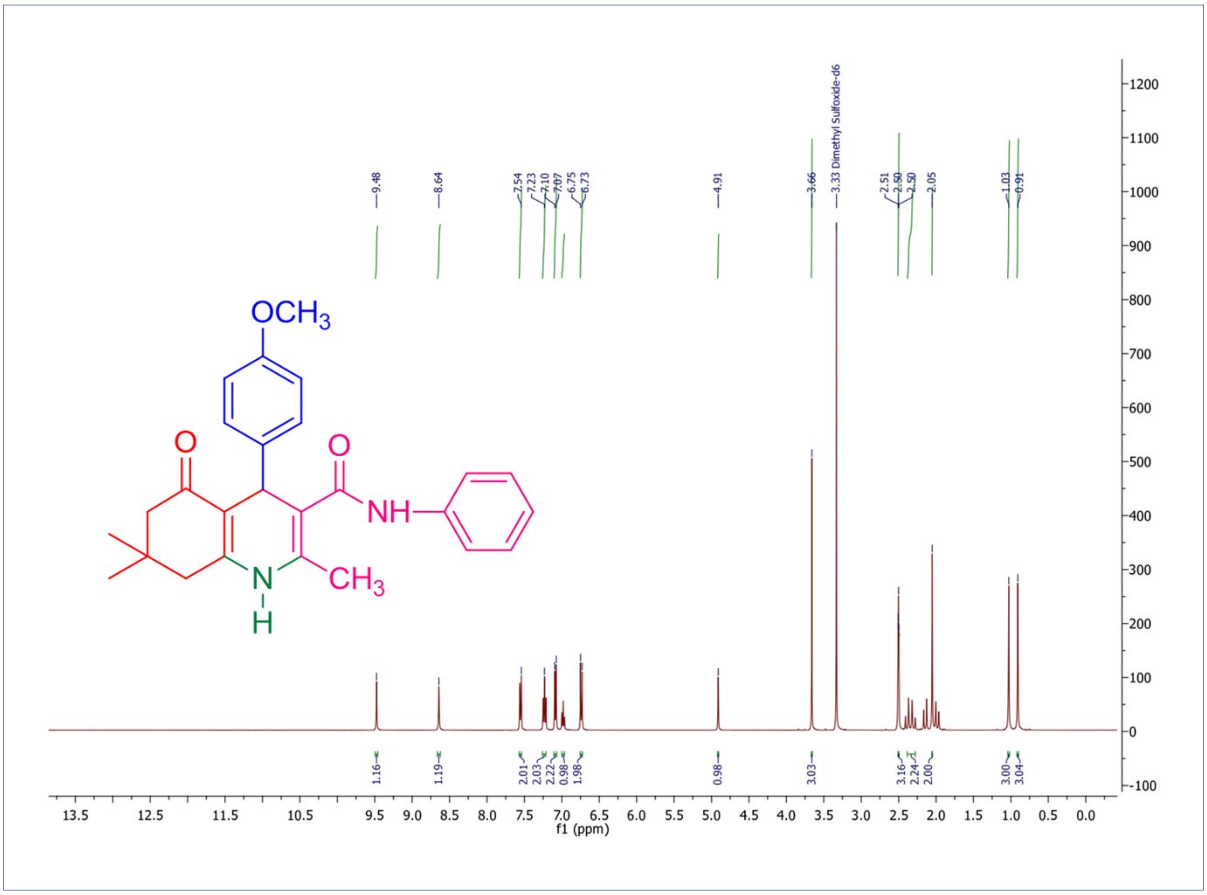

Spectrum 5 : ^{1}H NMR Spectrum of 4-(4-methoxyphenyl)-2,7,7-trimethyl-5-oxo-N-phenyl-1,4,5,6,7,8-hexahydroquinoline -3-carboxamide (5b)

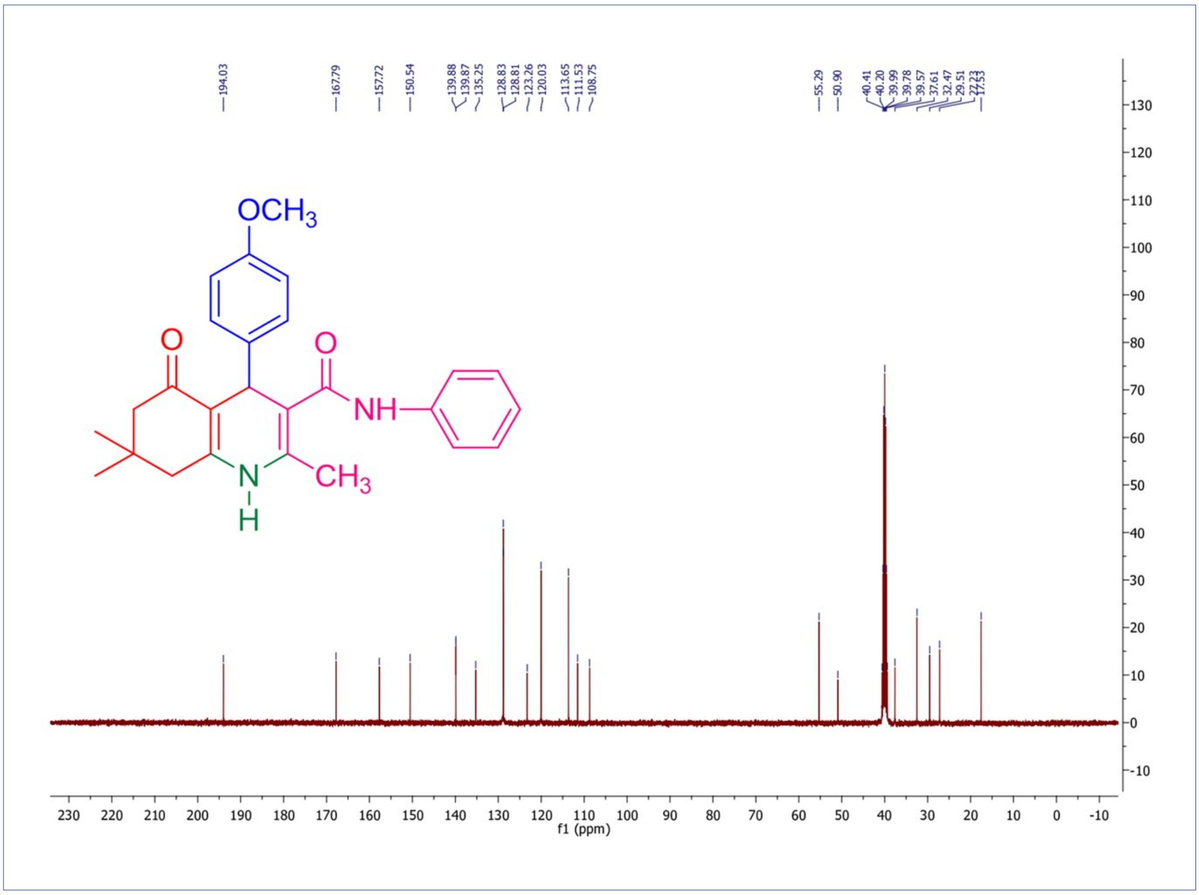

Spectrum 6 : ^{13}C NMR Spectrum of 4-(4-methoxyphenyl)-2,7,7-trimethyl-5-oxo-N-phenyl-1,4,5,6,7,8-hexahydroquinoline -3-carboxamide (5b)

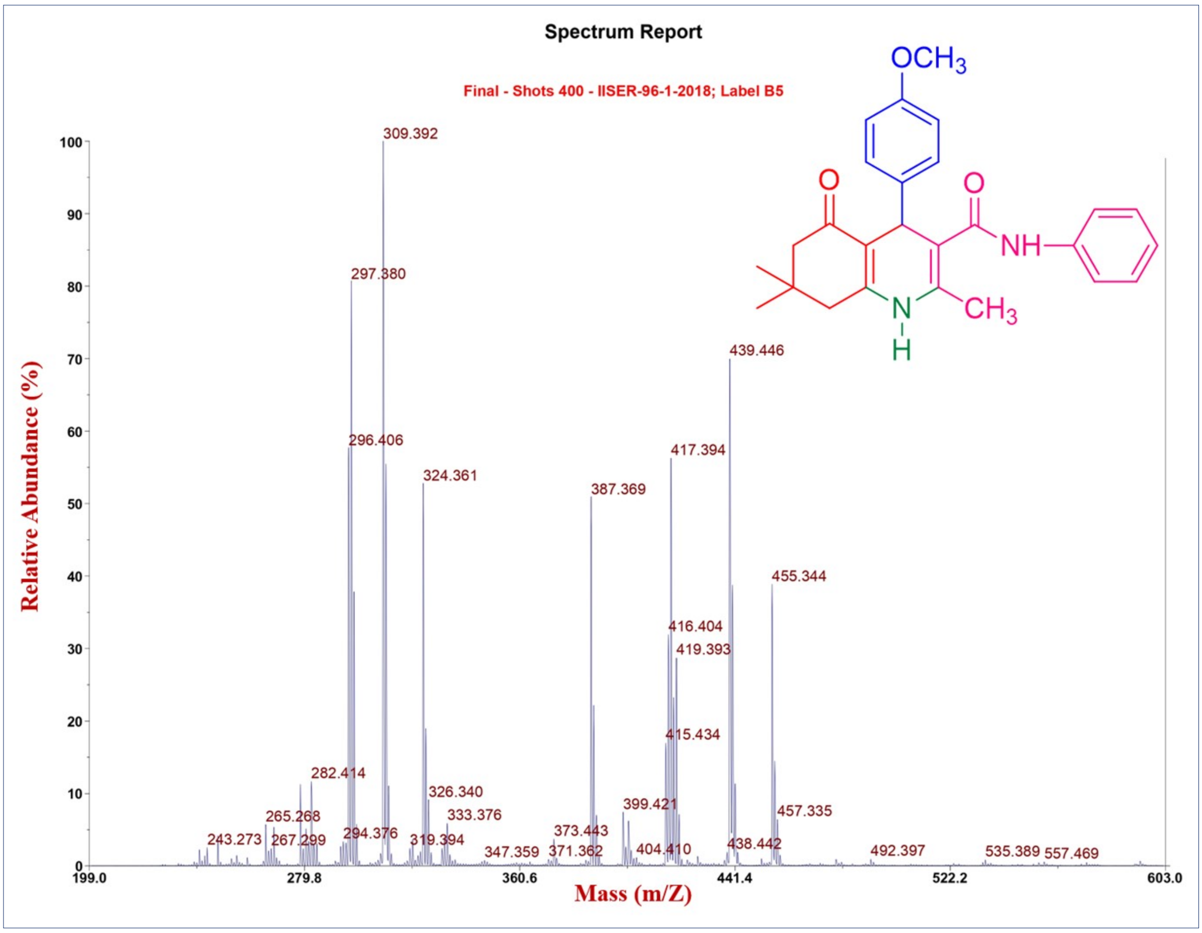

Spectrum 7 : Mass Spectrum of 4-(4-methoxyphenyl)-2,7,7-trimethyl-5-oxo-N-phenyl-1,4,5,6,7,8-hexahydroquinoline -3-carboxamide (5b)

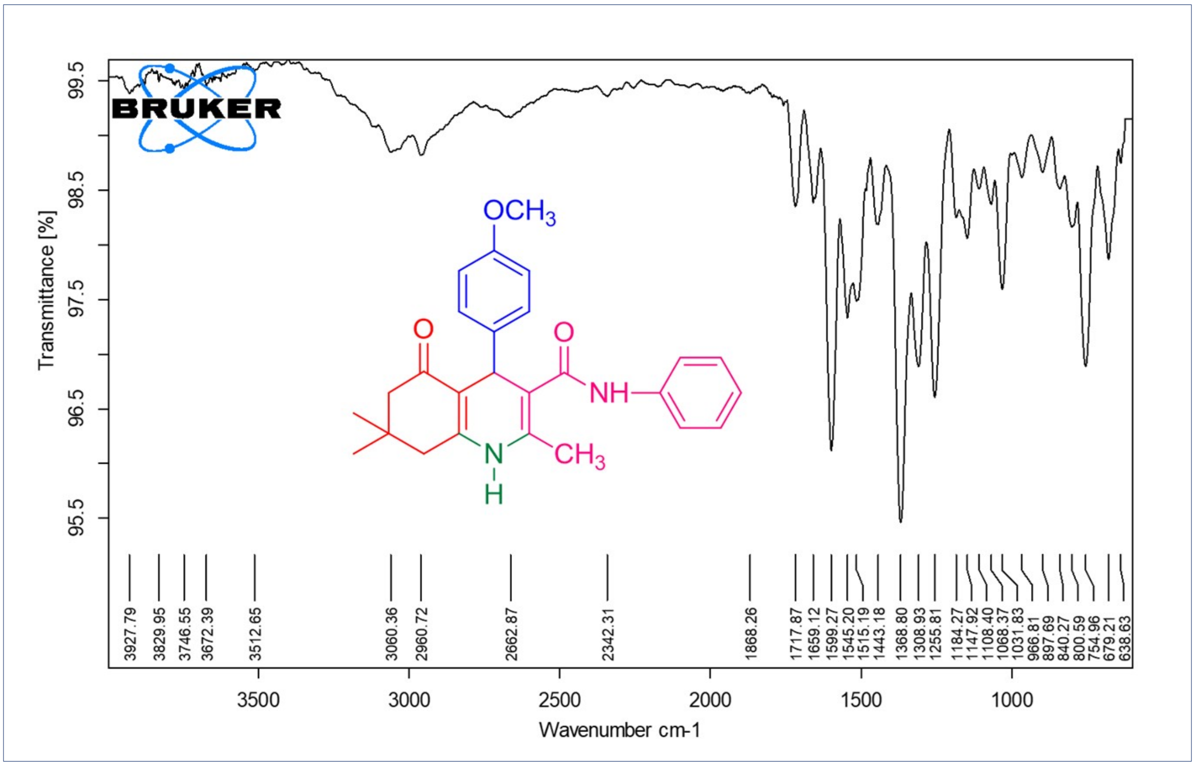

Spectrum 8 : FTIR Spectrum of 4-(4-methoxyphenyl)-2,7,7-trimethyl-5-oxo-N-phenyl-1,4,5,6,7,8-hexahydroquinoline -3-carboxamide (5b)

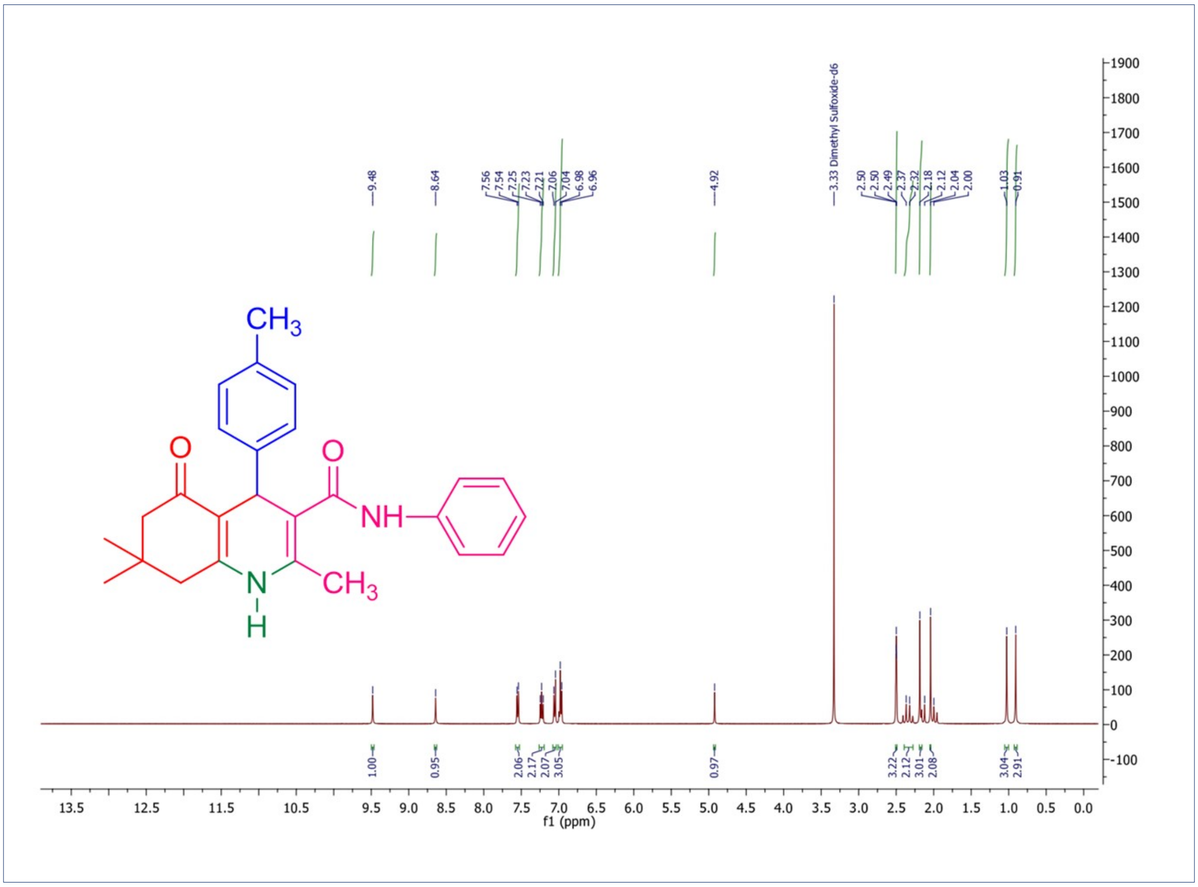

Spectrum 9 : ^{1}H Spectrum of 4-(4-methylphenyl)-2,7,7-trimethyl-5-oxo-N-phenyl-1,4,5,6,7,8-hexahydroquinoline -3-carboxamide (5c)

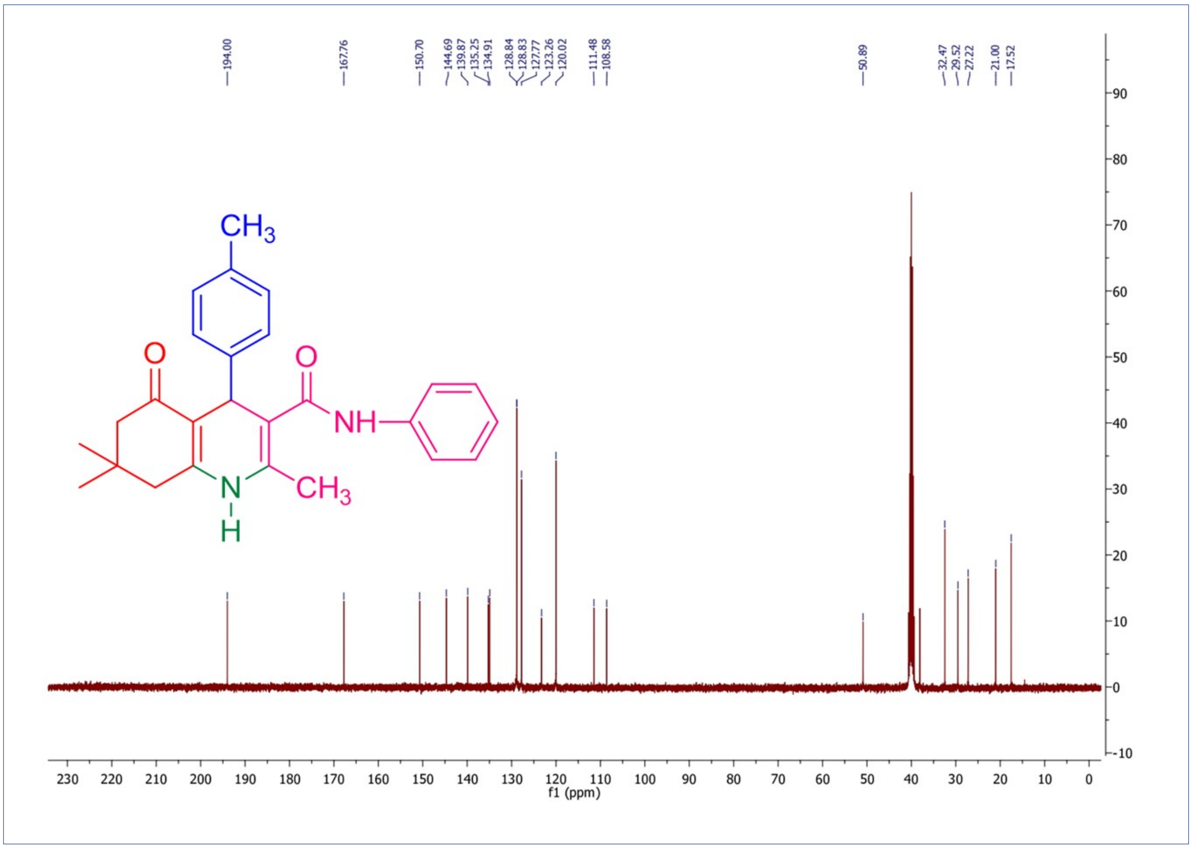

Spectrum 10 : ^{13}C Spectrum of 4-(4-methylphenyl)-2,7,7-trimethyl-5-oxo-N-phenyl-1,4,5,6,7,8-hexahydroquinoline -3-carboxamide (5c)

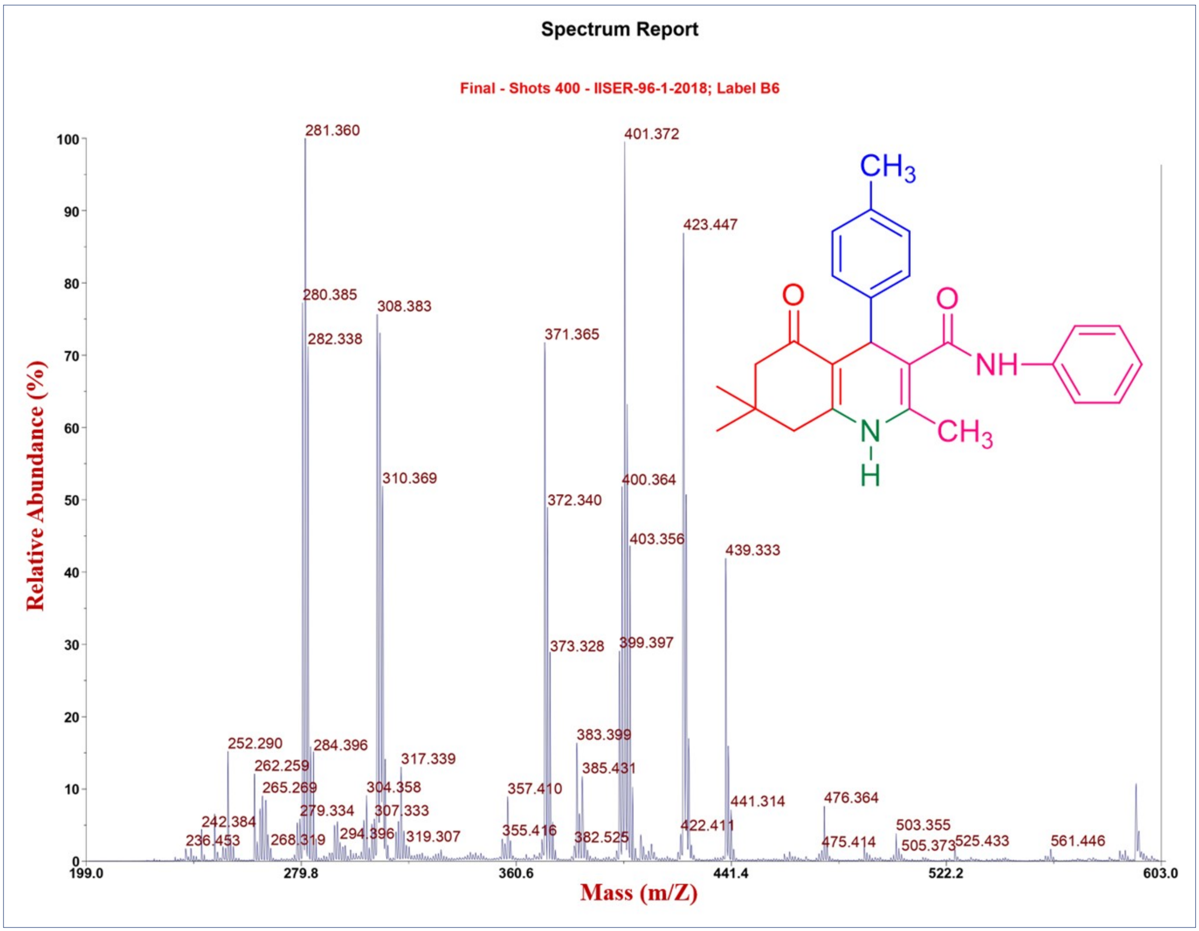

Spectrum 11 : Mass Spectrum of 4-(4-methylphenyl)-2,7,7-trimethyl-5-oxo-N-phenyl-1,4,5,6,7,8-hexahydroquinoline -3-carboxamide (5c)

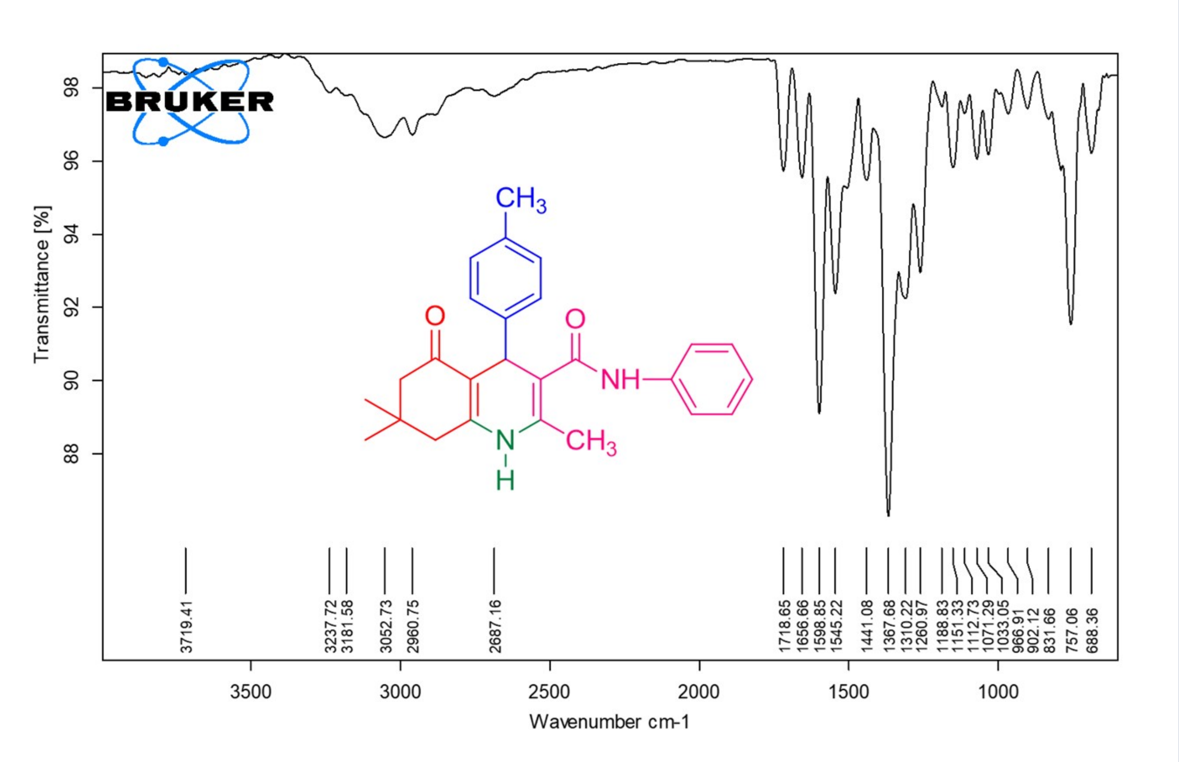

Spectrum 12 : FTIR Spectrum of 4-(4-methylphenyl)-2,7,7-trimethyl-5-oxo-N-phenyl-1,4,5,6,7,8-hexahydroquinoline -3-carboxamide (5c)

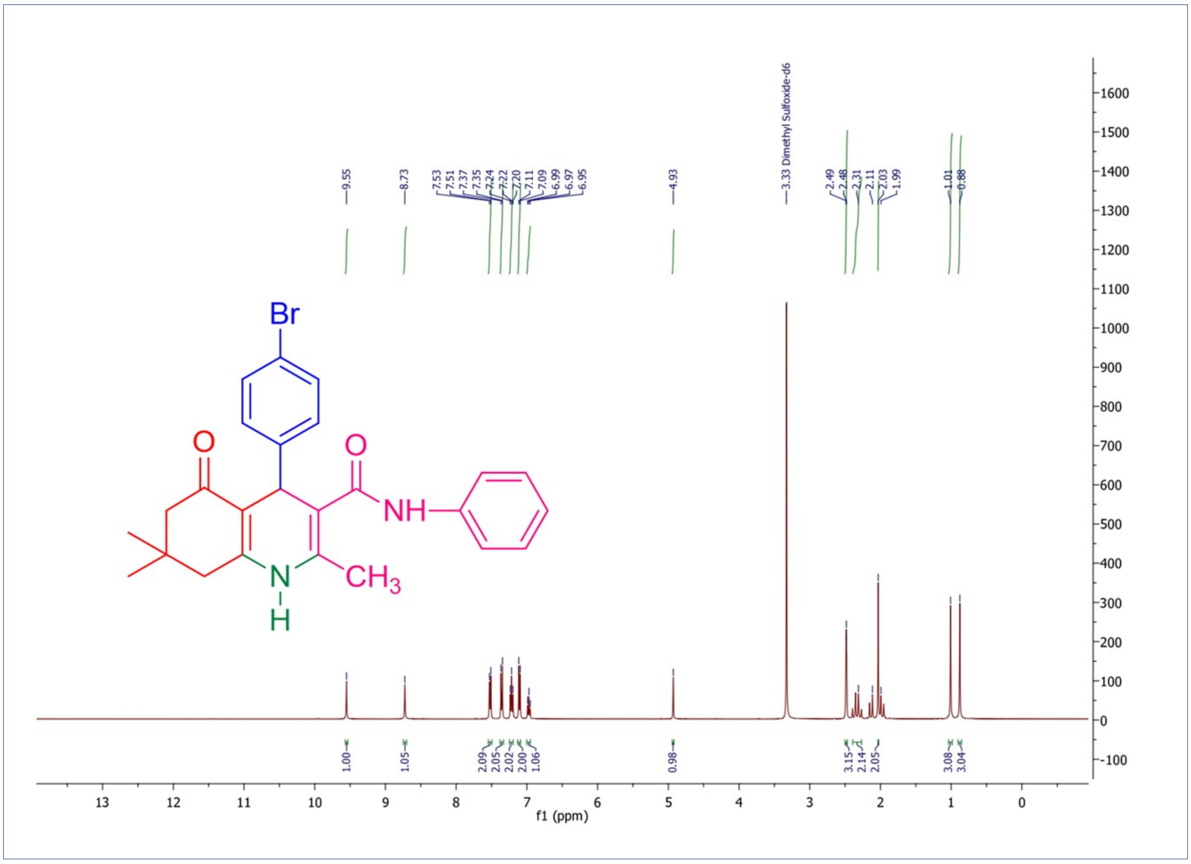

Spectrum 13 : ^{1}H Spectrum of 4-(4-bromophenyl)-2,7,7-trimethyl-5-oxo-N-phenyl-1,4,5,6,7,8-hexahydroquinoline -3-carboxamide (5d)

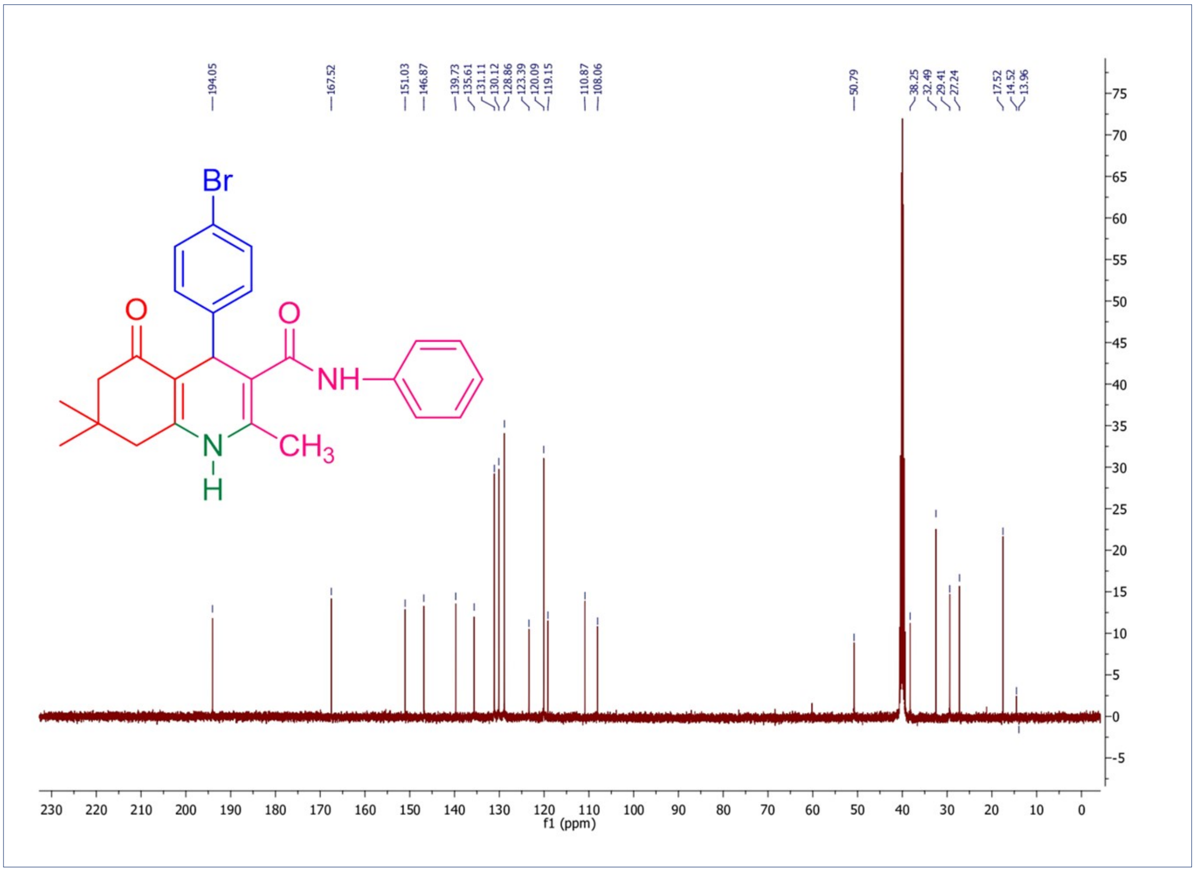

Spectrum 14 : ^{13}C Spectrum of 4-(4-bromophenyl)-2,7,7-trimethyl-5-oxo-N-phenyl-1,4,5,6,7,8-hexahydroquinoline -3-carboxamide (5d)

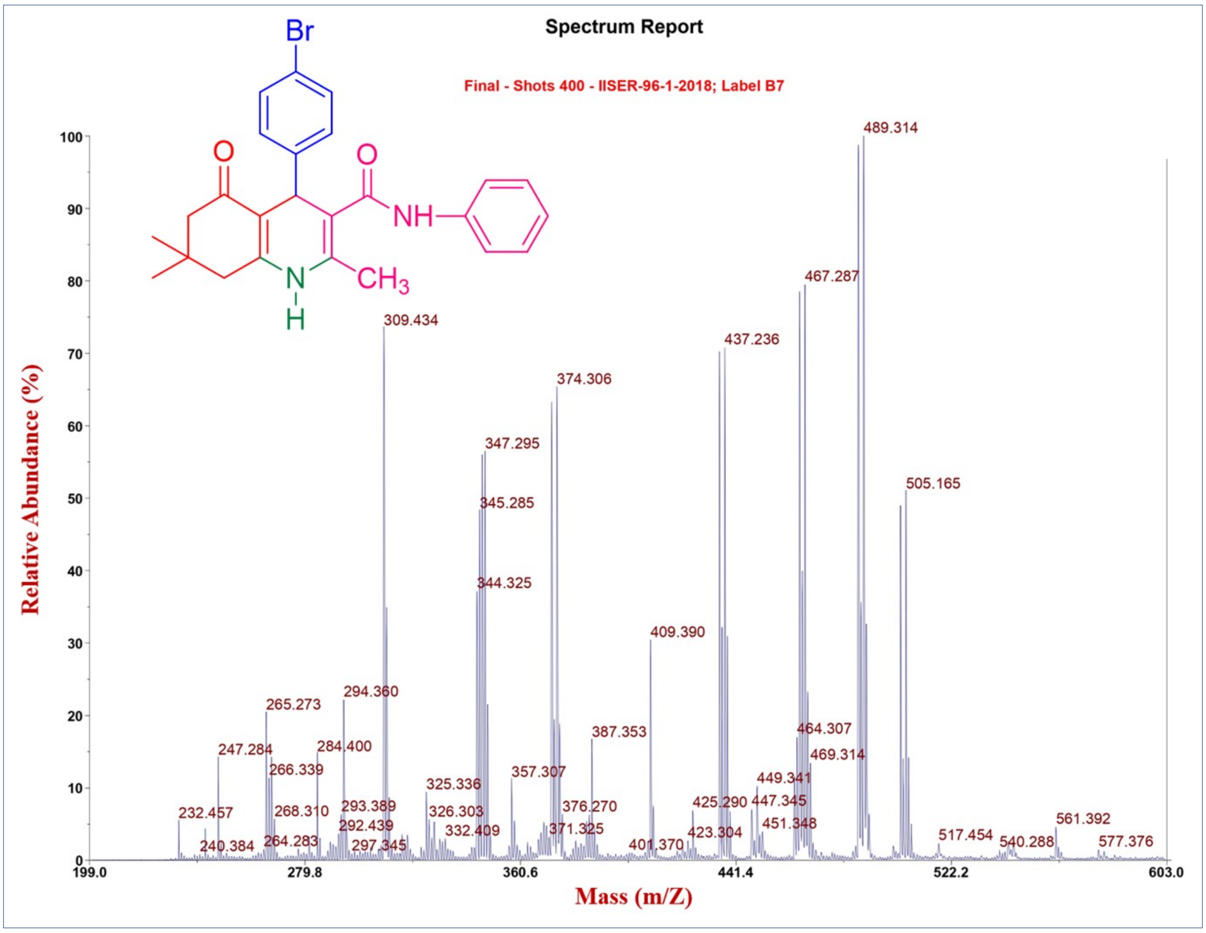

Spectrum 15 : Mass Spectrum of 4-(4-bromophenyl)-2,7,7-trimethyl-5-oxo-N-phenyl-1,4,5,6,7,8-hexahydroquinoline -3-carboxamide (5d)

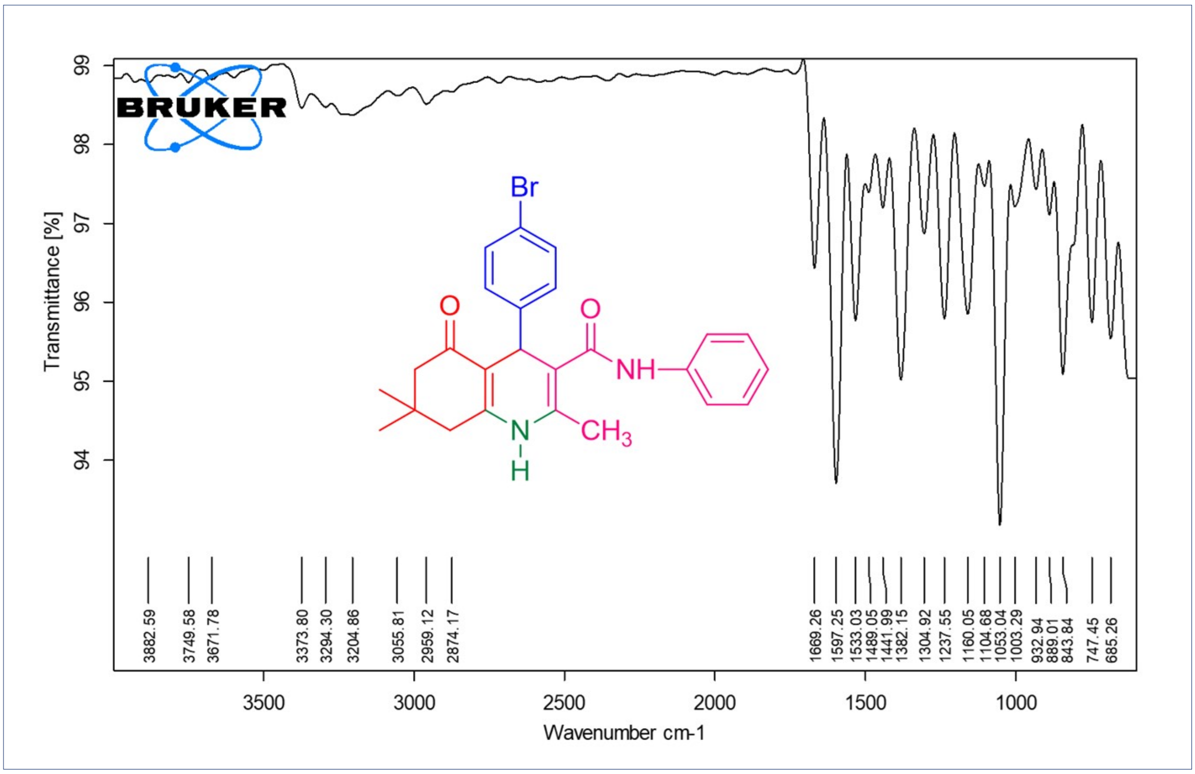

Spectrum 16 : FTIR Spectrum of 4-(4-bromophenyl)-2,7,7-trimethyl-5-oxo-N-phenyl-1,4,5,6,7,8-hexahydroquinoline -3-carboxamide (5d)

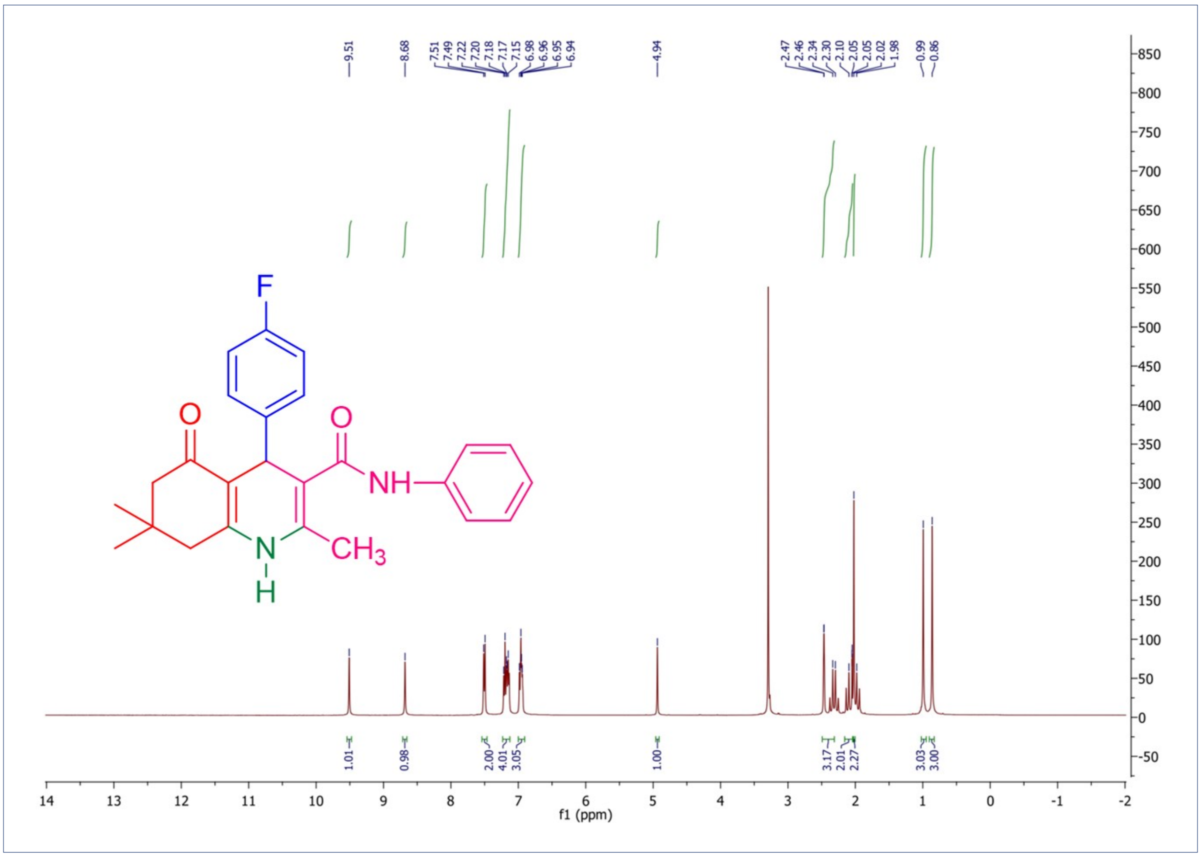

Spectrum 17 : ^{1}H Spectrum of 4-(4-fluorophenyl)-2,7,7-trimethyl-5-oxo-N-phenyl-1,4,5,6,7,8-hexahydroquinoline -3-carboxamide (5e)

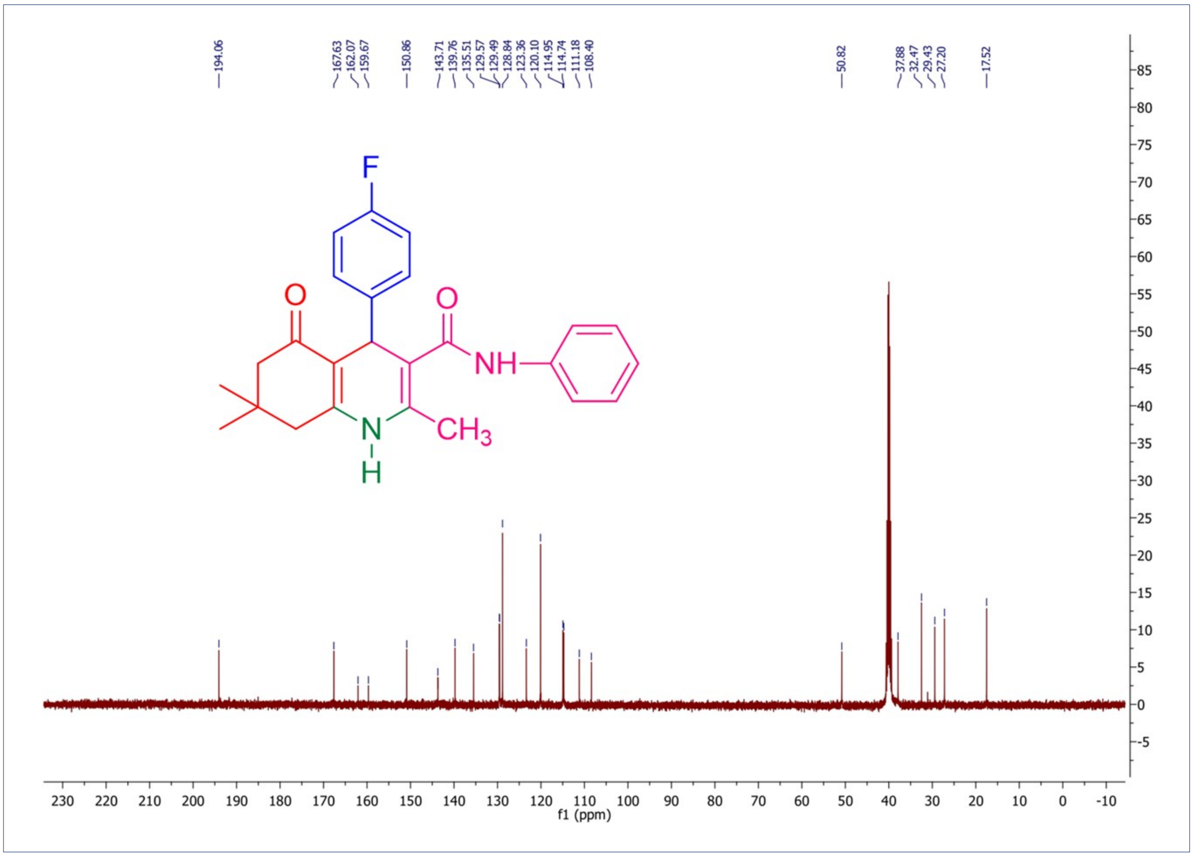

Spectrum 18 : ^{13}C Spectrum of 4-(4-fluorophenyl)-2,7,7-trimethyl-5-oxo-N-phenyl-1,4,5,6,7,8-hexahydroquinoline -3-carboxamide (5e)

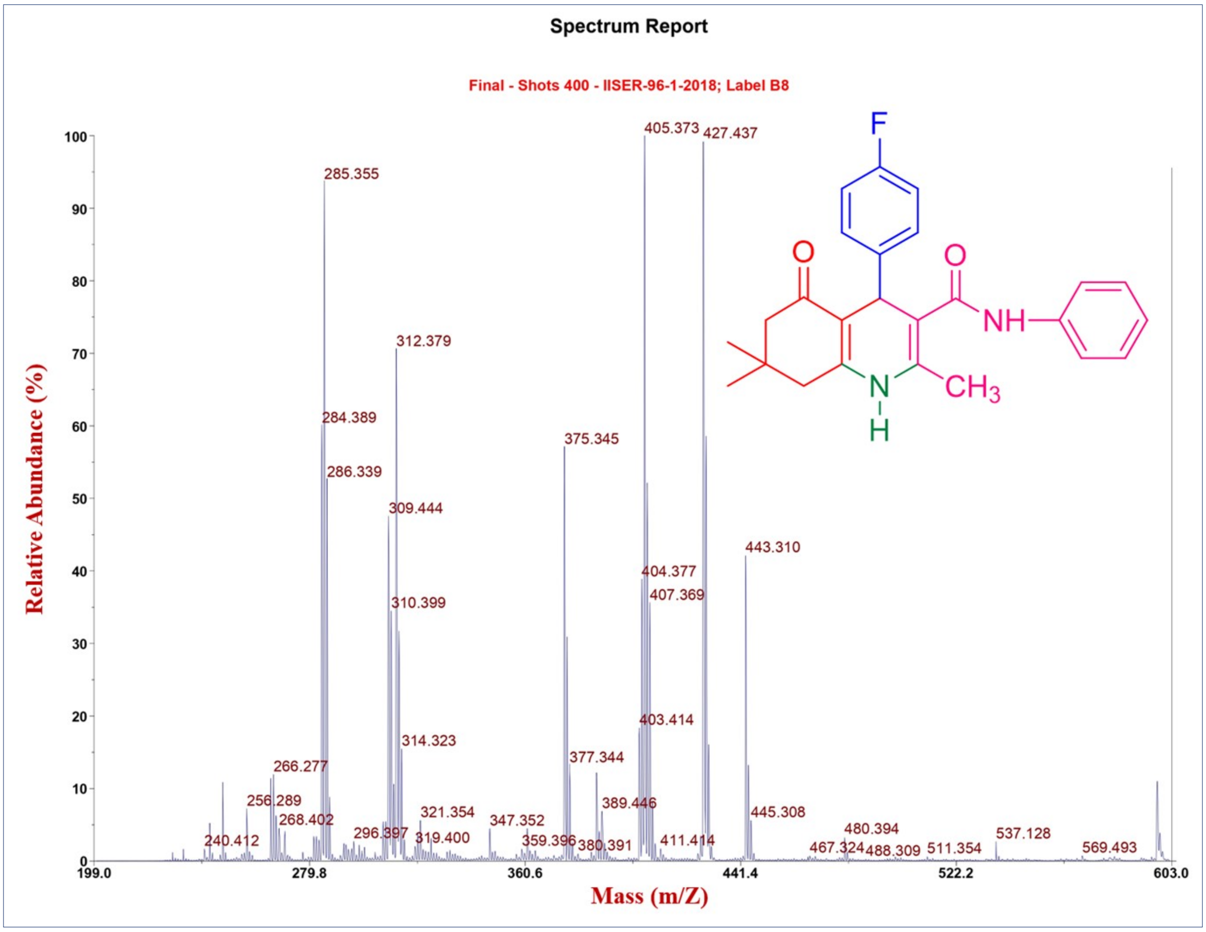

Spectrum 19 : Mass Spectrum of 4-(4-fluorophenyl)-2,7,7-trimethyl-5-oxo-N-phenyl-1,4,5,6,7,8-hexahydroquinoline -3-carboxamide (5e)

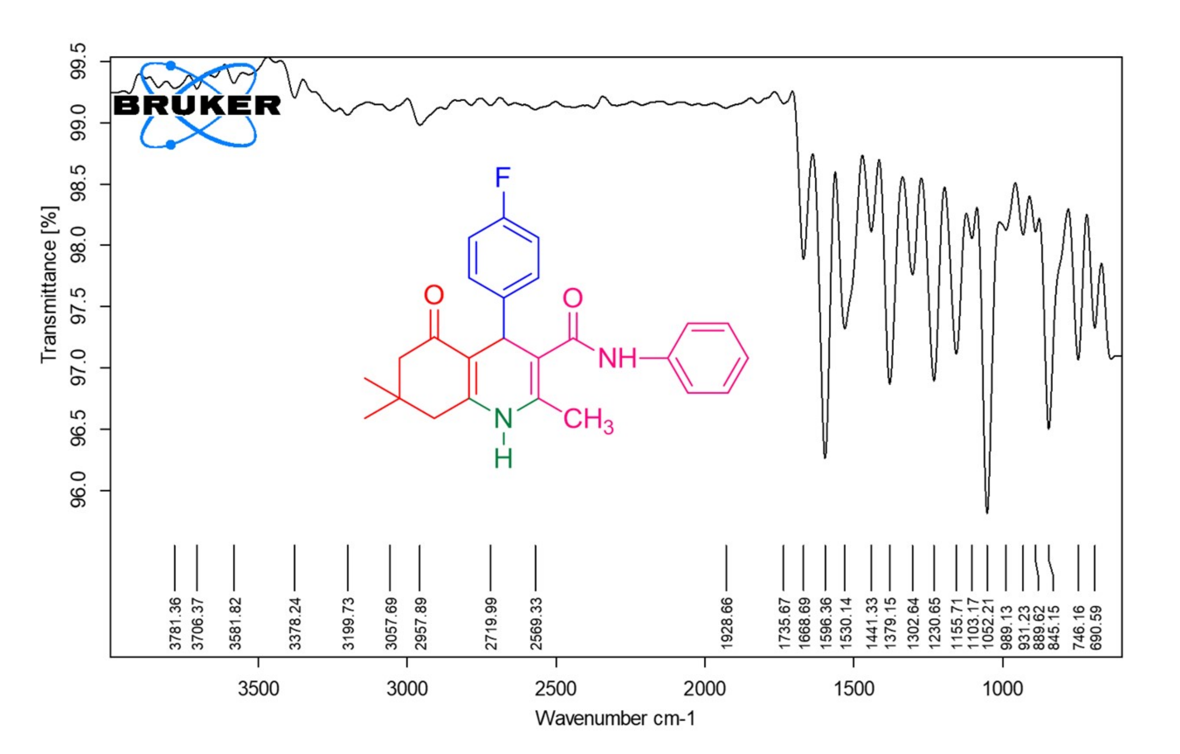

Spectrum 20 : FTIR Spectrum of 4-(4-fluorophenyl)-2,7,7-trimethyl-5-oxo-N-phenyl-1,4,5,6,7,8-hexahydroquinoline -3-carboxamide (5e)

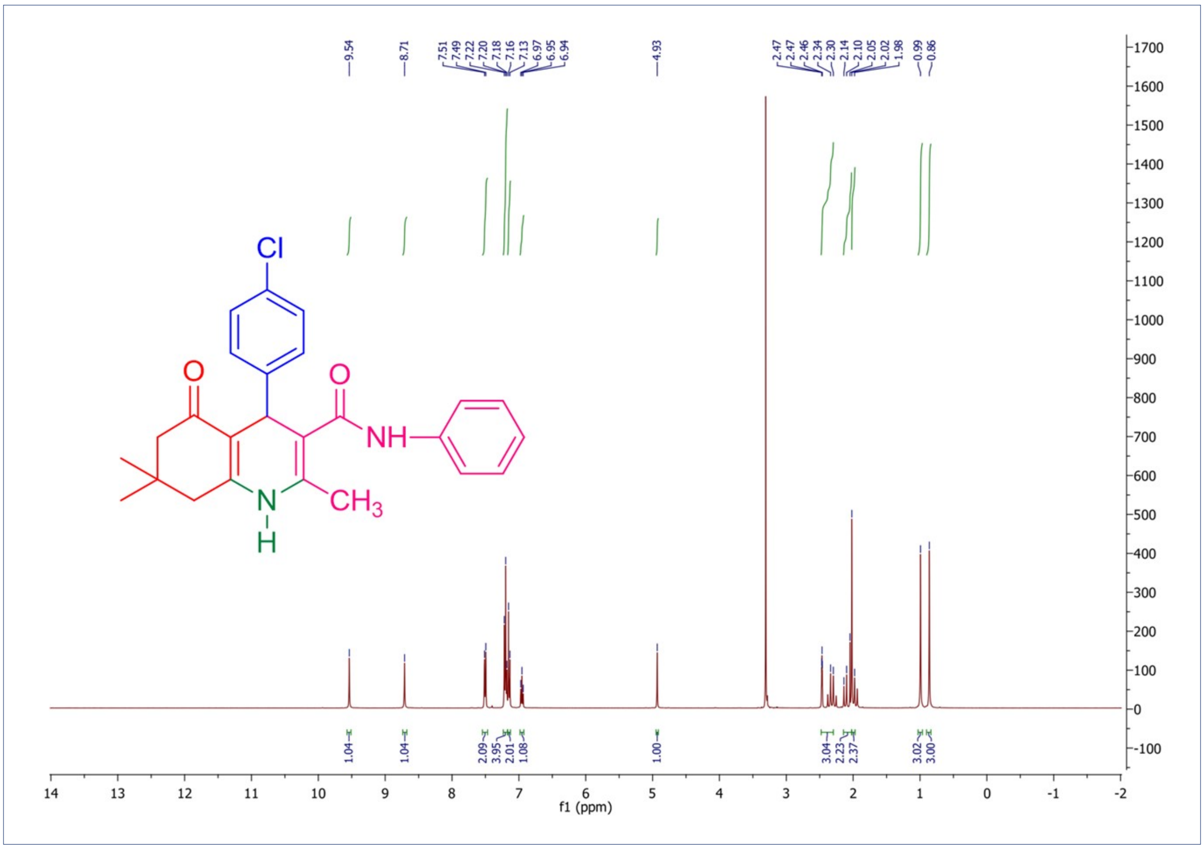

Spectrum 21 : ^{1}H Spectrum of 4-(4-chlorophenyl)-2,7,7-trimethyl-5-oxo-N-phenyl-1,4,5,6,7,8-hexahydroquinoline -3-carboxamide (5f)

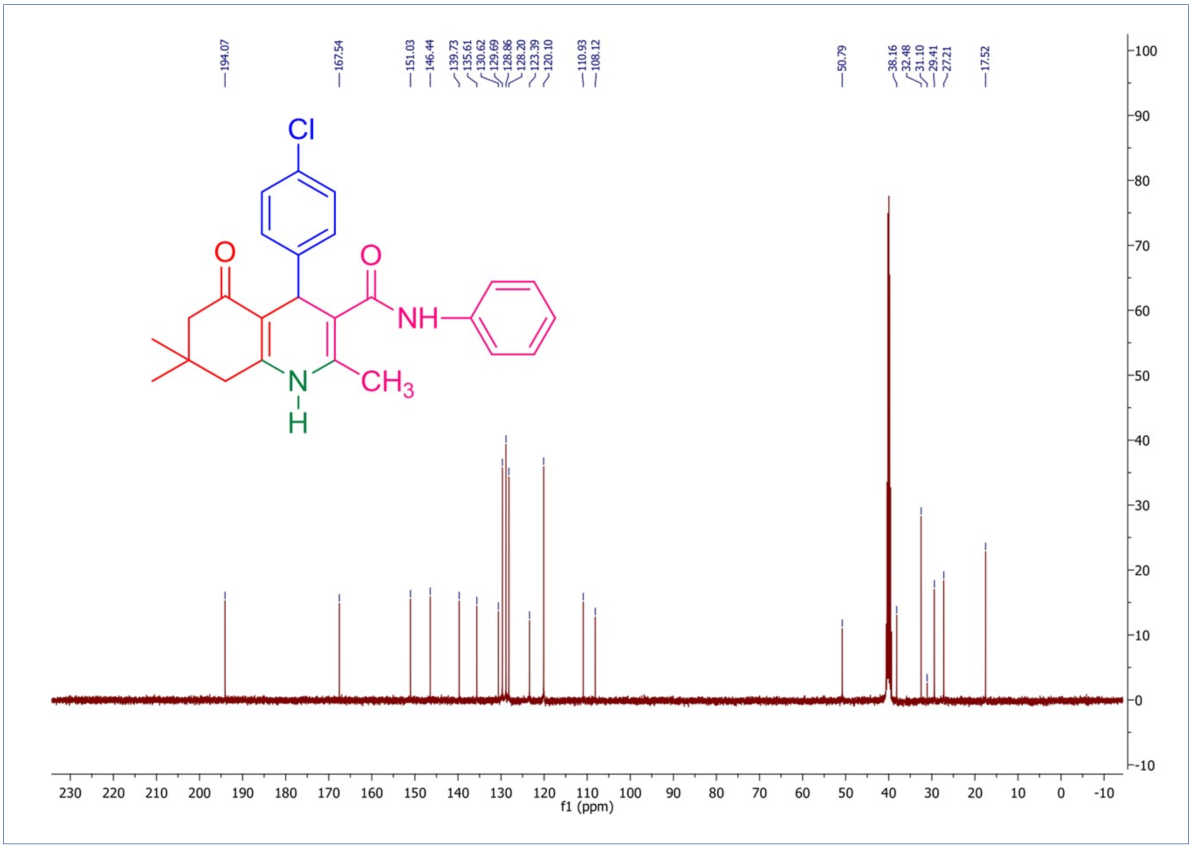

Spectrum 22 : ^{13}C Spectrum of 4-(4-chlorophenyl)-2,7,7-trimethyl-5-oxo-N-phenyl-1,4,5,6,7,8-hexahydroquinoline -3-carboxamide (5f)

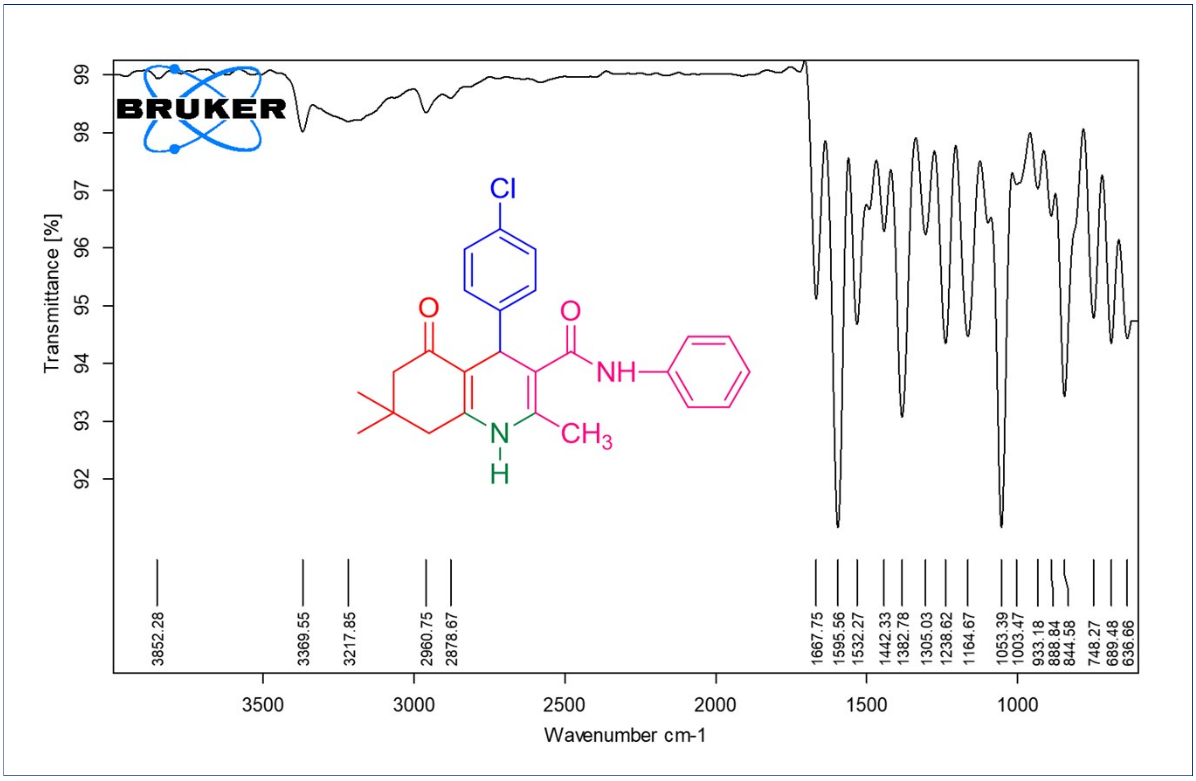

Spectrum 23 : FTIR Spectrum of 4-(4-chlorophenyl)-2,7,7-trimethyl-5-oxo-N-phenyl-1,4,5,6,7,8-hexahydroquinoline -3-carboxamide (5f)

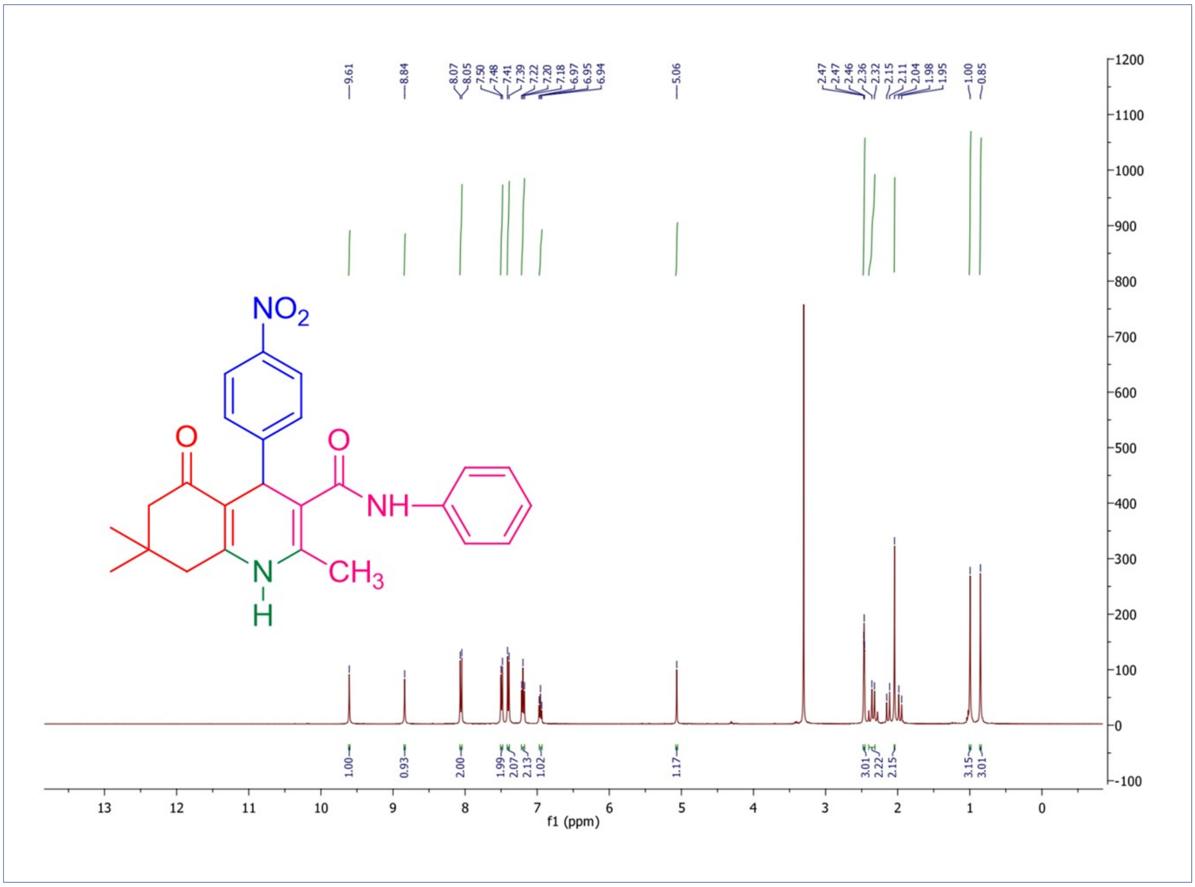

Spectrum 24 : ^{1}H Spectrum of 4-(4-nitrophenyl)-2,7,7-trimethyl-5-oxo-N-phenyl-1,4,5,6,7,8-hexahydroquinoline -3-carboxamide (5g)

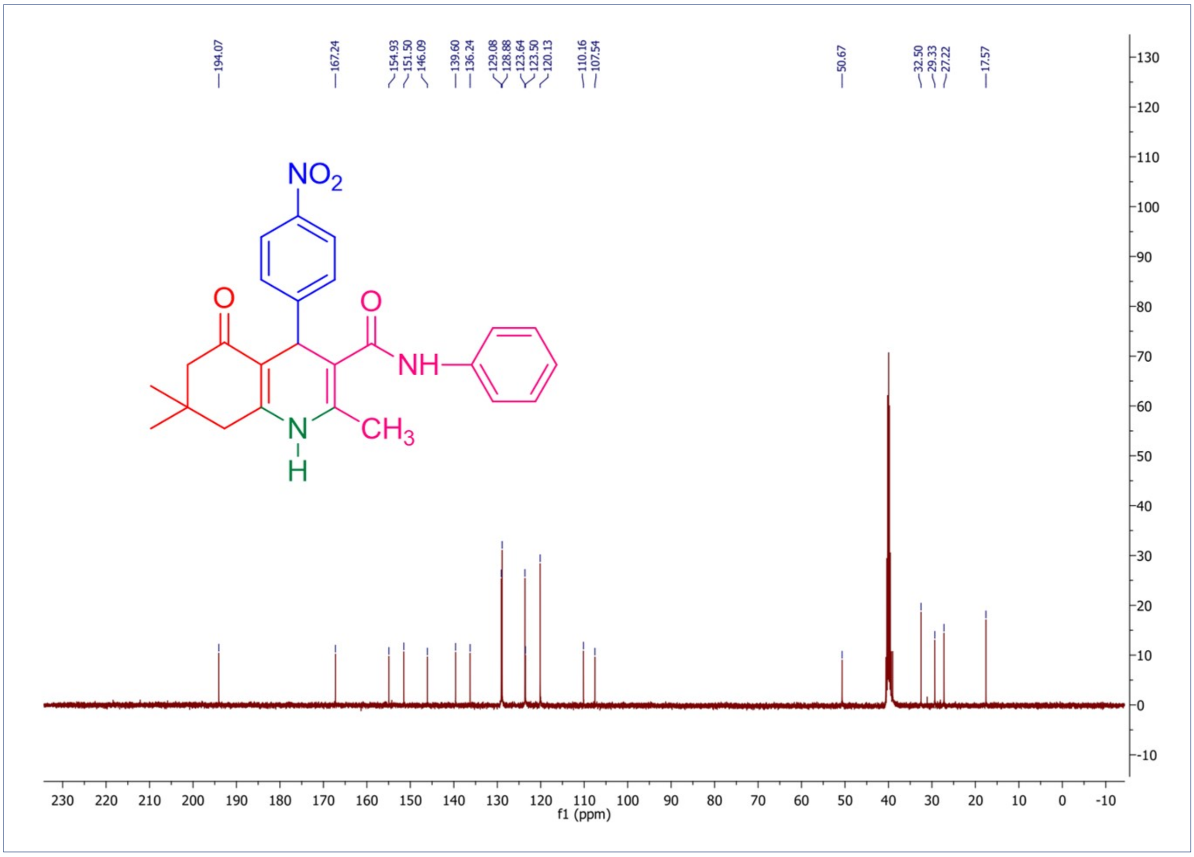

Spectrum 25 : [13]C Spectrum of 4-(4-nitrophenyl)-2,7,7-trimethyl-5-oxo-N-phenyl-1,4,5,6,7,8-hexahydroquinoline -3-carboxamide (5g)

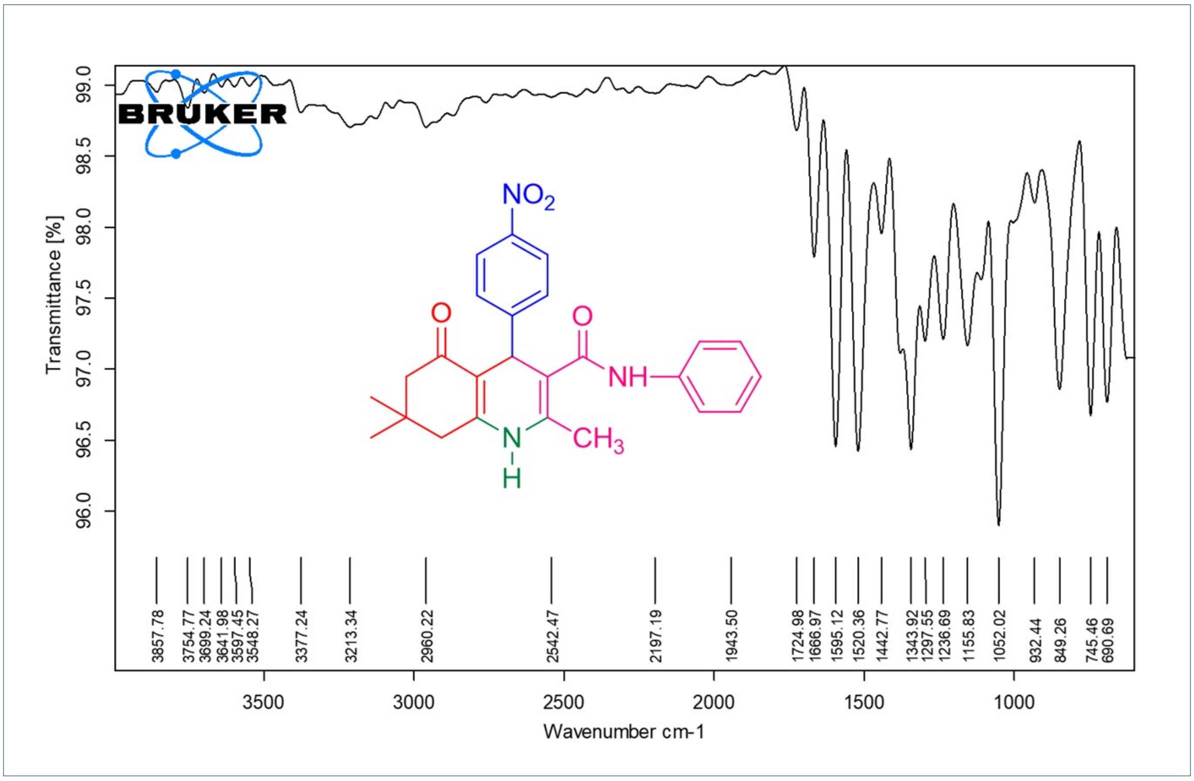

Spectrum 26 : FTIR Spectrum of 4-(4-nitrophenyl)-2,7,7-trimethyl-5-oxo-N-phenyl-1,4,5,6,7,8-hexahydroquinoline -3-carboxamide (5g)

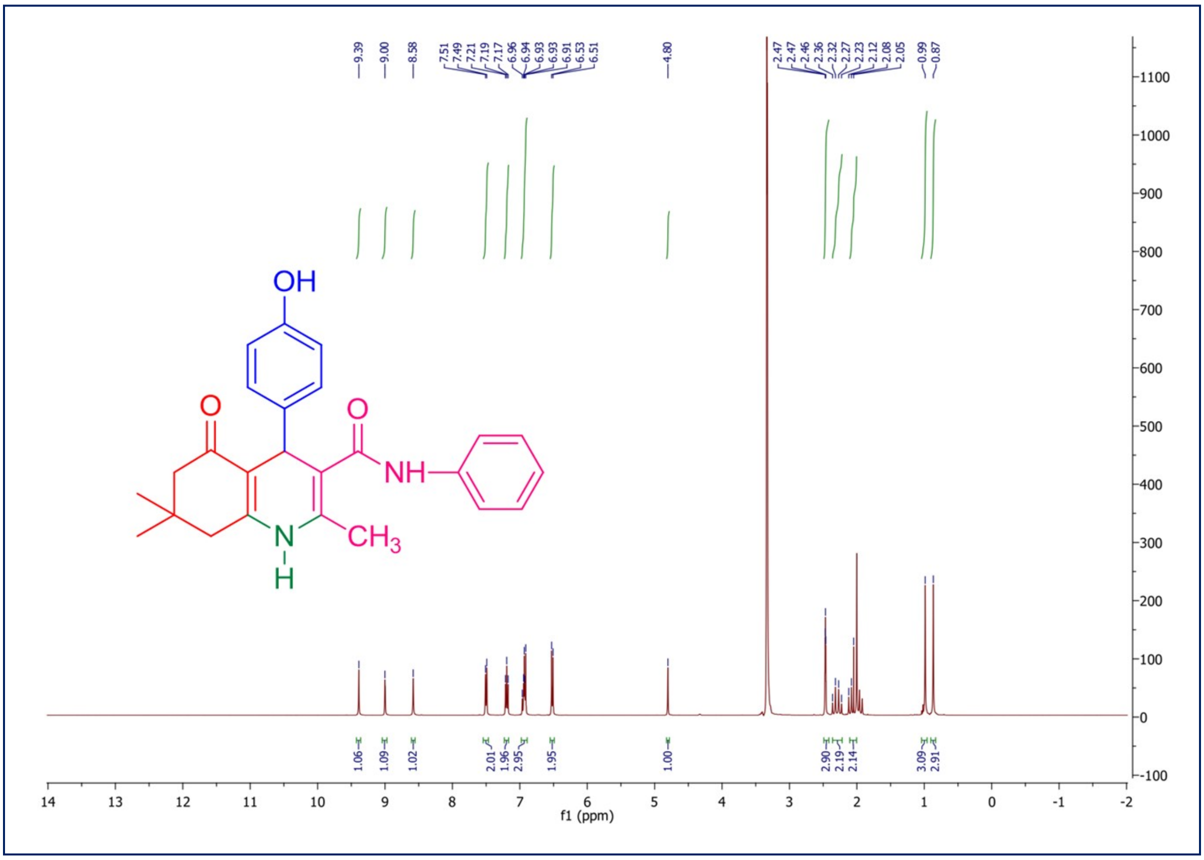

Spectrum 27 : ^{1}H Spectrum of 4-(4-hydroxyphenyl)-2,7,7-trimethyl-5-oxo-N-phenyl-1,4,5,6,7,8-hexahydroquinoline -3-carboxamide (5h)

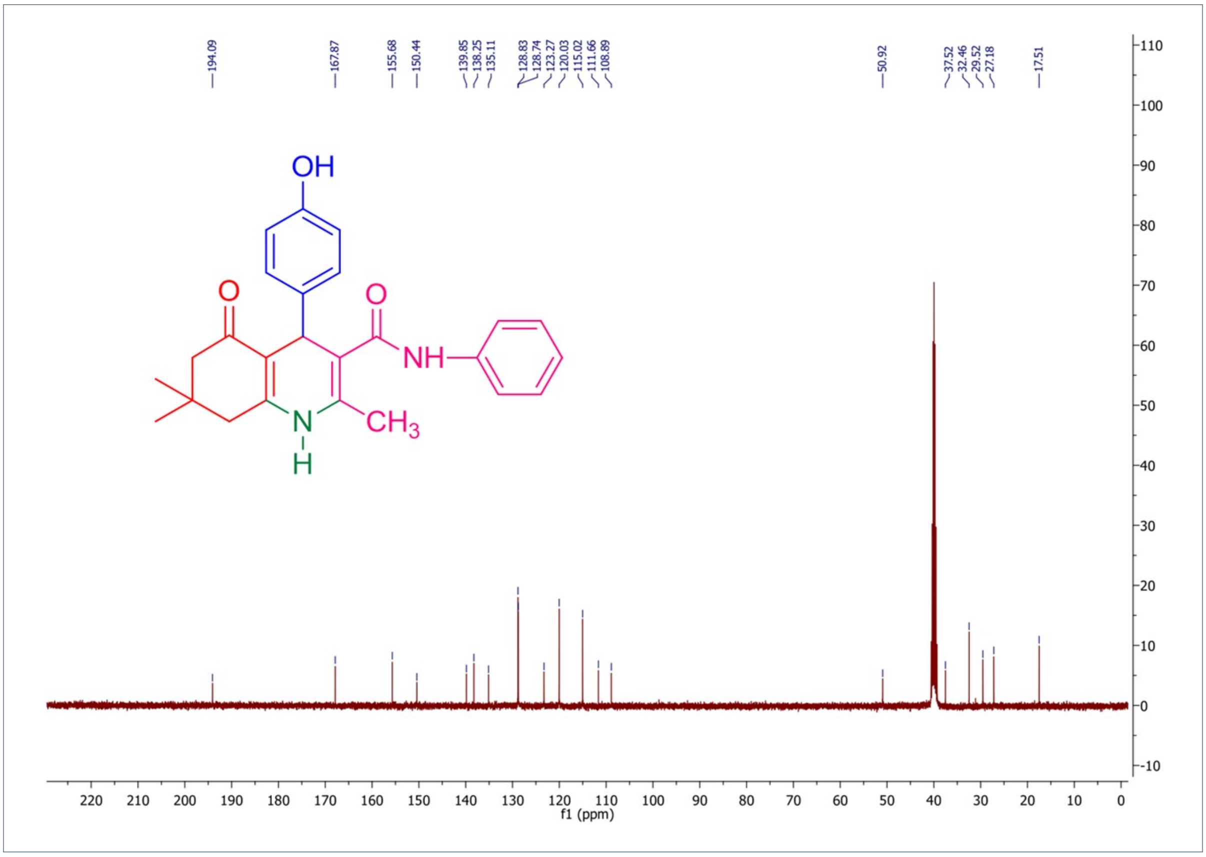

Spectrum 28 : ^{13}C Spectrum of 4-(4-hydroxyphenyl)-2,7,7-trimethyl-5-oxo-N-phenyl-1,4,5,6,7,8-hexahydroquinoline -3-carboxamide (5h)

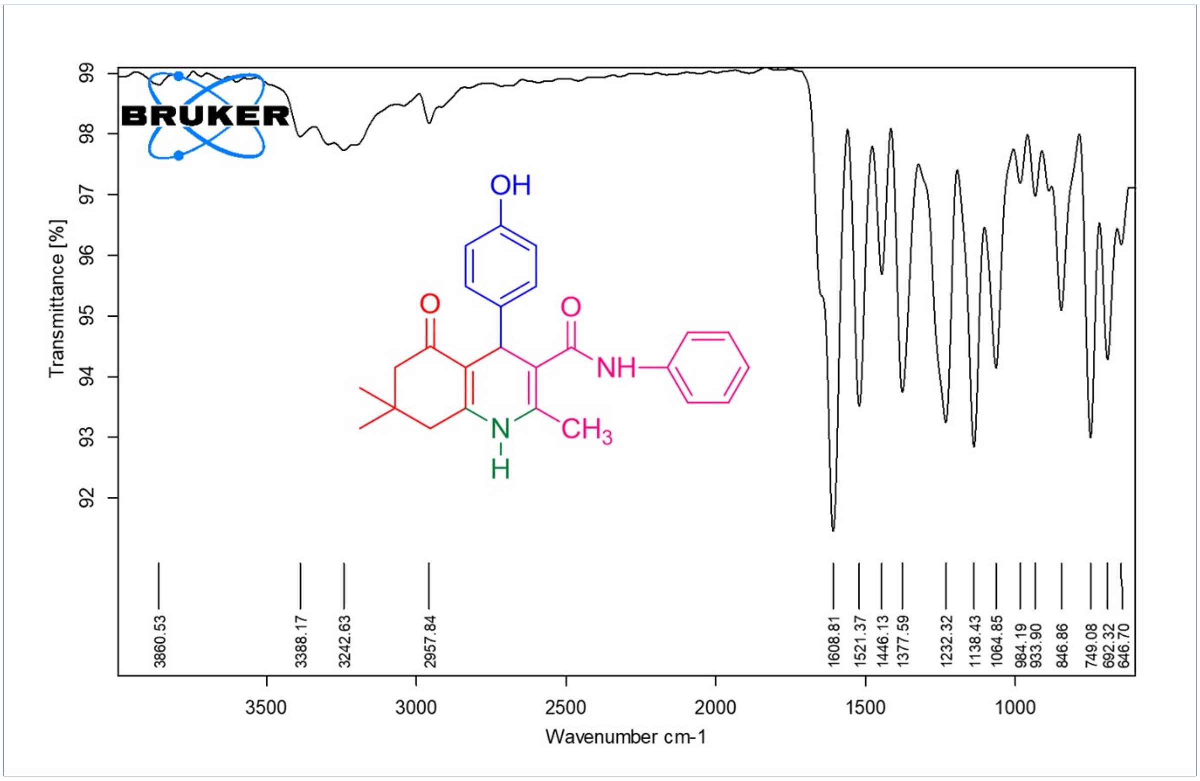

Spectrum 29 : FTIR Spectrum of 4-(4-hydroxyphenyl)-2,7,7-trimethyl-5-oxo-N-phenyl-1,4,5,6,7,8-hexahydroquinoline -3-carboxamide (5h)

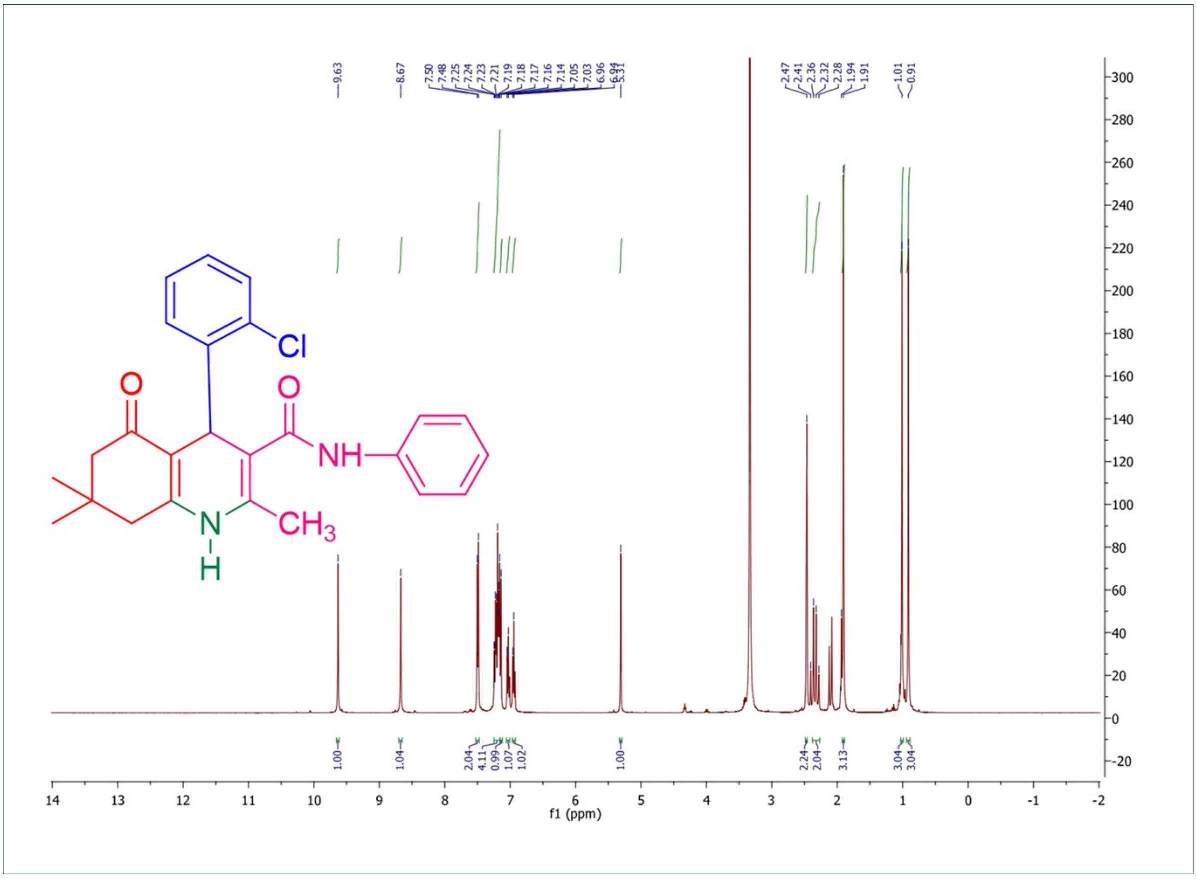

Spectrum 30 : ^{1}H Spectrum of 4-(2-chlorophenyl)-2,7,7-trimethyl-5-oxo-N-phenyl-1,4,5,6,7,8-hexahydroquinoline -3-carboxamide (5i)

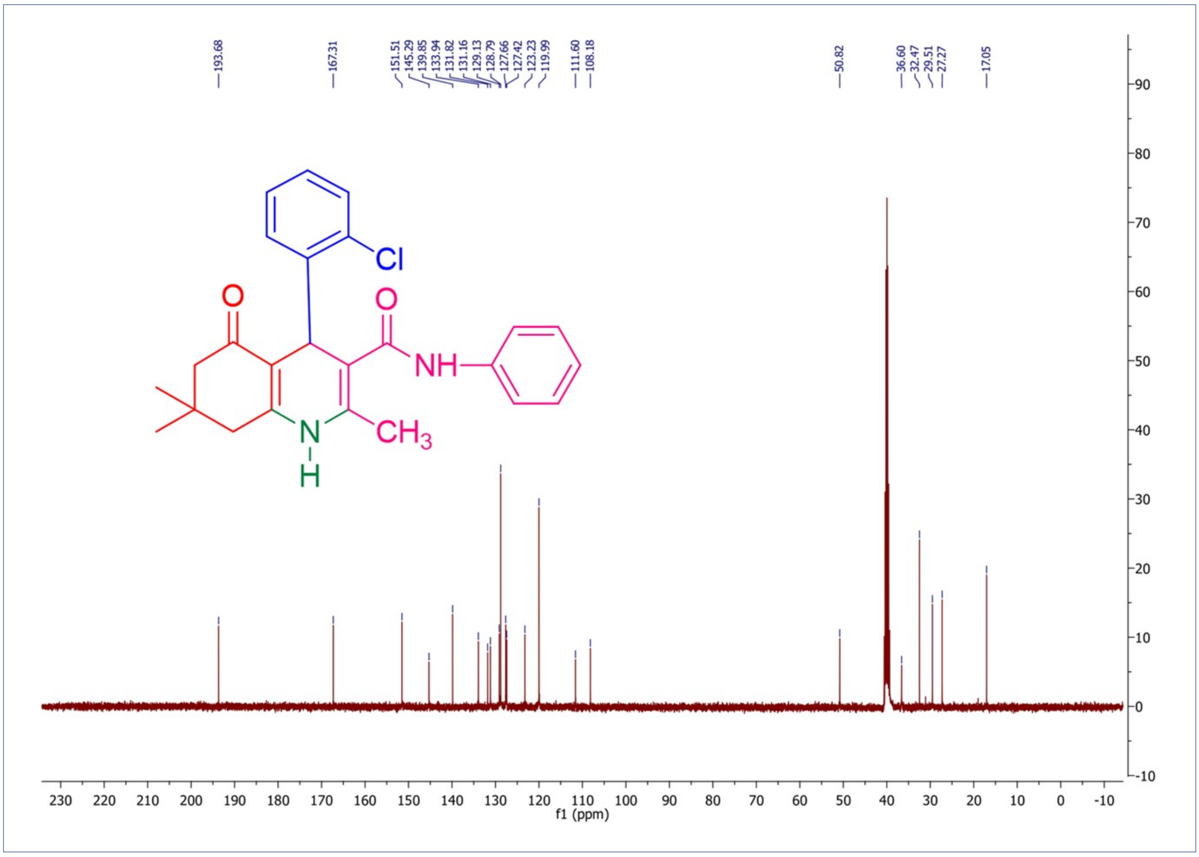

Spectrum 31 : ^{13}C Spectrum of 4-(2-chlorophenyl)-2,7,7-trimethyl-5-oxo-N-phenyl-1,4,5,6,7,8-hexahydroquinoline -3-carboxamide (5i)

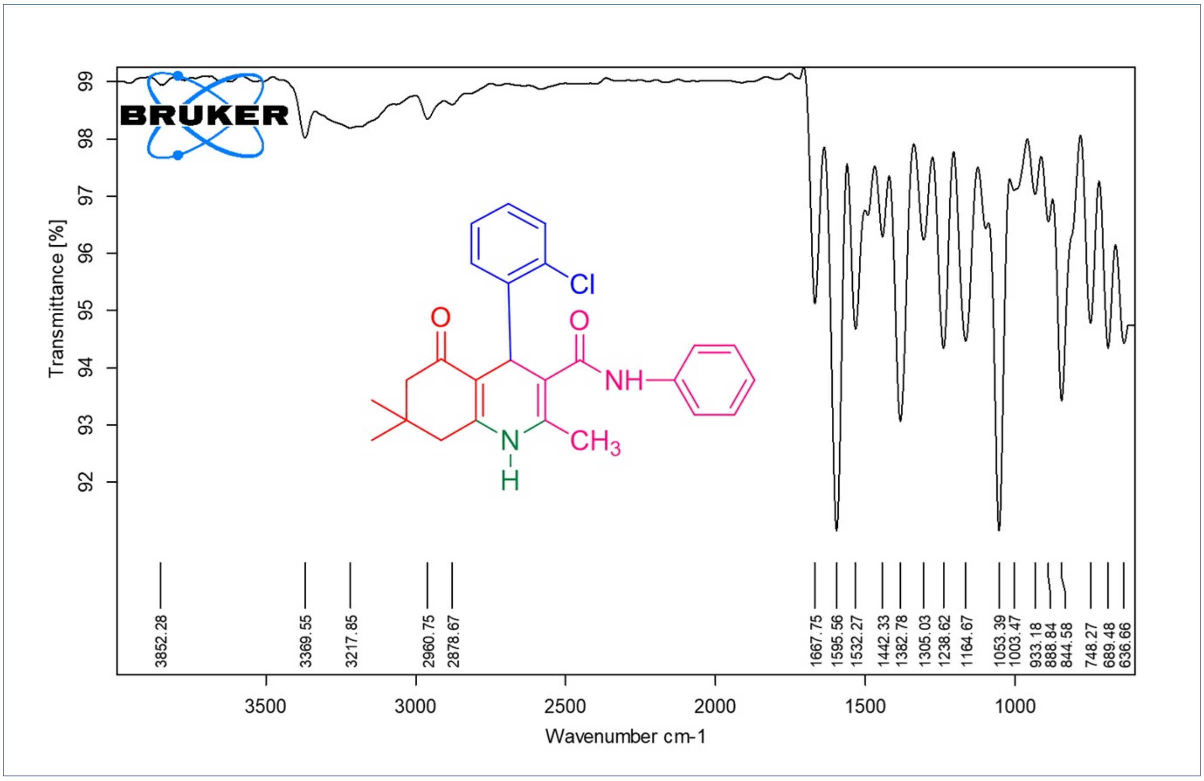

Spectrum 32 : FTIR Spectrum of 4-(2-chlorophenyl)-2,7,7-trimethyl-5-oxo-N-phenyl-1,4,5,6,7,8-hexahydroquinoline -3-carboxamide (5i)

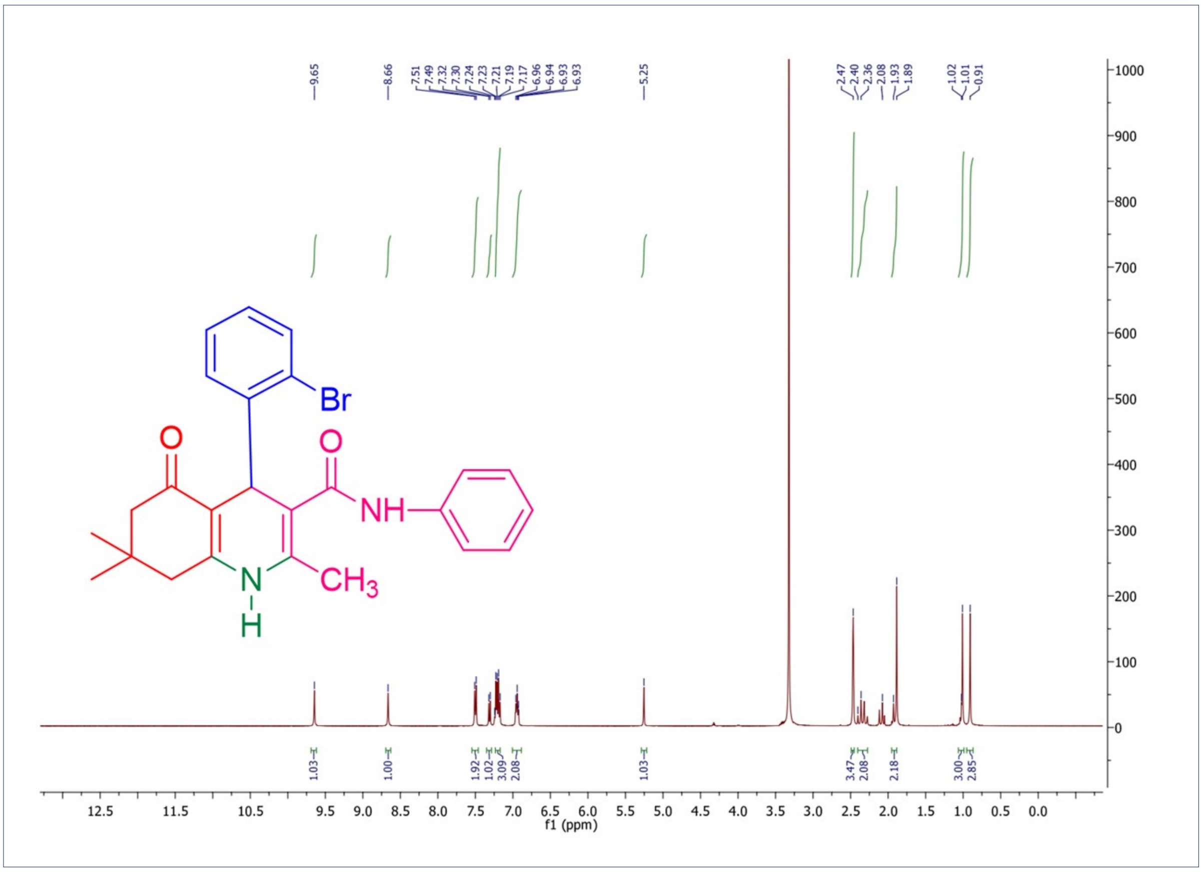

Spectrum 33 : ^{1}H Spectrum of 4-(2-bromophenyl)-2,7,7-trimethyl-5-oxo-N-phenyl-1,4,5,6,7,8-hexahydroquinoline -3-carboxamide (5j)

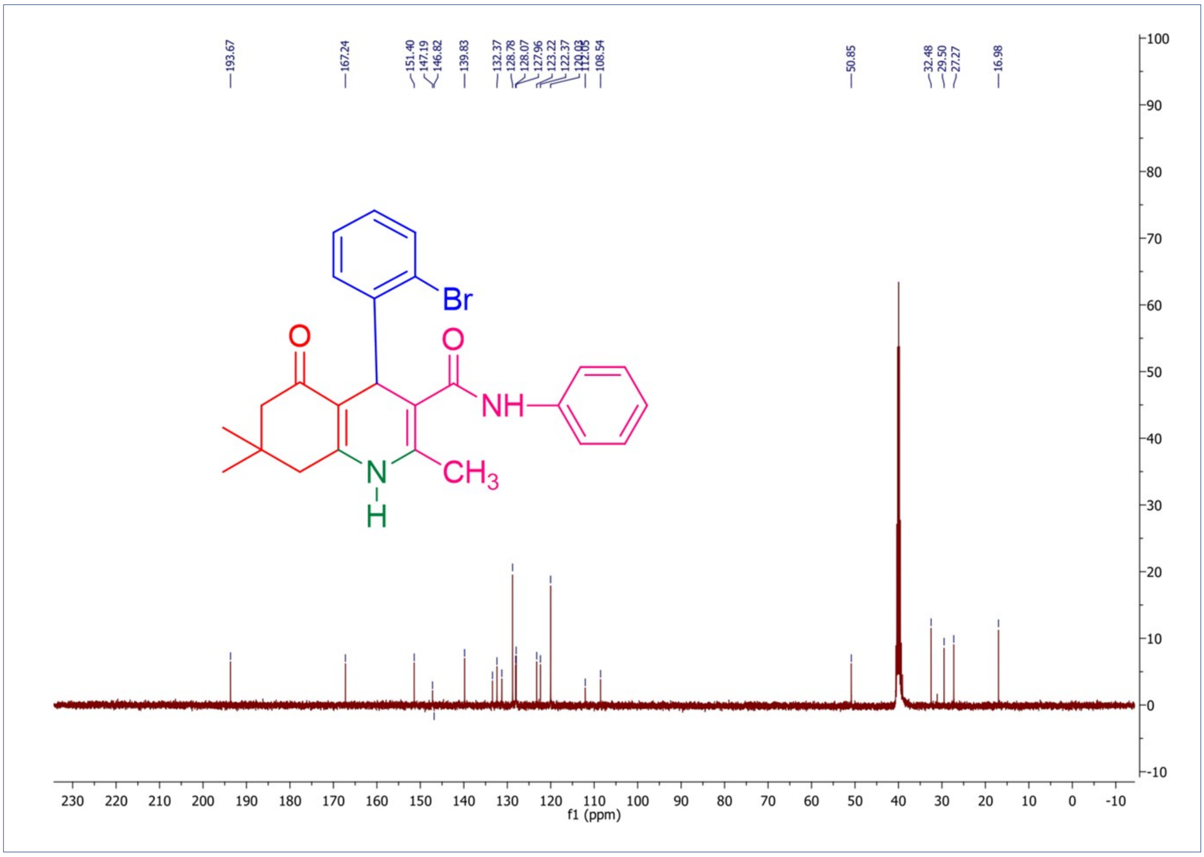

Spectrum 34 : ^{13}C Spectrum of 4-(2-bromophenyl)-2,7,7-trimethyl-5-oxo-N-phenyl-1,4,5,6,7,8-hexahydroquinoline -3-carboxamide (5j)

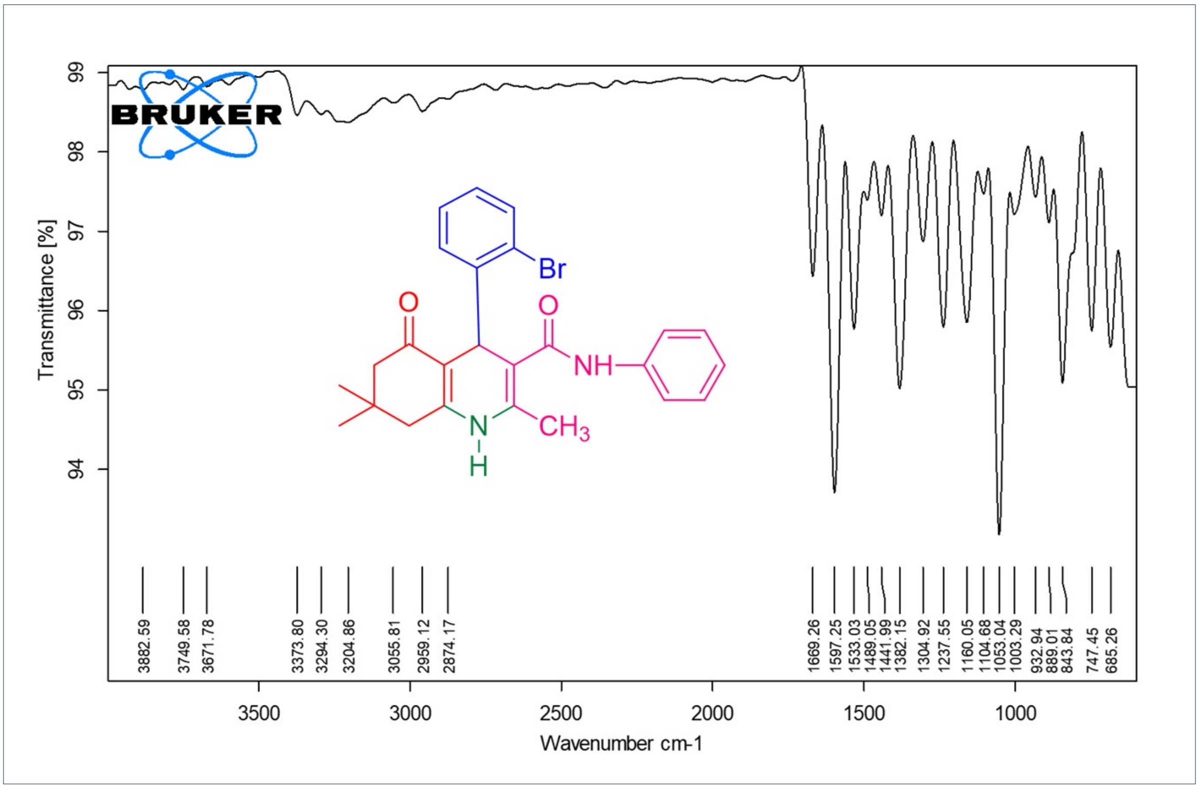

Spectrum 35 : FTIR Spectrum of 4-(2-bromophenyl)-2,7,7-trimethyl-5-oxo-N-phenyl-1,4,5,6,7,8-hexahydroquinoline -3-carboxamide (5j)

Spectral data of represented hexahydroquinoline-3-carboxamide derivatives

4-(phenyl)-2,7,7-trimethyl-5-oxo-N-phenyl-1,4,5,6,7,8-hexahydroquinoline-3-carboxamide (5a)

White solid; M.P. : 245-247 °C

IR (υ_{max} cm^{-1}): 3319 (N-H stretch), 2960 (aliphatic C-H stretch), 1608 (C=O), 1546 (C=O), 1441 (C=C stretch), 753 cm^{-1} (aromatic C-H out of plane bending).

^1H NMR (400 MHz, - DMSO, δ ppm) : 9.48 (s, 1H), 8.65 (s, 1H), 7.50 (d, J = 7.7 Hz, 2H), 7.19 (t, J = 7.9 Hz, 2H), 7.14 (d, J = 4.4 Hz, 3H), 7.03 (dd, J = 8.7, 4.5 Hz, 1H), 6.95 (t, J = 7.4 Hz, 1H), 4.93 (s, 1H), 2.48 – 2.46 (m, 3H), 2.39 – 2.29 (m, 2H), 2.01 (s, 2H), 1.00 (s, 3H), 0.87 (s, 3H).

^{13}C NMR (101 MHz, DMSO, δ ppm) : 194.05 (s), 167.74 (s), 150.91 (s), 147.54 (s), 139.82 (s), 135.36 (s), 128.84 (s), 128.26 (s), 127.83 (s), 126.07 (s), 123.31 (s), 120.06 (s), 111.36 (s), 108.42 (s), 50.86 (s), 32.48 (s), 29.50 (s), 27.21 (s), 17.52 (s).

Mass of $C_{25}H_{26}N_2O_2$, M^+ : 387.36.

4-(4-methoxyphenyl)-2,7,7-trimethyl-5-oxo-N-phenyl-1,4,5,6,7,8-hexahydroquinoline-3-carboxamide (5b)

White solid; M.P. : 246-248 °C

IR (υ_{max} cm^{-1}): 3352 (N-H stretch), 2960 (aliphatic C-H stretch), 1659 (C=O), 1599 (C=O), 1443 (C=C stretch), 754 cm^{-1} (aromatic C-H out of plane bending).

^1H NMR (400 MHz, - DMSO, δ ppm) : 9.48 (s, 1H), 8.64 (s, 1H), 7.54 (s, 2H), 7.23 (s, 2H), 7.09 (d, J = 8.6 Hz, 2H), 6.98 (t, J = 7.2 Hz, 1H), 6.74 (d, J = 8.7 Hz, 2H), 4.91 (s, 1H), 3.66 (s, 3H), 2.51 – 2.49 (m, 3H), 2.38 – 2.28 (m, 2H), 2.05 (s, 2H), 1.03 (s, 3H), 0.91 (s, 3H).

^{13}C NMR (101 MHz, DMSO, δ ppm) : 194.03 (s), 167.79 (s), 157.72 (s), 150.54 (s), 139.88 (d, J = 1.4 Hz), 135.25 (s), 128.82 (d, J = 1.6 Hz), 123.26 (s), 120.03 (s), 113.65 (s), 111.53 (s), 108.75 (s), 55.29 (s), 50.90 (s), 41.18 – 40.86 (m), 40.51 (d, J = 21.0 Hz), 40.20 (s), 39.99 (s), 39.78 (s), 39.57 (s), 39.36 (s), 37.61 (s), 32.47 (s), 29.51 (s), 27.23 (s), 17.53 (s).

Mass of $C_{26}H_{28}N_2O_3$, M^+ : 417.39.

4-(4-methylphenyl)-2,7,7-trimethyl-5-oxo-N-phenyl-1,4,5,6,7,8-hexahydroquinoline-3-carboxamide (5c)

White solid; M.P. : 252-255 °C

IR (υ_{max} cm^{-1}): 3341 (N-H stretch), 2960 (aliphatic C-H stretch), 1656 (C=O), 1598 (C=O), 1441 (C=C stretch), 757 cm^{-1} (aromatic C-H out of plane bending).

^1H NMR (400 MHz, - DMSO, δ ppm) : 9.48 (s, 1H), 8.64 (s, 1H), 7.55 (d, J = 7.6 Hz, 2H), 7.23 (t, J = 7.9 Hz, 2H), 7.05 (d, J = 8.0 Hz, 2H), 6.98 (t, J = 7.3 Hz, 3H), 4.92 (s, 1H), 2.51 –

2.49 (m, 3H), 2.39 – 2.28 (m, 2H), 2.17 (d, J = 9.4 Hz, 3H), 2.04 (s, 2H), 1.03 (s, 3H), 0.91 (s, 3H).

^{13}C NMR (101 MHz, DMSO, δ ppm) : 194.00 (s), 167.76 (s), 150.70 (s), 144.69 (s), 139.87 (s), 135.25 (s), 134.91 (s), 128.84 (d, J = 1.2 Hz), 127.77 (s), 123.26 (s), 120.02 (s), 111.48 (s), 108.58 (s), 50.89 (s), 32.47 (s), 29.52 (s), 27.22 (s), 21.00 (s), 17.52 (s).

Mass of $C_{26}H_{28}N_2O_2$, M^+ : 401.37.

4-(4-bromophenyl)-2,7,7-trimethyl-5-oxo-N-phenyl-1,4,5,6,7,8-hexahydroquinoline-3-carboxamide (5d)

White solid; M.P. : 228-230 °C

IR (υ_{max} cm^{-1}): 3373 (N-H stretch), 2959 (aliphatic C-H stretch), 1669 (C=O), 1597 (C=O), 1441 (C=C stretch), 747 cm^{-1} (aromatic C-H out of plane bending).

^1H NMR (400 MHz, - DMSO, δ ppm) : 9.55 (s, 1H), 8.73 (s, 1H), 7.52 (d, J = 7.8 Hz, 2H), 7.36 (d, J = 8.3 Hz, 2H), 7.22 (t, J = 7.8 Hz, 2H), 7.10 (d, J = 8.3 Hz, 2H), 6.97 (t, J = 7.4 Hz, 1H), 4.93 (s, 1H), 2.48 (d, J = 1.6 Hz, 3H), 2.39 – 2.27 (m, 2H), 2.03 (s, 2H), 1.01 (s, 3H), 0.88 (s, 3H).

^{13}C NMR (101 MHz, DMSO, δ ppm) : 194.05 (s), 167.52 (s), 151.03 (s), 146.87 (s), 139.73 (s), 135.61 (s), 131.11 (s), 130.12 (s), 128.86 (s), 123.39 (s), 120.09 (s), 119.15 (s), 110.87 (s), 108.06 (s), 50.79 (s), 38.25 (s), 32.49 (s), 29.41 (s), 27.24 (s), 17.52 (s), 14.52 (s).

Mass of $C_{25}H_{25}N_2O_2Br$, M^+ : 467.28.

4-(4-fluorophenyl)-2,7,7-trimethyl-5-oxo-N-phenyl-1,4,5,6,7,8-hexahydroquinoline-3-carboxamide (5e)

White solid; M.P. : 205-207 °C

IR (υ_{max} cm^{-1}): 3378 (N-H stretch), 2957 (aliphatic C-H stretch), 1668 (C=O), 1596 (C=O), 1441 (C=C stretch), 746 cm^{-1} (aromatic C-H out of plane bending).

^1H NMR (400 MHz, - DMSO, δ ppm) : 9.51 (s, 1H), 8.68 (s, 1H), 7.50 (d, J = 8.4 Hz, 2H), 7.23 – 7.13 (m, 4H), 6.96 (dd, J = 10.1, 6.6 Hz, 3H), 4.94 (s, 1H), 2.49 – 2.31 (m, 3H), 2.16 – 2.04 (m, 2H), 2.02 (s, 2H), 0.99 (s, 3H), 0.86 (s, 3H).

^{13}C NMR (101 MHz, DMSO, δ ppm) : 194.06 (s), 167.63 (s), 162.07 (s), 159.67 (s), 150.86 (s), 143.71 (s), 139.76 (s), 135.51 (s), 129.53 (d, J = 8.0 Hz), 128.84 (s), 123.36 (s), 120.10 (s), 114.95 (s), 114.74 (s), 111.18 (s), 108.40 (s), 50.82 (s), 37.88 (s), 32.47 (s), 29.43 (s), 27.20 (s), 17.52 (s).

Mass of $C_{25}H_{25}N_2O_2F$, M^+ : 405.37.

4-(4-chlorophenyl)-2,7,7-trimethyl-5-oxo-N-phenyl-1,4,5,6,7,8-hexahydroquinoline-3-carboxamide (5f)

White solid; M.P. : 255-257 °C

IR (υ_{max} cm^{-1}): 3369 (N-H stretch), 2960 (aliphatic C-H stretch), 1667 (C=O), 1595 (C=O), 1442 (C=C stretch), 748 cm^{-1} (aromatic C-H out of plane bending).

^{1}H NMR (400 MHz, - DMSO, δ ppm) : 9.54 (s, 1H), 8.71 (s, 1H), 7.50 (d, J = 7.6 Hz, 2H), 7.20 (t, J = 7.8 Hz, 4H), 7.14 (d, J = 8.5 Hz, 2H), 6.95 (t, J = 7.4 Hz, 1H), 4.93 (s, 1H), 2.48 – 2.30 (m, 3H), 2.14 – 2.02 (m, 2H), 2.00 (d, J = 16.4 Hz, 2H), 0.99 (s, 3H), 0.86 (s, 3H).

^{13}C NMR (101 MHz, DMSO, δ ppm) : 194.07 (s), 167.54 (s), 151.03 (s), 146.44 (s), 139.73 (s), 135.61 (s), 130.62 (s), 129.69 (s), 128.86 (s), 128.20 (s), 123.39 (s), 120.10 (s), 110.93 (s), 108.12 (s), 50.79 (s), 38.16 (s), 32.48 (s), 29.41 (s), 27.21 (s), 17.52 (s).

4-(4-nitrophenyl)-2,7,7-trimethyl-5-oxo-N-phenyl-1,4,5,6,7,8-hexahydroquinoline-3-carboxamide (5g)

Orange yellow solid; M.P. : 207-210 °C

IR (υ_{max} cm^{-1}): 3377 (N-H stretch), 2960 (aliphatic C-H stretch), 1666 (C=O), 1595 (C=O), 1442 (C=C stretch), 745 cm^{-1} (aromatic C-H out of plane bending).

^{1}H NMR (400 MHz, - DMSO, δ ppm) : 9.61 (s, 1H), 8.84 (s, 1H), 8.06 (d, J = 8.7 Hz, 2H), 7.49 (d, J = 7.7 Hz, 2H), 7.40 (d, J = 8.7 Hz, 2H), 7.20 (t, J = 7.8 Hz, 2H), 6.95 (d, J = 7.4 Hz, 1H), 5.06 (s, 1H), 2.48 – 2.45 (m, 3H), 2.34 (d, J = 14.5 Hz, 2H), 2.04 (s, 2H), 1.00 (s, 3H), 0.85 (s, 3H).

^{13}C NMR (101 MHz, DMSO, δ ppm) : 194.07 (s), 167.24 (s), 154.93 (s), 151.50 (s), 146.09 (s), 139.60 (s), 136.24 (s), 129.08 (s), 128.88 (s), 123.57 (d, J = 13.7 Hz), 120.13 (s), 110.16 (s), 107.54 (s), 50.67 (s), 32.50 (s), 29.33 (s), 27.22 (s), 17.57 (s).

4-(4-hydroxyphenyl)-2,7,7-trimethyl-5-oxo-N-phenyl-1,4,5,6,7,8-hexahydroquinoline-3-carboxamide (5h)

White solid; M.P. : > 300 °C

IR (υ_{max} cm^{-1}): 3388 (N-H stretch), 2957 (aliphatic C-H stretch), 1608 (C=O), 1521 (C=O), 1446 (C=C stretch), 749 cm^{-1} (aromatic C-H out of plane bending).

^{1}H NMR (400 MHz, - DMSO, δ ppm) : 9.39 (s, 1H), 9.00 (s, 1H), 8.58 (s, 1H), 7.50 (d, J = 7.7 Hz, 2H), 7.19 (t, J = 7.9 Hz, 2H), 6.98 – 6.89 (m, 3H), 6.52 (d, J = 8.5 Hz, 2H), 4.80 (s, 1H), 2.49 – 2.41 (m, 3H), 2.29 (dd, J = 36.1, 16.8 Hz, 2H), 2.06 (d, J = 13.2 Hz, 2H), 0.99 (s, 3H), 0.87 (s, 3H).

^{13}C NMR (101 MHz, DMSO, δ ppm) : 194.09 (s), 167.87 (s), 155.68 (s), 150.44 (s), 139.85 (s), 138.25 (s), 135.11 (s), 128.79 (d, J = 9.0 Hz), 123.27 (s), 120.03 (s), 115.02 (s), 111.66 (s), 108.89 (s), 50.92 (s), 37.52 (s), 32.46 (s), 29.52 (s), 27.18 (s), 17.51 (s).

4-(2-chlorophenyl)-2,7,7-trimethyl-5-oxo-N-phenyl-1,4,5,6,7,8-hexahydroquinoline-3-carboxamide (5i)

White solid; M.P. : 228-230 °C

IR (υ_{max} cm^{-1}): 3369 (N-H stretch), 2960 (aliphatic C-H stretch), 1667 (C=O), 1595 (C=O), 1442 (C=C stretch), 748 cm^{-1} (aromatic C-H out of plane bending).

^{1}H NMR (400 MHz, - DMSO, δ ppm) : 9.63 (s, 1H), 8.67 (s, 1H), 7.49 (d, J = 7.8 Hz, 2H), 7.20 (ddd, J = 13.0, 7.6, 3.2 Hz, 4H), 7.14 (s, 1H), 7.03 (dd, J = 10.9, 4.2 Hz, 1H), 6.94 (t, J = 7.3 Hz, 1H), 5.31 (s, 1H), 2.47 (s, 2H), 2.38 – 2.27 (m, 2H), 1.91 (s, 3H), 1.02 (d, J = 6.1 Hz, 3H), 0.91 (s, 3H).

^{13}C NMR (101 MHz, DMSO, δ ppm) : 193.68 (s), 167.31 (s), 151.51 (s), 145.29 (s), 139.85 (s), 133.94 (s), 131.82 (s), 131.16 (s), 129.13 (s), 128.79 (s), 127.66 (s), 127.42 (s), 123.23 (s), 119.99 (s), 111.60 (s), 108.18 (s), 50.82 (s), 36.60 (s), 32.47 (s), 29.51 (s), 27.27 (s), 17.05 (s).

4-(2-bromophenyl)-2,7,7-trimethyl-5-oxo-N-phenyl-1,4,5,6,7,8-hexahydroquinoline-3-carboxamide (5j)

White solid; M.P. : 241-242 °C

IR (υ_{max} cm^{-1}): 3373 (N-H stretch), 2959 (aliphatic C-H stretch), 1669 (C=O), 1597 (C=O), 1441 (C=C stretch), 747 cm^{-1} (aromatic C-H out of plane bending).

^{1}H NMR (400 MHz, - DMSO, δ ppm) : 9.65 (s, 1H), 8.66 (s, 1H), 7.50 (d, J = 8.1 Hz, 2H), 7.31 (d, J = 7.9 Hz, 1H), 7.23 – 7.17 (m, 3H), 6.94 (dd, J = 9.3, 5.0 Hz, 2H), 5.25 (s, 1H), 2.47 (s, 3H), 2.38 (d, J = 16.8 Hz, 2H), 1.93 (s, 2H), 1.02 (d, J = 5.2 Hz, 3H), 0.91 (s, 3H).

^{13}C NMR (101 MHz, DMSO, δ ppm) : 193.67 (s), 167.24 (s), 151.40 (s), 147.19 (s), 139.83 (s), 133.41 (s), 132.37 (s), 131.25 (s), 128.78 (s), 128.01 (d, J = 11.0 Hz), 123.22 (s), 122.37 (s), 120.03 (s), 112.05 (s), 108.54 (s), 50.85 (s), 32.48 (s), 29.50 (s), 27.27 (s), 16.98 (s).
